# The effectiveness of mental health interventions involving non-specialists and digital technology in low-and middle-income countries – a systematic review

**DOI:** 10.1186/s12889-023-17417-6

**Published:** 2024-01-03

**Authors:** Kalpani Wijekoon Wijekoon Mudiyanselage, Karina Karolina De Santis, Frederike Jörg, Maham Saleem, Roy Stewart, Hajo Zeeb, Heide Busse

**Affiliations:** 1 Faculty 11 Human and Health Sciences, University of Bremen, Bremen, Germany; 2 Department of Prevention and Evaluation, Leibniz Institute for Prevention Research and Epidemiology- BIPS, Bremen, Germany; 3 Department of Psychiatry, Interdisciplinary Center Psychopathology and Emotion regulation, University of Groningen, University Medical Center Groningen, Groningen, the Netherlands; 4 Department of Education and Research, Friesland Mental Health Care Services, Leeuwarden, the Netherlands; 5 Department of Health Sciences, Community & Occupational Medicine, University of Groningen, University Medical Center Groningen, Groningen, the Netherlands; 6 Leibniz ScienceCampus Digital Public Health Bremen, Bremen, Germany

**Keywords:** Mental health, Non-specialists, Task-sharing, Digital technologies, Low-and middle-income countries, Systematic review, Interventions, Mental healthcare

## Abstract

**Background:**

Combining non-specialists and digital technologies in mental health interventions could decrease the mental healthcare gap in resource scarce countries. This systematic review examined different combinations of non-specialists and digital technologies in mental health interventions and their effectiveness in reducing the mental healthcare gap in low-and middle-income countries.

**Methods:**

Literature searches were conducted in four databases (September 2023), three trial registries (January–February 2022), and using forward and backward citation searches (May–June 2022). The review included primary studies on mental health interventions combining non-specialists and digital technologies in low-and middle-income countries. The outcomes were: (1) the mental health of intervention receivers and (2) the competencies of non-specialists to deliver mental health interventions. Data were expressed as standardised effect sizes (Cohen’s d) and narratively synthesised. Risk of bias assessment was conducted using the Cochrane risk-of-bias tools for individual and cluster randomised and non-randomised controlled trials.

**Results:**

Of the 28 included studies (*n* = 32 interventions), digital technology was mainly used in non-specialist primary-delivery treatment models for common mental disorders or subthreshold symptoms. The competencies of non-specialists were improved with digital training (d ≤ 0.8 in 4/7 outcomes, *n* = 4 studies, 398 participants). The mental health of receivers improved through non-specialist-delivered interventions, in which digital technologies were used to support the delivery of the intervention (d > 0.8 in 24/40 outcomes, *n* = 11, 2469) or to supervise the non-specialists’ work (d = 0.2–0.8 in 10/17 outcomes, *n* = 3, 3096). Additionally, the mental health of service receivers improved through digitally delivered mental health services with non-specialist involvement (d = 0.2–0.8 in 12/27 outcomes, *n* = 8, 2335). However, the overall certainty of the evidence was poor.

**Conclusion:**

Incorporating digital technologies into non-specialist mental health interventions tended to enhance non-specialists’ competencies and knowledge in intervention delivery, and had a positive influence on the severity of mental health problems, mental healthcare utilization, and psychosocial functioning outcomes of service recipients, primarily within primary-deliverer care models. More robust evidence is needed to compare the magnitude of effectiveness and identify the clinical relevance of specific digital functions. Future studies should also explore long-term and potential adverse effects and interventions targeting men and marginalised communities.

**Supplementary Information:**

The online version contains supplementary material available at 10.1186/s12889-023-17417-6.

## Background

### The mental healthcare gap

Despite the availability of evidence-based treatments for mental disorders, most of those in need of care do not receive the adequate type and amount of care in a timely manner, which has been described as the mental healthcare (MHC) gap [1, 2]. Compared to high-income countries, where 36–50% of people with a mental disorder are estimated to be undertreated, this amounts to 76–85% in low-and middle-income countries (LMICs), which can be explained by the general lack of resources and high stigmatization of mental disorders in LMICs [2, 3].

### Strategies to reduce the mental healthcare gap

To decrease the MHC gap in resource-poor settings such as LMIC, researchers have stressed the importance of using trained and supervised non-specialists to deliver mental health promotion, mental illness prevention, and treatment activities [4–6]. Non-specialist MHC workers have not received specialised training or tertiary education in mental health-related fields. This definition includes lay people from the community, primary care physicians, or other health workers not specialised in mental health and excludes MHC specialists, such as psychiatrists, neurologists, psychologists, psychiatric nurses, or mental health social or occupational workers [7, 8].

Barnett and colleagues [7] have set the groundwork for non-specialist-led mental health interventions by proposing a conceptual framework that differentiates between *the outreach/navigator model*, *the auxiliary care model*, and *the task-shifting model* [7, 9]. In the outreach/navigator model, the non-specialist is concerned with bridging the gap between the community and care provider by raising awareness of mental health, screening for mental disorders and providing guidance to treatment pathways [7]. In the auxiliary care model, non-specialists involved in treatment may act as auxiliary workers, meaning they assist the specialist who provides treatment by, for example, promoting the treatment and medication adherence of patients. Lastly, non-specialists may be used in the task-shifting model, which differentiates between the stepped-care approach and the primary-deliverer care approach, depending on the involvement of the specialist in care delivery. In the stepped-care approach non-specialists provide the least intensive care available and refer patients to a specialist if required. In the primary-deliverer care approach, the non-specialist acts as the sole treatment deliverer [7].

Several randomized controlled trials (RCTs) have shown that particularly the outreach model and the task-shifting models hold the potential to increase mental health awareness and decrease the symptom burden of people with mental health complaints in LMICs [8, 10–12]. However, utilizing non-specialists to bridge the MHC gap still requires appropriate training and ongoing support to ensure the safety of patients and non-specialists [13, 14]. The most pressing challenge is, however, finding a suitable way to support non-specialists in LMICs, considering the resource scarcity.

One potential solution to support non-specialised MHC workers may be through the use of digital technologies [5, 15]. Technology-based devices are devices with a digital component, such as mobile phones, smartphones, telepsychiatry, wearables or sensors, online platforms, or mobile applications [15]. Access to technology-based devices, such as mobile phones, has increased rapidly in the past years in LMICs [16, 17]. By the end of 2021, half of the population residing in LMICs used mobile internet, implying that digital technology may hold the potential to address health priorities in countries with limited human workforce [18].

According to a framework by Agarwal et al. [19], digital technologies can adopt three prominent roles when used to support healthcare workers: *1. Training and competence building*, *2. Supporting the delivery of health interventions*, and *3. Supervising and supporting retention.* Other researchers found that digital technology was used by front-line health workers to receive education on treatment guidelines, for data collection and reporting, improvement of communication, alerts and reminders, client education, emergency referrals, supervision, enhancing motivation and maintaining competence [19]. To the best of our knowledge, no review has yet been undertaken to systematically summarize the evidence of interventions involving mental health non-specialists and digital technologies within the context of reducing the MHC gap in LMICs.

### Aims and research questions

Given the lack of evidence on the effectiveness of interventions combining mental health non-specialist models with digital technology in reducing the MHC gap, this systematic review has the following aims:

1. To assess how non-specialists and digital technologies are combined in mental health interventions.
2. To assess the effectiveness of these interventions in reducing the MHC gap in LMICs.

In particular, this review attempts to answer the following research questions (RQ):

1. How are non-specialists and digital technologies combined in mental health interventions?
2. Are digital training interventions effective for non-specialists?
3. Are mental health interventions delivered by non-specialists who are supported by digital technologies effective for the service receivers?
4. Are digitally delivered interventions with additional non-specialist involvement effective for service receivers?
5. Are digital supervision tools effective for non-specialists?

## Methods

A protocol for this review, including the corresponding amendments, was registered before the commencement at the International prospective register of systematic reviews, PROSPERO (Registration number: CRD42021293016). The conduct and reporting of this review adhere to the Preferred Reporting Items for Systematic Reviews and Meta-analysis (PRISMA 2020) guidelines [20] [Additional file 1] and guidance by AMSTAR 2 (A Measurement Tool to Assess Systematic Reviews) [21] [Additional file 2].

### Eligibility criteria

Randomised controlled trials (RCTs) and non-randomised controlled trials (NRCTs), pilot, and feasibility studies were included. The reason for including this broad selection of study designs is that especially population-level interventions are considered to be hard to randomise and because, next to the effectiveness of such interventions, we were interested in the way digital technologies and non-specialists can be combined in mental health interventions [22]. The following PICO characteristics had to be fulfilled for studies to be eligible for inclusion: (1) Population: People (of any age) who are non-specialists or service receivers (people receiving the intervention of interest) residing in LMIC, defined by the World Bank data [23]. (2) Intervention: Mental health services combining non-specialised MHC workers with digital technology that promote mental health, prevent or treat mental illness [15]. (3) Control: Care as usual, baseline outcomes in studies with only one group, interventions that only included non-specialised MHC workers without technology-based support, interventions that only included digital technology without non-specialists involved. (4) Outcome: Any outcomes related to mental health promotion/prevention (i.e., psychosocial functioning outcomes), mental illness treatment (i.e., treatment seeking behaviour, (severity of) mental illness burden and adverse events), and the competencies of non-specialists in delivering the intervention (i.e., knowledge, competence scores). For RCTs and NRCTs, only primary outcomes were included. For (N)RCTs that do not specify a primary outcome and for pilot and feasibility studies, any outcome of interest was selected. This is because the primary outcomes in pilot and feasibility studies usually did not correspond with the interests of this review. Reviews, comments (non-primary studies), conference papers, dissertations, and studies not published in English were excluded from the review.

### Search strategy and study selection

#### Study sources

The search for eligible studies was based on three study sources: First, four bibliographic databases (PubMed, Psychological Information Database (PsychINFO), Cumulative Index to Nursing and Allied Health Literature (CINAHL), Web of Science) were searched for relevant primary studies and protocols from their inception until 18.09.2023. Second, trial registries were searched for protocols of suitable intervention studies to identify any additional studies not picked up by the initial search. The following trial registries were searched: International Standard Randomised Controlled Trial Number (ISRCTN), International Clinical Trials Registry Platform (ICTRP) by the WHO, and Clinical Trials Registry- India (CTRI) using similar search terms as within the bibliographic search. Third, backward and forward citation chaining of the included studies was conducted to identify further eligible studies.

#### Search strategy development

The search strategy was developed with support from a research librarian and included keywords and vocabulary terms for the following four concepts: 1. LMIC, 2. Non-specialists, 3. Digital technology, 4. Mental health. The search syntax for PubMed is presented elsewhere [Additional file 3].

#### Search conduct

First, three consecutive searches were performed in December 2021 (database search), January and February 2022 (publications of protocols from databases and grey literature), and May and June (forward and backward citation chaining of included articles) 2022 by the first author. An additional database search was performed to include studies that were published between the December 2021 and September 2023.

#### Study selection

**Fig. 1:**
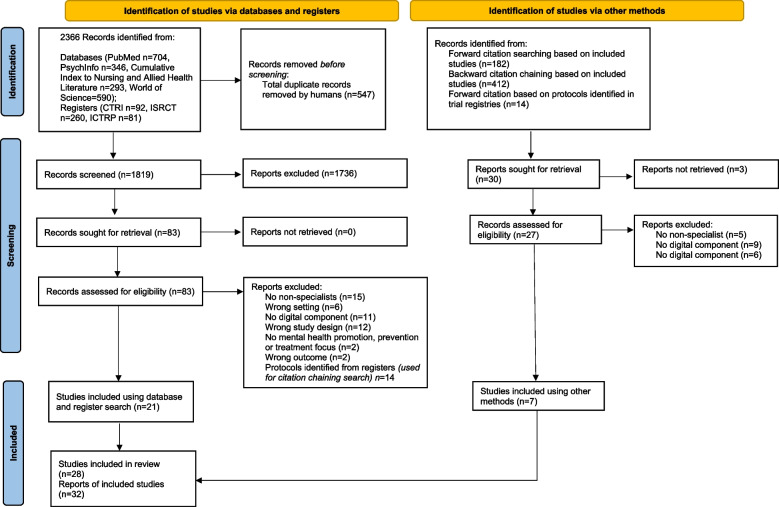
PRISMA flow diagram. Notes: This diagram was derived from the PRISMA 2020 statement [20]

The search results were first extracted to EndNote to remove duplicates. The remaining studies were then imported into the Covidence review software for screening. Two reviewers independently conducted the title, abstract and full-text screening and resolved any discrepancies by discussion. Following the study assessment, 28 out of 2413 studies reporting on 32 interventions were included in this review (Fig. 1). A list of excluded studies with reasons for exclusion in the full-text screening is reported elsewhere [Additional file 4].

### Data extraction

Data from all included studies were extracted into a self-developed Excel sheet. Data items included bibliographic information (publication year and country), study design, characteristics of participants (health status and demographics, including biological sex or gender information), interventions, control conditions, and outcomes (i.e., outcome type, methods to measure the outcomes, and results). For studies focusing on non-specialists, the outcomes from the latest assessment time-point were obtained to provide insights into the long-term impact of digital training. For studies focusing on the service receiver and in which the primary outcome was not defined or did not specify the assessment time-point, the results were obtained for all different assessment time points. If available, outcomes using the intention-to-treat (ITT) analysis were preferred over the complete case analysis (CCA). In case of missing or unclear data, the study authors were contacted. Two reviewers independently extracted data from all studies and resolved any discrepancies by discussion.

### Study quality

The risk of bias was assessed using RoB2 (Version 2 of the Cochrane risk-of-bias tool for individual and cluster RCTs [24] and ROBINS-I (Risk Of Bias In NRCTs of Interventions) (ROBINS-I) [25]. We assumed that pilot and feasibility studies generated results with a high risk of bias given small sample sizes and thus unrepresentative results. Two reviewers appraised each study independently and resolved any discrepancies by discussion. The overall certainty of the evidence was evaluated based on the GRADE guidelines and using the recommendations for certainty rating in narrative synthesis [26, 27].

### Data synthesis

All studies were grouped according to the non-specialist models [7] and the m-health functions model [19] to describe how mental health interventions have combined digital technologies and non-specialists. Following the Synthesis without Meta-analysis (SWiM) guidelines, a narrative synthesis was conducted [28]. If possible, the effect sizes (standardised mean difference, Cohen’s d) were calculated using Excel and all used formulae are reported elsewhere [Additional file 5] [29]. Effect sizes were calculated based on either difference in the outcome at follow-up, pre-post change of the outcome between the intervention and control group, or pre-post change only in the intervention group without a control condition. The baseline sample size and a correlation coefficient of r = 0.5 were used in the calculations. The effect sizes were interpreted small (d < 0.2), medium (d = 0.2–0.8), or large (d > 0.8) [29]. In four studies [30–33], more than one intervention was investigated. For these, we separately calculated the effect size of both intervention relative to the baseline values [30, 32, 33] and waitlist control group [31]. In addition, different effect sizes comparing each endpoint with the same baseline were computed for studies where multiple assessment points of the outcome were extracted. To address the potential ambiguity caused by varying assessment tools, we chose to present absolute effect sizes. We defined a favourable outcome as one in which the intervention led to lower severity of mental problem outcomes (compared to the control group or baseline outcomes) and higher psychosocial functioning and MHC use outcomes (compared to the control group or baseline outcomes) or when the change in outcomes from pre- to post-intervention was greater in the intervention group than in the control group.

## Results

### Study characteristics

Of the *n* = 28 included studies reporting on *n* = 32 interventions and published in peer-reviewed journals between 2013 and 2023, four focused on non-specialists and *n* = 24 focused on service receivers (Table 1). In total, *n* = 13 were RCTs, four were NRCTS, six were feasibility studies, and five were pilot studies (Table 2). The included interventions were conducted in China (*n* = 6), India (*n* = 5), Brazil (*n* = 5), Pakistan (*n* = 3), Thailand (*n* = 3), Zimbabwe (*n* = 3), Peru (*n* = 2), Kenya (*n* = 1), Indonesia (*n* = 1), Egypt (*n* = 1), Turkey (*n* = 1), Korea (*n* = 1), Malaysia (*n* = 1) and mostly in urban settings (*n* = 21 interventions). In some cases, the same intervention was conducted in different trial settings (Table 1).

**Table 1:** Participant characteristics

Author, reference	Type of non-specialist/ service receivers (mental health assessment tools)	Sample size (n) at baseline^a^	Age in years (mean, SD or range)	Gender (woman %)	Setting
***Digital training of non-specialists***					
Rahman [34]	Lady health workers	Intervention *n* = 40; Control *n* = 40	Intervention mean = 36, SD = 7; Control mean = 35, SD = 8	100%^b^	Pakistan, urban+rural
Muke [30]	ASHA, ASHA Facilitator, Multi-Purpose Health workers	Intervention 1 *n* = 14	Mean = 36	79%^b^	India, rural+urban
		Intervention 2 *n* = 14	Mean = 38	79%^b^	
Nisar [35]	Enrolled nurses at nursing school	Intervention *n* = 50; Control *n* = 50	Intervention mean = 20, SD = 2; Control mean = 19, SD = 2	Intervention = 85%^b^; Control = 70%^b^	China, urban
Pereira [31]	Teachers	Intervention 1 *n* = 52	Intervention 1 mean = 40, SD = 10	100%^b^	Brazil, urban
		Intervention 2 *n* = 32	Intervention 2 mean = 43, SD = 11;	97%^b^	
***Digital support for non-specialist-delivered interventions***					
Maulik [36]	Tribe communities screened positive for CMD symptoms (PHQ-9 ≥ 10, GAD≥10) by ASHAs	*n* = 238	Mean = 44, SD = 15	71%^b^	India, rural
Maulik [37]	People screened positive for CMD symptoms (PHQ-9 ≥ 10, GAD≥10) by ASHAs	*n* = 900	Mean = 48–53	70%^b^	
Doukani [38]	People with CMD symptoms (SSQ ≥ 9)	*n* = 60	18–51 or older	63%^b^	Kenya, urban
Dambi [32]	People with CMD symptoms (SSQ ≥ 9)	Intervention 1 *n* = 45	Mean = 25, SD = 6	66%^d^	Zimbabwe, urban+ rural
		Intervention 2 *n* = 32	Mean = 23, SD = 4		
Chibanda [39]	People with CMD symptoms and/or suicidal ideation but without suicide intent (SSQ ≥ 9)	Intervention *n* = 286; Control *n* = 287	Intervention mean = 33, SD = 11; Control mean = 37, SD = 13	Intervention = 89%^c^ Control = 84%^c^	Zimbabwe, urban
Ross [40]	Pregnant women (≤28 gestation weeks) with HIV	Intervention *n* = 20; Control *n* = 20	Intervention mean = 26, SD = 5; Control mean = 27, SD = 6	100%^b^	Thailand, urban
Ebrahem [41]	Parents of school children during the COVID-pandemic	*n* = 220 couples	Mean = 43–44	50%^b^	Egypt, urban+ rural
Scazufca [42]	Elderly people with depressive symptoms (PHQ-9 ≥ 10)	Intervention *n* = 33; Control *n* = 25	60–70 or older	Intervention = 73%^c^; Control = 80%^c^	Brazil, urban
Garg [43]	People with a (probable) mental disorder (GHQ-12 ≥ 4, AUDIT ≥15, clinical judgement)	*n* = 161	18–65 or older	63%^d^	India, urban and rural
Liu [44]	People with spinal cord injury	Intervention *n* = 49; Control *n* = 49	Intervention mean = 40, SD = 12; Control mean = 43, SD = 12	17%^d^	China, urban
Öztoprak [45]	Women at postnatal stage	Intervention *n* = 21; Control *n* = 23	Intervention mean = 23, SD = 3; Control mean = 24, SD = 3	100%^b^	Turkey, urban
***Digitally delivered treatment with non-specialist involvement***					
Hong [46]	Elderly people with mild depressive symptoms (KGDS ≥5)	Intervention *n* = 21; Control = 23	Intervention mean = 76, SD = 6; Control mean = 77, SD = 6	64^c^	Korea, urban
Hanita [47]	People undergoing a coronary artery bypass grafting surgery	Intervention *n* = 23, Control *n* = 21	Intervention mean = 57, SD = 12; Control mean = 58, SD = 12	20%^b^	Malaysia, urban
Xu [48]	People with substance abuse disorder (SCID-5)	Intervention *n* = 20; Control *n* = 20	Intervention mean = 47, SD = 9; Control mean = 45, SD = 11	Intervention = 20%^c^; Control = 25%^c^	China, urban
Rodriguez [49]	University students with at least mild depression and/or anxiety (self-report)	*n* = 27	Mean = 23, SD = 3	81%^c^	China, urban
Anttila [33]	School children	Intervention 1 *n* = 54	Mean = 16, SD = 1	64%^b^	Thailand, urban
		Intervention 2 *n* = 55	Mean = 16, SD = 1	55%^b^	
Menezes [50]	People with diabetes or hypertension + depressive symptoms (PHQ-9 ≥ 10)	Brazil trial: *n* = 21, Peru trial 1: *n* = 21, Peru trial 2: *n* = 24	All trials: 21–61 or older	62–76%^b^	Brazil & Peru, urban
Zhou [51]	Women with breast cancer	Intervention *n* = 66; Control *n* = 66	Intervention mean = 45, SD =7.89; Control mean = 44, SD = 7.32	100%^b^	China, urban
Gonsalves, 2021 [52]	School children with psychological stress (self-report)	*n* = 248	Mean = 16	50%^b^	India, urban
Arjadi [53]	People with major depressive disorder (PHQ-9 ≥ 10, SCID-5)	Intervention *n* = 159; Control *n* = 154	Intervention mean = 24, SD = 5; Control mean = 25, SD = 5	81%^c^	Indonesia, urban+rural
Araya [54]	Patients with diabetes and/or hypertension + depressive symptoms (PHQ-9 ≥ 9)	Brazil trial: Intervention *n* = 440; Control *n* = 440, Peru trial: Intervention *n* = 217; Control *n* = 215	All trials: 21–61 or older	Intervention = 86%^d^; Control = 77–87%^d^	Brazil&Peru, urban
***Digital supervision of non-specialists***					
Khan, 2019 [55]	Woman with psychological distress (GHQ > 2, WHO-DAS > 16)	Intervention *n* = 59; Control *n* = 60	Both groups: 18–46 or older	100%^b^	Pakistan, rural
Rahman, 2019 [56]	Woman with psychological distress (GHQ ≥3, WHO-DAS ≥ 17)	Intervention *n* = 306; Control *n* = 306	Intervention mean = 37, SD = 11, Control mean = 35, SD = 9	100%^b^	
Chen, 2022 [57]	Elderly people with depressive symptoms + hypertension (PHQ-9 ≥ 10, SIS< 3)	Intervention *n* = 1232; Control *n* = 1133	Both groups: 60–69 or older	Intervention = 67%^c^; Control = 66%^c^	China, rural

^a^The definition of the intervention and control groups are indicated in table. Baseline numbers refer to those participants who did the baseline assessments in the respective studies. ^b^These studies used solely the terms “gender”, or “women and men” or “husband” and “wife”; ^c^These studies solely used the terms “sex”, or “female” or “male”; ^d^These studies used the terms “sex”, “gender” interchangeably. Abbreviations: *ASHA* Accredited Social Health Activist, *CMD* common mental disorders, *PHQ-9* Patient Health Questionnaire-9, *GAD* Generalised anxiety disorder questionnaire, *SRQ-20* self-reporting questionnaire 20-item, *SSQ* Shona Symptom Questionnaire, *GHQ − 12* Global Health Questionnaire, *AUDIT* Alcohol Use Disorder Test, *KGDS* Korean Version of the Geriatric Depression Scale – Short Form, *SCI* Clinical Interview based on the Statistical Manual of Mental Disorders - 5, *SIS* Six Item Screener for Intact Cognitive Functioning, *WHO-DAS* WHO disability assessment schedule

**Table 2:** Study design and intervention characteristics

Author, year, reference	Sources of study funding reported^a^	Study design^b^	Interventions	Outcomes definition
***Digital training of non-specialists***				
Rahman, 2019 [34]	Yes	RCT^c^ - The individual pre-test post-test control group design	Digital training with face-to-face support vs. conventional training	Competence^e^
Muke, 2020 [30]	Yes	Pilot study - The individual multiple treatment and control pre-test design	Intervention 1: Digital training; follow-up vs. baseline	Competence^f^
		Pilot study. The individual multiple treatment and control pre-test design	Intervention 2: Digital training + remote support; follow-up vs. baseline	Competence^f^
Nisar, 2022 [35]	Yes	RCT^c^ - The individual pre-test post-test control group design	Digital training with face-to-face support vs. conventional training	Competence^e^
Pereira, 2015 [31]	Yes	RCT^c^ - The cluster multiple treatment and control pre-test design	Intervention 1: Web-based interactive education vs. Wait-list control	Knowledge about child mental health^g^
		RCT^c^ - The cluster multiple treatment and control pre-test design	Intervention 2: Text-and video-based education vs. Wait-list control	Knowledge about child mental health^g^
***Digital support for non-specialists***				
Maulik, 2017 [36]	Yes	NRCT^d^ - One-group pre-test post-test design	The Systematic Medical Appraisal Referral and Treatment intervention; follow-up vs. baseline	Mental health service use^h^
Maulik, 2020 [37]	No	NRCT^d^ - One-group pre-test post-test design	The Systematic Medical Appraisal Referral and Treatment intervention; follow-up vs. baseline	Mental health service use^h^
Doukani, 2022 [38]	Yes	Pilot study - One-group pre-test post-test	The Inuka couaching app intervention; follow-up vs. baseline	Symptoms of common mental disorders^i^, Depressive symptoms^j^,Anxiety symptoms^k^
Dambi, 2022 [32]	No	Feasibility trial- The individual alternative treatment control group design	Intervention 1: The Inuka coaching app intervention; follow-up vs. baseline	Symptoms of common mental disorders^l^, Depressive symptoms^j^, Anxiety symptoms^k^, Disability and functioning^m^, Quality of life^o^
			Intervention 2: Friendship-bench Whatsapp intervention; follow-up vs. baseline	
Chibanda, 2016 [39]	Yes	RCT^c^ - The cluster alternative treatment control group design	The friendship-bench intervention vs. enhanced usual care	Symptoms of common mental disorders^l^
Ross, 2013 [40]	Yes	RCT^c^ - The individual pre-test post-test control group design	Telephone counselling intervention vs. regular prenatal care and HIV education	Depressive symptoms^p^
Ebrahem, 2023 [41]	No	NRCT^d^ – One group pre-test post-test design	Telehealth nursing intervention; follow-up vs. baseline	Proportion of people with depression (normal vs. mild-severe), anxiety (normal vs. mild-extremely severe), stress (normal vs. mild-severe)^q^
Scazufca, 2019 [42]	Yes	Feasibility trial - The cluster alternative treatment control group design	Psychosocial intervention vs. enhanced usual care	Depressive symptoms^j^, Quality of life^o^, Disability^r^
Garg, 2022 [43]	Yes	Feasibility study - One-group pre-test post-test	IMproving Access through Tele-psychiatry intervention; follow-up vs. baseline	Common mental disorders^s^, Alcohol use disorder^t^, Disability and functioning^m^
Liu, 2023 [44]	Yes	RCT - The individual pre-test post-test control group design	Together app nurse-led self-management intervention; follow-up vs. baseline	Depressive symptoms^u^
Öztoprak, 2023 [45]	No	RCT – The individual alternative treatment control group design	Nurse navigation program-based interventions vs. usual care	Quality of life^v^, depressive symptoms^w^, anxiety symptoms^x^
***Digitally delivered treatment with non-specialist involvement***				
Hong, 2023 [46]	Yes	NRCT^d^ – The individual alternative treatment alternative treatment control group design	Nurse-led mHealth intervention vs. usual care	Depressive symptoms^y^
Hanita, 2022 [47]	Yes	Feasibility study - The individual alternative treatment control group design	MyEducation: coronary artery bypass graft application vs. usual care	Depressive symptoms, anxiety symptoms ^z^
Xu, 2021 [48]	Yes	Pilot study - The individual pre-test post-test control group design	The CARE intervention vs. regular community-based rehabilitation	% of drug positive test^aa^, longest period of abstinence^aa^
Rodriguez, 2021 [49]	Yes	RCT^c^- The individual alternative treatment control group design	MIND + intervention vs. MIND intervention without non-specialist support	Depressive symptoms^j^, Anxiety symptoms^k^, Indices of depression^ab^, Indices of anxiety^ab^, Indices of stress^ab^, Mindfulness level^ac^
Anttila, 2019 [33]	Yes	Feasibility study - The cluster multiple treatment and control pre-test design	Intervention 1: The DepisNet-Thai intervention; follow-up vs. baseline	Depressive symptoms^j^, Stress level^ad^
			Intervention 2: The DepisNet-Thai active control; follow-up vs. baseline	Depressive symptoms^j^, Stress level^ad^
Menezes, 2019 [50]	Yes	Pilot study - One-group pre-test post-test design	The CONEMO app intervention; follow-up vs. baseline (1 trial conducted in Brazil, 2 trials conducted in Peru)	Proportion of participants with depression^j^, Proportion of participants with disability^m^, Proportion of participants in 5 quality of life domains^o^
Zhou, 2019 [51]	Yes	RCT^c^ - The individual pre-test post-test control group design	The cyclic adjustment training (CAT) intervention vs. routine nursing care	Level of resilience^ae^
Gonsalves, 2021 [52]	Yes	Pilot study. One-group pre-test post-test design	POD Adventure app intervention; follow-up vs. baseline	Psychosocial problem severity^af^, Mental health symptoms^ag^, Perceived stress^ah^, Mental wellbeing^ai^
Arjadi, 2018 [53]	Yes	RCT^c^ - The individual alternative treatment control group design	GAF-ID intervention vs. online psychoeducation without non-specialist	Depressive symptoms^j^
Araya, 2022 [54]	Yes	RCT^c^ – Brazil: The cluster alternative treatment control group design; Peru: The individual alternative treatment control group design	Digital intervention vs. usual care	Proportion of participants with at least a 50% reduction from baseline depression scores^j^
***Digital supervision of non-specialists***				
Khan, 2019 [55]	Yes	Feasibility study - The cluster alternative treatment control group design	Group management plus intervention vs. enhanced usual care	Psychological distress in terms of states of anxiety and depression^z^, States of only anxiety^z^, States of only depression^z^, Disability and functioning^m^, Psychological profile^aj^, PTSD symptoms^ak^, Generalised distress^j^
Rahman, 2019 [56]	Yes	RCT^c^ –The cluster alternative treatment control group design	Group management plus intervention vs. enhanced usual care	Psychological distress in terms of states of anxiety and depression^z^
Chen, 2022 [57]	Yes	RCT^c^ - The cluster alternative treatment control group design	Chinese Older Adult Collaborations in Health (COACH) vs. enhanced usual care	Depressive symptoms^al^

^a^ The funding sources for each study are listed in the Additional file 10. ^b^Based on schematic diagrams by Campbell et al. [58] ^c^Randomised controlled trial; ^d^Non-randomised controlled trial; The following assessment tools were used: ^e^ENhancing Assessment of Common Therapeutic factors; ^f^Non-standardised questionnaire; ^g^Non-standardised questionnaire; ^h^Self-report at baseline and primary health care physician registries at follow-up; ^i^Self-reporting questionnaire 20-item; ^j^Patient Health Questionnaire-9; ^k^Generalized Anxiety Disorder Questionnaire; ^l^The Shona Symptom Questionnaire; ^m^ WHO Disability Assessment Schedule; ^n o^EuroQoL (Quality of Life)-5 dimensions; ^p^Center for Epidemiologic Studies Depression scale; ^q^Arabic Depression, Anxiety and Stress Scales; ^r^ICEpop CAPability measure for Older people; ^s^Global Health Questionnaire; ^t^Alcohol Use Disorders Identification Test; ^u^Version 2 of Beck Depression Inventory; ^v^Maternal Post-Partum Quality of Life Assessment; ^w^Post-Partum Specific Anxiety Scale; ^x^Edinburgh Postnatal Depression Scale; ^y^Korean Version of the Geriatric Depression Scale -Short form; ^z^Hospital Anxiety and Depression Scale; ^aa^Urine drug screen; ^ab^Depression, Anxiety and Stress Scale; ^ac^The five-facet mindfulness questionnaire; ^ad^Thai version of the 10-item Perceived Stress Scale; ^ae^The Connor-Davidson Resilience Scale; ^af^Youth Top Problems Questionnaire; ^ag^Strengths and Difficulties Questionnaire; ^ah^Perceived Stress Scale; ^ai^Short Warwick-Edinburgh Mental Well-Being Scale; ^aj^Psychological outcome profiles questionnaire; ^ak^Post-traumatic stress symptoms questionnaire; ^al^Hamilton Depression Rating Scale

Among all studies, *n* = 16 studies used various forms of usual procedures for the control condition, such as conventional face-to-face training for non-specialists, and (enhanced) care as usual for service receivers including regular medical care (i.e., pre-and postnatal or cancer care) with additional mental health education, identification of mental illness problems and referral. The remaining studies used the baseline (*n* = 10 studies), waitlist (*n* = 1) and digital intervention without non-specialist involvement (*n* = 1) as the control condition [details on intervention and control conditions in Additional file 6].

Among studies focussing on non-specialists, outcomes were categorized into *competence* to deliver a depression treatment (*n* = 3 studies), and *knowledge* about mental disorders and ways to promote/prevent mental illness (*n* = 1). The latest outcome assessment was 3 months post-baseline. Among studies focussing on service receivers, outcomes of *n* = 21 studies were categorized into severity of mental health problems, comprising different mental illness symptoms or disorders. Six studies focussed on psychosocial functioning outcomes and two studies on MHC use [Additional file 7, table S7.1]. The latest assessment time-points were 12 months post-baseline and 3 months post-intervention for severity of mental health problems and psychosocial outcomes and 1-year post-baseline for MHC use outcomes. None of the studies reported on adverse events (Table 3). In two studies the lost to follow-up rates were above 50%, and in most studies (*n* = 13) the reasons for lost to follow-up were unclear or not mentioned. Only two studies reported that most participants were lost due to intervention-related reasons [Additional file 8].

**Table 3:** Effect sizes

Study, intervention	Outcome	Effect size calculation	Unadjusted effect size^a^	Adjusted effect size^a^	Adjusted variables	Favours intervention vs. control	Favours post intervention vs. baseline
***Digital training of non-specialists; n = 4 studies (6 interventions)***							
Rahman [34]	Competence	Intervention – control (3 months post intervention)	d = 0.16 small			***Intervention***	
Muke, intervention 1 [30]	Competence	Intervention – baseline	d = 0.7 medium				***Post intervention***
Muke, intervention 2 [30]		Intervention – baseline	d = 0.32 medium				***Post intervention***
Nisar [35]	Competence	Intervention – control (3 months post intervention)	d = 0.1 small	d = 0.1 small	Sociodemographics, prior mental health training	Control	
Pereira, intervention 1 [31]	Knowledge	Intervention – baseline		d = 0.1 small	Clusters (Schools)		***Post intervention***
Pereira, intervention 2 [31]				d = 0.08 small	Clusters (Schools)		Baseline
***Digital support for non-specialists; n = 11 studies (12 interventions)***							
Maulik [36]	Mental healthcare use	Intervention – baseline	d = 0.68 medium				***Post intervention***
Maulik [37]	Mental healthcare use	Intervention – baseline	d = 1.17 large	d = 1.18, large	Clusters (villages), sociodemographics		***Post intervention***
Doukani [38]	Severity of mental health problems	Intervention – baseline	d = 0.83–1 large (3 outcomes)				***Post intervention***
Dambi, intervention 1 [32]	Severity of mental health problems	Intervention – baseline	d = 0.6–0.7.1 medium (3 outcomes), d = 1.08 large				***Post intervention***
	Psychosocial functioning		d = 0.39 medium				***Post intervention***
Dambi, intervention 2 [32]	Severity of mental health problems	Intervention – baseline	d = 0.88–1.35 large (4 outcomes)				***Post intervention***
	Psychosocial functioning		d = 0.06 small				***Post intervention***
Chibanda [39]	Severity of mental health problems	Intervention – control (6 months post baseline)	d = 1.07 large	d = 1.04, large	Sociodemographics, (mental) health status		***Post intervention***
Ross [40]	Severity of mental health problems	Intervention – control (1 month post baseline)	d = 0.27 medium			Control	
		Intervention – control (2 months post baseline)	d = 0.24 medium			***Intervention***	
Ebrahem [41]	Severity of mental health problems	Intervention – baseline	d = 0.28–0.29 medium (3 outcomes)				***Post intervention***
Scazufca [42]	Severity of mental health problems	Intervention – control (1 month post intervention)	d = 1.49 large			***Intervention***	
	Psychosocial functioning		d = 0.06 small			Control	
			d = 0.17 small			***Intervention***	
Garg [43]	Severity of mental health problems	Intervention – baseline (3 months post baseline)	d = 0.69 medium-d = 1.13–3.22 large (2 outcomes)				***Post intervention***
Liu [44]	Severity of mental health problems	(Intervention – baseline) – (control – baseline)	d = 0.18 small			***Intervention***	
		(Intervention – baseline) – (control – baseline) (12 weeks post intervention)	d = 0.54 medium			***Intervention***	
Öztoprak [45]	Psychosocial functioning	Intervention – control (10 weeks post baseline)	d = 2.24 large			***Intervention***	
		Intervention – control (16 weeks post baseline)	d = 3.39 large			***Intervention***	
	Severity of mental health problems	Intervention – control (4 weeks post baseline)	d = 2.91 large			***Intervention***	
		Intervention – control (5 weeks post baseline)	d = 1.14–2.5 large (2 outcomes)			***Intervention***	
		Intervention – control (10 weeks post baseline)	d = 1.79–3.17 large (2 outcomes)			***Intervention***	
		Intervention – control (16 weeks after baseline)	d = 1.86–3.28 large (2 outcomes)			***Intervention***	
***Digitally delivered treatment with non-specialist involvement; n = 10 studies (11 interventions)***							
Hong [46]	Severity of mental health problems	(Intervention – baseline) – (control – baseline)		d = 0.02 small	Baseline mental health	***Intervention***	
Hanita [47]	Severity of mental health problems	Intervention – control outcome (1 month post intervention)	d = 0.2 medium, d = 1.35 large			***Intervention***	
Xu [48]	Severity of mental health problems	Intervention – control	d = 0.62–0.66, medium (2 outcomes)			***Intervention***	
Rodriguez [49]	Severity of mental health problems	(Intervention – baseline) – (control – baseline)	d = 0.07–0.1 small (4 outcomes), d = 0.33 medium			***Intervention***	
	Psychosocial functioning		d = 0.19 small			Control	
Anttila, intervention 1 [33]	Severity of mental health problems	Intervention – control (1 month post intervention)	d = 0.07 small				***Post Intervention***
	Severity of mental health problems		d = 0.14 small				Baseline
Anttila, intervention 2 [33]	Severity of mental health problems	Intervention – control (1 month post intervention)	d = 0.09 small, d = 0.27 medium				***Post intervention***
Menezes [50]	Severity of mental health problems	Proportion of outcome at follow-up	In all trials: recovered from depression *n* > 50%, with disability n ≤ 10%; Brazil trial: without suicide risk *n* = 100%, Peru trials: *n* = 0%				***Post intervention***
Zhou [51]	Psychosocial functioning	Intervention – control		d = 2.68 large	Baseline mental health	***Intervention***	
Gonsalves [52]	Severity of mental health problems	Intervention – baseline (2–3 weeks post intervention)	d = 0.45–0.51 medium (2 outcomes) - d = 1.75 large				***Post intervention***
		Intervention – baseline (9–10 weeks post intervention)	d = 0.6 medium (2 outcomes) - d = 1.94 large				***Post intervention***
	Psychosocial functioning	Intervention – baseline (2–3 weeks post intervention)	d = 0.07 small				***Post intervention***
		Intervention – baseline (9–10 weeks post intervention)	d = 0.32 medium				***Post intervention***
Arjadi [53]	Severity of mental health problems	Intervention – control outcome (2 weeks post intervention)		d = 0.39 medium	Sociodemographics, baseline mental health	***Intervention***	
Araya [54]	Severity of mental health problems	Intervention – control (3 months post intervention)		d = 0.11–0.18 small (2 outcomes)	Brazil study: cluster (residency), Peru: health centre, both: Baseline mental health	***Intervention***	
***Digitally supervised non-specialists; n = 3 studies (3 interventions)***							
Khan [55]	Severity of mental health problems	Intervention – control (7 weeks post baseline)		d = 0.15 small (2 outcomes), d = 0.53–0.62 medium (4 outcomes), d = 0.86 large	Baseline mental health	***Intervention***	
Rahman [56]	Severity of mental health problems	Intervention – control (1 week post intervention)		d = 0.79 medium	Baseline mental health	***Intervention***	
		Intervention – control (3 months post intervention)		d = 0.6 medium		Control	
Chen [57]	Severity of mental health problems	Intervention – control (3 months post baseline)	d = 0.45 medium	d = 0.13 small	Sociodemographics, baseline (mental) health	***Intervention***	
		Intervention – control (6 months post baseline)	d = 0.82 large	d = 0.25 medium		***Intervention***	
		Intervention – control (9 months post baseline)	d = 1.09 large	d = 0.34 medium		***Intervention***	
		Intervention – control (12 months post baseline)	d = 1.37 large	d = 0.4 medium		***Intervention***	

^a^The interpretation are based on following rules: small (d < 0.2), medium (d = 0.2–0.8), large (d > 0.8) [29]

### Participant characteristics

#### Non-specialists

The mean age of non-specialist participants ranged from 20 to 40 years, and across studies, more than 70% of participants were female. Lady health workers, Accredited Social Health Activist (ASHAs), ASHA facilitators, community/multipurpose health workers or volunteers, nursing students and teachers were selected as non-specialists who received digital training/education (Table 1).

#### Service receivers

Service receivers were aged between 18–65 years in *n* = 19 studies, 15–16 years in two studies, and 65+ in three studies. More than 50% of the intervention receivers were female in most studies (*n* = 22). Additionally, most studies (*n* = 17) differentiated between two genders (men, women), seven studies considered biological sex, and in four studies, the terms “gender” and “sex” were used interchangeably (Table 1).

In *n* = 11 studies participants reported symptoms of common mental disorders (CMD), including depressive, anxiety and unexplained somatic symptoms with chronic somatic comorbidity (diabetes, hypertension), which were assessed using standardized clinical assessment tools, such as the Patient Health Questionnaire-9. In two studies participants self-reported having depressive or general psychological distress symptoms. In three studies participants had a probable mental disorder, including alcohol use, substance use or major depressive disorder based on structured clinical interviews using the Statistical Manual of Mental Disorders – 5 or clinical judgements. Participants from other studies had somatic diseases, such as breast cancer, HIV, coronary heart disease or spinal cord injury (*n* = 4). In three studies participants were considered healthy (women at postnatal stage, school children, parents) (Table 1). Furthermore, *n* = 14 studies excluded participants with severe mental disorders, including high suicide risks or psychotic disorders [Additional file 9].

### Heterogeneity assessment

The included studies were very heterogeneous regarding the PICO characteristics. First, studies focussed either on the non-specialist or the service receivers (Table 1). Second, the age range and type of (mental) health status differed among the service receivers (Table 1). Third, the types of intervention and control conditions differed regarding the use of digital technologies, the tasks of the non-specialists and the type of care [Additional file 6]. Fourth, some studies focus on the difference of the (change in) outcome between two groups at follow-up, while others assessed the change of the outcome from baseline to follow-up. Fifth, it was difficult to determine whether the drop-outs were due to the side effects related to the mental health intervention or other reasons [Additional file 8]. Due to all these reasons, a planned meta-analysis was not deemed feasible.

#### RQ 1: how are non-specialists and digital technologies combined in mental health interventions?

**Fig. 2:**
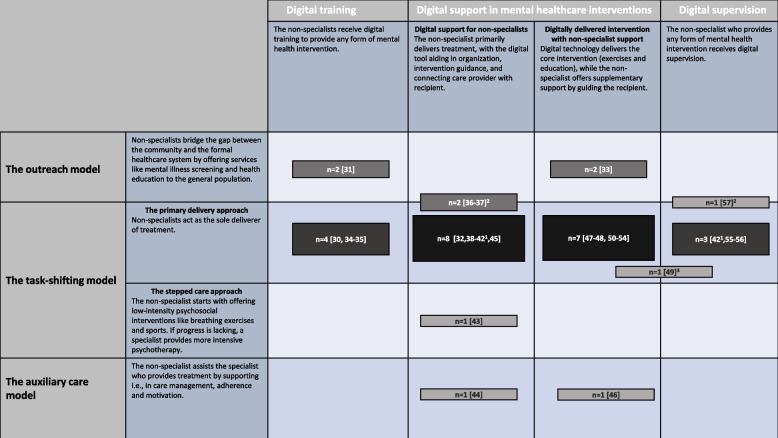
Conceptual framework of mental health interventions combining digital technologies and non-specialist mental health workers. Notes: This model allocated each intervention across a matrix combining non-specialist models based on the framework proposed by Barnett et al. [7] and the m-health function model by Agarwal et al. [19]. The numbers of interventions (n) are presented in each category. ^1^ One study [42] was categorized twice, because the digital component was used to support in the delivery of MHC and for supervision of the non-specialist. ^2^These intervention comprise two non-specialist models. ^3^This intervention uses the digital technology to support in the delivery of MHC and for supervision of the non-specialist

Digital technology was identified to adopt four different purposes in four non-specialist models (Fig. 2). The purposes of digital technology were: 1. Digital training for non-specialists, 2. Supporting non-specialist-delivered interventions, 3. Digitally delivered intervention with non-specialist involvement, and 4. Digital supervision of non-specialists. Digital technologies were mostly used in task-shifting care models, particularly in primary-deliverer care approaches (*n* = 24 intervention) followed by stepped-care approaches (*n* = 1). Additionally, technology was used in non-specialist outreach models (*n* = 7) and auxiliary care models (*n* = 2). A detailed rationale for the categorization of interventions can be found in the Additional file 10.

#### Digital training of non-specialists

In four studies, non-specialists were trained through mobile-based or tablet-based applications or websites with remote or face-to-face support from their trainers to provide depression treatment or mental health promotion strategies [Additional file 10, table S10.1]. All training or education interventions lasted from a minimum of 9 hours to a maximum of 5 days [Additional file 6].

#### Digitally supporting non-specialist-delivered interventions

In *n* = 11 studies, digital technology supported non-specialists in delivering treatment through data collection, decision support, setting alerts and reminders, enabling emergency contact, a visualization tool for mental health educational purposes and as a communication tool [Additional file 10, table S10.1].

In five studies, the non-specialist provided treatment synchronously or asynchronously with digital support. Synchronous treatment delivery refers to when the service receiver and the non-specialist communicate simultaneously (i.e., by phone). Asynchronous contact refers to a certain time delay between the response (i.e., chat or email conversations) [59]. In the remaining studies, treatment was delivered face-to-face (*n* = 4), or using a hybrid method (*n* = 2). The following treatment types were generally provided in (bi-) weekly sessions within a time frame ranging from one to 4 months: problem-solving treatment (*n* = 3), treatment of CMD based on mhGAP guidelines (*n* = 2), psychosocial and emotional support (*n* = 4), behavioural activation treatment (*n* = 1) by non-specialist. In one study the non-specialist supported the specialist (auxiliary care model), who provided treatment, by providing basic emotional counselling and involving family members. In two studies, non-specialists were additionally used for promotional activities, including providing an anti-stigma campaign and screening of mental health problems [Additional file 6].

#### Digitally delivered intervention with non-specialist involvement

In ten studies, digital technologies in the form of mobile or tablet-based applications were mainly used to provide specific intervention components (mental-health information and exercises), while the non-specialist was available for further support (face-to-face, remotely or both) [Additional file 10, table S10.1]. The non-specialists were mainly responsible for introducing and implementing the digital intervention, promoting adherence and maintaining the motivation of service receivers, resolving technical issues and reinforcing digitally delivered treatment content. In one study, mental health promotion techniques were digitally provided, while in nine studies, mental health treatment and prevention services were provided. The following treatments were delivered: Behavioural activation therapy (*n* = 3 studies), problem-solving therapy (*n* = 1), strategies for drug rehabilitation (*n* = 1), cyclic adjustment training (*n* = 1), mindfulness therapy (*n* = 1), general psychosocial treatment (education, symptom assessment and self-management skills) (*n* = 1). The duration of the interventions ranged from 2 weeks to 6 months while the treatment sessions took place at least once a week [Additional file 6].

#### Digital supervision of non-specialists

Among the five studies focussing on the service receiver, digital technologies were used to supervise the non-specialists’ work through online meetings, phone calls, and audio-recording sessions. Three of these studies used digital technology solely for (weekly or monthly) supervision purposes [Additional file 10, Table S10.1; Additional file 6].

### Effectiveness of using digital technologies in non-specialist interventions

Table 3 shows the effect sizes for following outcome categories: non-specialists’ competence and knowledge, service receivers’ MHC use, severity of mental health problems, and psychosocial functioning. The detailed effect sizes for each outcome can be found elsewhere [Additional file 7, Table S.7.2]. The effect sizes were either unadjusted or adjusted for various potential confounders. Relative to unadjusted effect sizes, the adjustment for confounders did not change the interpretation of the effect sizes according to three studies for which both the unadjusted and adjusted outcomes were reported. Only in one study four adjusted outcomes provided a conservative effect estimate compared to the unadjusted outcome (Table 3).

#### RQ 2: Are digital training interventions effective for non-specialists?

Digital training or education, with a minimum of 9 hours, generally seems beneficial for non-specialists. Three out of four digital training/education interventions showed small to medium improvements from baseline to post-intervention (d = 0.1–0.7 in 3 out of 4 outcomes) in non-specialists’ competencies in delivering treatment and knowledge to promote mental health. In one out of two cases non-specialist’s competencies were better in the intervention group compared to usual face-to-face training with a small effect size (d = 0.16 in 1 out of 2 outcomes).

#### RQ 3: Are mental health interventions delivered by non-specialists who are supported by digital technologies effective for the service receivers?

Non-specialist-delivered interventions that use digital technology for various functions (data collection, decision support, visualisation, communication, alerts and reminders and care coordination) were generally beneficial for service receivers. This is because around 95% of the outcomes across the *n* = 11 studies favoured the intervention in contrast to different control conditions, with mostly large effect sizes (large: d > = 0.8 in 24 out of 38 outcomes). In particular, two interventions revealed an increased MHC use (after screening), while seven interventions showed a decrease in severity of mental health problems and increase in psychosocial function (after treatment) relative to the baseline values. In addition, three interventions showed that such treatment interventions were more effective in decreasing severity of mental health problems and increasing psychosocial outcomes than regular care without any mental health treatment and enhanced usual care, including brief counselling, mental health education and/or referral to specialists (Table 3) [Additional file 7, Table S.7.2].

#### RQ 4: Are digitally delivered interventions with additional non-specialist involvement effective for service receivers?

Digitally delivered interventions with additional non-specialist involvement were beneficial for the service receivers with mostly small effect sizes (d = 0.11–0.2 in 12 out of 25 outcomes). More than 90% of outcomes across the 10 studies favoured digitally delivered interventions with non-specialist involvement compared to different controls. Specifically, these interventions reduced the severity of mental health problems and increased the psychosocial functioning of service receivers relative to baseline (*n* = 4 interventions), (enhanced) regular care including brief counselling or no mental health care (*n* = 5), and digitally delivered interventions without non-specialist involvement (*n* = 2) (Table 3) [Additional file 7, Table S.7.2].

#### RQ 5: Are digital supervision tools effective for non-specialists?

Despite none of the included studies investigating the direct effect of digital supervision for non-specialists (i.e., competence level), the evidence indirectly suggests that such interventions benefit the non-specialists. Three non-specialist-delivered interventions that use digital technology solely for supervision purposes showed lower severity of mental illness problems with mostly medium effect sizes (d = 0.2–0.8 in 10 out of 17 outcomes) at follow-up in contrast to enhanced care as usual. However, because the usual care control group does not include non-specialists, it remains unclear how effective digital supervision is relative to no or usual on-site supervision (Table 3) [Additional file 7, Table S.7.2].

### Study quality and potential bias

The overall certainty of the evidence at hand can be judged as being mostly low due to most (N)RCTs having a moderate to high risk of bias (*n* = 15 studies) [Additional file 11], the high heterogeneity of the study and intervention characteristics (Table 1), and potential publication bias due to most studies being conducted in Asian and South-American settings [Additional file 12].

## Discussion

This systematic review investigated how different non-specialist models were combined with digital technology support models and whether these combinations can effectively reduce the MHC gap in LMICs. Digital technology was predominantly used in task-shifting care interventions, with the focus on the primary-deliverer care approaches, for purposes such as training, supervising, and supporting the non-specialist in treatment delivery and delivering treatment components with non-specialist involvement. Treatment interventions combining non-specialists and digital technology mainly focused on people with non-severe CMD or subthreshold symptoms. This review shows that any digital training improved the competencies and knowledge of non-specialists with a small to medium effect size (d ≤ 0.8). Furthermore, non-specialist-delivered interventions using digital technology as further support for the service-delivery and supervision of the non-specialist work improved mental health outcomes in service receivers with overall medium to large effect sizes (d ≥ 0.2). Similarly, digitally delivered interventions with additional non-specialist involvement improved the service receiver’s mental health outcomes with mostly medium effect sizes (d = 0.2–0.8). However, the overall certainty of the evidence at hand was evaluated to be low.

### Digital technology in non-specialist mental health models

Interestingly, digital technology was primarily utilised in interventions where non-specialists were the primary service deliverers [7]. Unsurprisingly, digital technology was not often used in auxiliary care models or task-shifting stepped-care approaches. Reasons for the lack of such collaborative care methods are the general shortages of specialists and specific regulations that probably favour the primary-delivery model [13, 60].

The purposes for and functions of digital technologies identified in this review were also identified in interventions targeting other health domains, such as for example communicable diseases [19]. However, our review indicates that, specifically in mental health interventions, digital technology was used to deliver main treatment components, such as mental health education and evidence-based exercises, with additional non-specialist involvement.

### Digital training and supervision

We can cautiously assume that digital training appears to be as effective as face-to-face training in increasing the non-specialist’s competence in providing MHC treatment. Additionally, the available evidence suggests that non-specialists who are digitally supervised can effectively improve mental health outcomes in service receivers. Other researchers have supported these findings by outlining the benefits associated with digital training and supervision, including reducing the amount of training and overcoming structural barriers, such as inviting on-site specialists for training and supervision [19]. Furthermore, according to a study examining the acceptability and feasibility of digital training, non-specialists perceived using digital technologies as useful and convenient, even given a lack of acquaintance with technology [61].

Despite the general benefits of digital training and supervision, more robust evidence is needed to quantify the long-term effects of digital training compared to face-to-face training. Moreover, current evidence does not provide insights into the benefits of using digital vs usual supervision methods for non-specialists. These results align with the current literature on the digital supervision of general frontline health workers, suggesting that evidence seems either lacking or inconclusive [19]. In the future, studies are needed that primarily examine if digital supervision helps to maintain the competencies of the non-specialists sustainably, compared to on-site supervision or no supervision in the context of LMICs. Results from such studies could facilitate more equitable MHC provision globally by enabling, for example, specialists from resource-rich settings to supervise non-specialists from resource-poor settings virtually.

### Mental health treatment involving non-specialists and digital technology

Despite the limited certainty of the evidence, the circumstance that most outcomes favoured the intervention groups, suggest that incorporating digital technology into non-specialist MHC interventions can be beneficial. This is supported by previous research demonstrating the practical benefits of using technology, such as time-saving for care providers that enable them to engage in other income-generating activities, as seen in two qualitative studies on midwife care in Indonesia [62, 63]. Another qualitative study found that non-specialist MHC workers prefer using digital protocols for their convenience during the treatment when adequately trained on using the digital device [64]. Other studies have shown that digital reminder messages can improve the non-specialist’s management of malaria in children by 24%, and digital decision support tools can increase non-specialist’s adherence to treatment protocols for early childhood disorders [65, 66]. These findings could be applied to non-specialists involved in mental health treatment.

However, in spite of the general benefits of using digital technology in non-specialist treatment models, gaps remain in current literature, such as the lack of insights into the clinical influences of specific digital functions. For example, in some cases, non-specialist and service receiver communication took place face-to-face, while in other cases, remote synchronous or asynchronous communication was used. Previous research suggests that specific components of in-person therapy, such as non-verbal communication, can contribute to psychopathological improvements [67]. Because these components are usually missing in remote communication and the conversation dynamics may differ between asynchronous and synchronous conversations, it remains to be seen if and what type of remote communication for primary-delivery models is most effective. Additionally, in digitally delivered interventions with asynchronous communication, there may be a certain time period between the potential exposure to stress and the contact with the non-specialist. In usual on-site psychotherapy, the generation of a safe space enables the mediation and modulation of stress, which is a key component for treatment success [67]. Whether or not this lack of safe space may be ignorable or even harmful still needs to be clarified because none of the included studies examined adverse effects.

Moreover, the current body of evidence does not provide any insights into what effect size (d) can be considered clinically meaningful. Clinical meaningfulness can be defined by considering the minimal important difference (MID), which refers to the smallest change in the outcome score of interest that is perceived as beneficial for the patients and would warrant a change in the management of the health problem in the absence of adverse effects and high costs [68, 69]. Considering what effect size constitutes a MID highly depends on the population, context and the measurement tool [68, 70]. Because of the high heterogeneity of the studies regarding the PICO characteristics and the utilised measurement tools, comparisons of the different effect sizes should be conducted with caution. However, although the extent of clinical meaningfulness remains unclear, we can assume that interventions combining digital technologies and non-specialists are helpful for people with non-severe CMD or subthreshold symptoms by at least maintaining their mental health and potentially by preventing the progression to a full-blown disorder. To gain more robust evidence on this assumption, long-term outcomes and potential adverse effects should be the focus in future studies. Apart from that, most of the studies excluded participants with severe mental health problems, such as suicidality or psychotic disorders [Additional file 9], who may require medication and close monitoring. Hence, we can assume that non-specialist interventions with digital technologies do not replace specialised mental health workers but rather act as a substitute for population groups that do not have any other alternative to receive support.

### Health equity viewpoint

Given that such interventions may be a potential solution especially for resource poor areas, where no alternative mental health support may be available, it still remains unclear how such interventions can be implemented in such settings, because most studies were conducted in urban areas. Although access to digital technology increased rapidly in LMICs, 33% of adults in rural areas are less likely to use mobile internet than those in urban areas [18]. Structural implications, such as power cuts, lack of network coverage, and increased internet costs may explain this digital divide [18]. Hence, future research needs to identify ways to overcome these structural barriers, such as, for example, through applications that do not require an internet connection. Moreover, there is a need to tailor these interventions to men in LMICs, as most of the included participants were women. Current literature indicates that especially men show consistently fewer positive attitudes toward MHC use as compared to females [3].

## Strengths and limitations

Due to the large heterogeneity of included studies, a meta-analysis could not be performed, and the effects-sizes must be compared with caution. Additionally, the calculation of Cohen’s d did not fulfil the underlying assumptions for *n* = 12 outcomes. However, a sensitivity analysis comparing Cohen’s d with the statistical corrections of Hedge’s g and Glass delta [71], showed no differences in the interpretation of most of the effect sizes [Additional file 13]. Moreover, a fixed correlation coefficient of r = 0.5 was assumed for *n* = 22 outcomes. The sensitivity analysis results showed that among these outcomes, *n* = 18 outcomes showed no difference in the interpretation of the effect size when using a regression coefficient of r = 0.5, r = 0.2 or r = 0.8 [Additional file 14]. Finally, only one author conducted the GRADE assessment. However, no major concerns regarding interrater reliability were assumed, given that the bias assessment, which was one major component of the GRADE guidelines, was conducted by two authors.

Nevertheless, this systematic review generated a detailed overview of the existing literature by including a broad range of interventions, among which mental health promotion, prevention, and treatment strategies. Additionally, evidence gaps and promising intervention approaches could be identified, and the existing frameworks on non-specialist models and digital technology for health interventions could be combined and thus expanded. Finally, this review is of high methodological quality according to the AMSTAR 2 checklist [Additional file 2].

## Conclusion

This systematic review of *n* = 28 studies shows that digital technologies in non-specialist mental health interventions tended to have a positive impact in the four outcome categories: 1) competencies and knowledge of non-specialists, 2) severity of mental health problems 3) MHC use and 4) psychosocial functioning of service receivers. Digital technology was mostly used in task-shifting primary-deliverer care models. In some cases, technology was also used in task-shifting stepped care, outreach and auxiliary care models. Most treatment interventions involving non-specialists and digital technology addressed people with non-severe CMD and subthreshold symptoms. Digital technology adopted four purposes: to train and supervise non-specialists, to support non-specialists in the delivery of treatment, and to provide digital treatment with non-specialist involvement. The available results show that using digital technology for all four purposes in different non-specialist interventions can be effective for non-specialists and for service receivers, especially when no other adequate care can be provided. However, the certainty of the current evidence is poor. Hence, several gaps in the current body of evidence were identified that need to be addressed in future studies in the following ways: 1. generating more rigorous study methodologies with low risk of bias, 2. generating more robust evidence to better understand and compare the magnitude of the effectiveness and clinical relevance of the different interventions, 3. generating insights into the clinical influences of the specific digital functions, 4. building evidence on the effectiveness of digital supervision compared to on-site or no supervision, 5. studying potential harms and the long-term effects of such interventions, 6. expanding and tailoring such interventions to men and marginalized communities to address global health equity. Given that this review unveils the general potential of combining digital technology with non-specialists in mental health interventions, addressing the current knowledge gap can be one approach to successfully reduce the global MHC gap, especially in resource-poor settings.

### Supplementary Information

**Additional file 1.**

**Additional file 2.**

**Additional file 3.**

**Additional file 4.**

**Additional file 5.**

**Additional file 6.**

**Additional file 7.**

**Additional file 8.**

**Additional file 9.**

**Additional file 10.**

**Additional file 11.**

**Additional file 12.**

**Additional file 13.**

**Additional file 14.**

**Additional file 15.**

## Data Availability

The search syntax for PubMed can be found in Additional file 3. The complete search syntax and the data extraction sheet used for analysis in this article is available upon request from the corresponding author on reasonable request.

## Abbreviations

AMSTAR: A measurement tool to assess systematic reviews
ASHA: Accredited social health workers
CCA: Complete case analysis
CINHAL: Cumulative index to nursing and allied health literature
CMD: Common mental disorders
CTRI: Clinical trial registry, India
GRADE: Grading of Recommendations, Assessment, Development and Evaluation
ICTRP: International clinical trial register platform
ISRCTN: International standard randomised controlled trial
ITT: Intention-to-treat
LMIC: Low-and middle-income countries
MHC: Mental health care
MID: Minimal important difference
NRCT: Non-randomized-controlled trial
PRISMA: Preferred reporting items for systematic reviews and meta-analysis
QOL: Quality of life
RCT: Randomized-controlled trial
ROB: Risk of Bias
RQ: Research question
SWiM: Synthesis without Meta-analysis
