# Supplementary material for: The effectiveness of mental health interventions involving non-specialists and digital technology in low-and middle-income countries – a systematic review
Source: BMC Public Health. 2024 Jan 3;24:77. doi: 10.1186/s12889-023-17417-6 (PMC10763181; doi:10.1186/s12889-023-17417-6)
Supplement: Supplementary file 2 — Additional file 2. [file 12889_2023_17417_MOESM2_ESM.docx]

# **ADDITIONAL FILE 2: AMSTAR 2 CHECKLIST**

Overall outcome of AMSTAR2 assessment: this review is of high quality because it has no critical weaknesses (Table S2).

**Table S2. Amstar 2 checklist**

| **AMSTAR2 Item** | **Score** |
| --- | --- |
| 1. Did the research questions and inclusion criteria for the review include the components of PICO? | **YES**, see methods, eligibility criteria |
| 2^a^. Did the report of the review contain an explicit statement that the review methods were established prior to the conduct of the review and did the report justify any significant deviations from the protocol? | **YES**, review protocol on PROSPERO: CRD42021293016 |
| 3. Did the review authors explain their selection of the study designs for inclusion in the review? | **YES**, randomised-controlled trials, non-randomised controlled trials, pilot and feasibility studies were included (see methods, eligibility criteria) |
| 4^a^. Did the review authors use a comprehensive literature search strategy? | **YES**, 4 databases (Pubmed, Psychological Information Database (PsychINFO), Cumulative Index to Nursing and Allied Health Literature (CINAHL), Web of Science), 3 trial registries (International Standard Randomised Controlled Trial Number (ISRCTN), International Clinical Trials Registry Platform (ICTRP) by the WHO, and Clinical Trials Registry- India (CTRI), and forward and backward citation of included studies, as well as protocols that were identified in the title-and abstract screening in the database and trial registry search. See methods, search strategy and study selection. |
| 5. Did the review authors perform study selection in duplicate? | **YES**, stated in Methods, study selection |
| 6. Did the review authors perform data extraction in duplicate? | **YES**, stated in Methods, data extraction |
| 7^a^. Did the review authors provide a list of excluded studies and justify the exclusions? | **YES**, a list of justification of excluded articles of the full-text screening is provided in table S4 (additional file 4) |
| 8. Did the review authors describe the included studies in adequate detail? | **YES**, table 1, table 2 and additional file 6 |
| 9^a^. Did the review authors use a satisfactory technique for assessing the risk of bias (RoB) in individual studies that were included in the review? | **YES**, RoB2 (Version 2 of the Cochrane risk-of-bias tool for individual and cluster RCTs (1) and ROBINS-I (Risk Of Bias In NRCTs of Interventions) (ROBINS-I) (2); see methods, study quality and additional file 11 |
| 10. Did the review authors report on the sources of funding for the studies included in the review? | **YES**, Additional file 15 |
| 11^a^. If meta-analysis was performed did the review authors use appropriate methods for statistical combination of results? | No meta-analysis was performed |
| 12. If meta-analysis was performed, did the review authors assess the potential impact of RoB in individual studies on the results of the meta-analysis or other evidence synthesis? | No meta-analysis was performed |
| 13^a^. Did the review authors account for RoB in individual studies when interpreting/discussing the results of the review? | **YES**, stated in Study quality and potential bias, discussion and conclusion |
| 14. Did the review authors provide a satisfactory explanation for, and discussion of, any heterogeneity observed in the results of the review? | **YES**, stated in heterogeneity assessment |
| 15^a^. If they performed quantitative synthesis did the review authors carry out an adequate investigation of publication bias (small study bias) and discuss its likely impact on the results of the review? | No meta-analysis was performed |
| 16. Did the review authors report any potential sources of conflict of interest, including any funding they received for conducting the review? | **YES**, no conflict reported (Declarations) |

Note. This table is based on the AMSTAR checklist (3). AMSTAR2 can be used as a checklist for conducting and self-assessing the quality of own systematic reviews. It consists of a standardised scale with 16 items that can be scored as YES, Partial YES or NO. The 16 items consist of 7 critical items (depicted with ^a^) and 9 non-critical items. Following criteria is used to measure the overall quality of a systematic review:

- High: 0 critical weaknesses or 1 non-critical weakness,
- Moderate: 0 critical weaknesses or 1< non-critical weakness,
- Low: 1 critical weakness with or without non-critical weaknesses,
- Critically-low: 1< critical weakness with or without non-critical weaknesses

Abbreviation: AMSTAR2, A Measurement Tool to Assess Systematic Reviews, version 2.

References:

1. Sterne J, Savović J, Page M, et al. RoB 2: a revised tool for assessing risk of bias in randomised trials. BMJ. 2019;366:(14898). doi: 10.1136/bmj.l4898.

2. Sterne JA, Hernán MA, Reeves BC, et al. ROBINS-I: A tool for assessing risk of bias in non-randomised studies of interventions. BMJ. 2016;355(i4919). doi: 10.1136/bmj.i4919.

3. Shea BJ, Reeves BC, Wells G, et al. AMSTAR 2: A critical appraisal tool for systematic reviews that include randomised or non-randomised studies of healthcare interventions, or both. BMJ. 2017;358 (j4008):1–9. doi: https://doi.org/10.1136/bmj.j4008.
