# Supplementary material for: The effectiveness of mental health interventions involving non-specialists and digital technology in low-and middle-income countries – a systematic review
Source: BMC Public Health. 2024 Jan 3;24:77. doi: 10.1186/s12889-023-17417-6 (PMC10763181; doi:10.1186/s12889-023-17417-6)
Supplement: Supplementary file 3 — Additional file 3. [file 12889_2023_17417_MOESM3_ESM.docx]

# **ADDITIONAL FILE 3: SEARCH SYNTAX EXAMPLE**

**Table S3. Search syntax for PubMed**

**First search conducted from inception until 01.12.2021**

**Updated second search conducted from 02.12.2021-18.09.2023**

| **Block 1: LMICs** | *Rationale:*  *we added the countries mentioned as lower or middle income countries in the world bank and added synonyms for some countries based on the authors Ginneken et al.* (1) *+ Cochrane LMIC filters* (2) |
| --- | --- |
|  | Syntax |
| #1 specific LMICs | (((((((((((((((((((((((((((((((((((((((((((((((((((((((((((((((((((((((((((((((((((((((((((((((((((((((((((((((((((((((((((((((((((((((((((((((((((((((((((((((((((((((((((((((Afghanistan[Title/Abstract]) OR (Angola[Title/Abstract])) OR (Albania[Title/Abstract])) OR (Algeria[Title/Abstract])) OR (Argentina[Title/Abstract])) OR (Armenia[Title/Abstract])) OR (American Samoa[Title/Abstract])) OR (Azerbaijan[Title/Abstract])) OR (Burundi[Title/Abstract])) OR (Benin[Title/Abstract])) OR ("Burkina Faso"[Title/Abstract])) OR ("Burkina Fasso"[Title/Abstract])) OR ("Upper Volta"[Title/Abstract])) OR (Bangladesh[Title/Abstract])) OR (Bulgaria[Title/Abstract])) OR ("Bosnia and Herzegovina"[Title/Abstract])) OR (Hercegovina[Title/Abstract])) OR (Belarus[Title/Abstract])) OR (Byelarus[Title/Abstract])) OR (Byelorussian[Title/Abstract])) OR (Belize[Title/Abstract])) OR (Bolivia[Title/Abstract])) OR (Brazil[Title/Abstract])) OR (Brasil[Title/Abstract])) OR (Botswana[Title/Abstract])) OR ("Central African Republic"[Title/Abstract])) OR (China[Title/Abstract])) OR ("Côte d'Ivoire"[Title/Abstract])) OR (Cameroon[Title/Abstract])) OR (Cameroons[Title/Abstract])) OR (Cameron[Title/Abstract])) OR (Congo[Title/Abstract])) OR (Colombia[Title/Abstract])) OR (Comoros[Title/Abstract])) OR ("Comoro Islands"[Title/Abstract])) OR (Comores[Title/Abstract])) OR ("Cabo Verde"[Title/Abstract])) OR (Verde[Title/Abstract])) OR ("Costa Rica"[Title/Abstract])) OR (Cuba[Title/Abstract])) OR (Dominica[Title/Abstract])) OR ("Dominican Republic"[Title/Abstract])) OR (Ecuador[Title/Abstract])) OR (Egypt[Title/Abstract])) OR (Eritrea[Title/Abstract])) OR (Ethiopia[Title/Abstract])) OR (Fiji[Title/Abstract])) OR (Micronesia[Title/Abstract])) OR (Gabon[Title/Abstract])) OR (Georgia[Title/Abstract])) OR (Ghana[Title/Abstract])) OR (Guinea[Title/Abstract])) OR (Gambia[Title/Abstract])) OR (Guinea-Bissau[Title/Abstract])) OR ("Equatorial Guinea"[Title/Abstract])) OR (Grenada[Title/Abstract])) OR (Guatemala[Title/Abstract])) OR (Guiana[Title/Abstract])) OR (Guyana[Title/Abstract])) OR (Honduras[Title/Abstract])) OR (Haiti[Title/Abstract])) OR (Indonesia[Title/Abstract])) OR (India[Title/Abstract])) OR (Iran[Title/Abstract])) OR (Iraq[Title/Abstract])) OR (Jamaica[Title/Abstract])) OR (Kazakhstan[Title/Abstract])) OR (Kazakh[Title/Abstract])) OR (Jordan[Title/Abstract])) OR (Kenya[Title/Abstract])) OR (Kyrgyzstan[Title/Abstract])) OR (Kirghizia[Title/Abstract])) OR (Kyrgyz[Title/Abstract])) ) OR (Kirgizstan[Title/Abstract])) OR (Cambodia[Title/Abstract])) OR (Kiribati[Title/Abstract])) OR ("Lao PDR"[Title/Abstract])) OR (Lebanon[Title/Abstract])) OR (Liberia[Title/Abstract])) OR (Libya[Title/Abstract])) OR ("St. Lucia"[Title/Abstract])) OR ("Saint Lucia"[Title/Abstract])) OR ("Sri Lanka"[Title/Abstract])) OR (Ceylon[Title/Abstract])) OR (Lesotho[Title/Abstract])) OR (Basutoland[Title/Abstract])) OR (Morocco[Title/Abstract])) OR (Moldova[Title/Abstract])) OR (Madagascar[Title/Abstract])) OR ("Malagasy Republic"[Title/Abstract])) OR (Maldives[Title/Abstract])) OR (Mexico[Title/Abstract])) OR ("Marshall Islands"[Title/Abstract])) OR ("North Macedonia"[Title/Abstract])) OR (Mali[Title/Abstract])) OR (Myanmar[Title/Abstract])) OR (Myanma[Title/Abstract])) OR (Burma[Title/Abstract])) OR (Montenegro[Title/Abstract])) OR (Mongolia[Title/Abstract])) OR (Mozambique[Title/Abstract])) OR (Mauritania[Title/Abstract])) OR (Mauritius[Title/Abstract])) OR (Malawi[Title/Abstract])) OR (Malaysia[Title/Abstract])) OR (Malaya[Title/Abstract])) OR (Malay[Title/Abstract])) OR (Namibia[Title/Abstract])) OR (Niger[Title/Abstract])) OR (Nigeria[Title/Abstract])) OR (Nicaragua[Title/Abstract])) OR (Nepal[Title/Abstract])) OR (Pakistan[Title/Abstract])) OR (Panama[Title/Abstract])) OR (Peru[Title/Abstract])) OR (Philippines[Title/Abstract])) OR ("Papua New Guinea"[Title/Abstract])) OR (Korea[Title/Abstract])) OR (Paraguay[Title/Abstract])) OR ("West bank"[Title/Abstract])) OR (Gaza[Title/Abstract])) OR (Romania[Title/Abstract])) OR (Russia[Title/Abstract])) OR (Russian[Title/Abstract])) OR ("Russian Federation"[Title/Abstract])) OR (Rwanda[Title/Abstract])) OR (Ruanda[Title/Abstract])) OR (Sudan[Title/Abstract])) OR (Senegal[Title/Abstract])) OR (Solomon[Title/Abstract])) OR ("Solomon Island*"[Title/Abstract])) OR ("Sierra Leone"[Title/Abstract])) OR ("El Salvador"[Title/Abstract])) OR (Serbia[Title/Abstract])) OR (Yugoslavia[Title/Abstract])) OR ("South Sudan"[Title/Abstract])) OR ("São Tomé and Principe"[Title/Abstract])) OR ("Surinam*"[Title/Abstract])) OR (Eswatini[Title/Abstract])) OR ("Syrian Arab Republic"[Title/Abstract])) OR (Syria[Title/Abstract])) OR (Togo[Title/Abstract])) OR ("Togolese Republic"[Title/Abstract])) OR (Thailand[Title/Abstract])) OR (Tajikistan[Title/Abstract])) OR (Tadzhikistan[Title/Abstract])) OR (Tadjikistan[Title/Abstract])) OR (Tadzhik[Title/Abstract])) OR (Turkmenistan[Title/Abstract])) OR (Timor-Leste[Title/Abstract])) OR (Tonga[Title/Abstract])) OR (Tunisia[Title/Abstract])) OR (Turkey[Title/Abstract])) OR (Tuvalu[Title/Abstract])) OR (Tanzania[Title/Abstract])) OR (Uganda[Title/Abstract])) OR (Ukraine[Title/Abstract])) OR (Uzbekistan[Title/Abstract])) OR (Uzbek[Title/Abstract])) OR ("St. Vincent and the Grenadines"[Title/Abstract])) OR (Vietnam[Title/Abstract])) OR (Vanuatu[Title/Abstract])) OR (Samoa[Title/Abstract])) OR (Kosovo[Title/Abstract])) OR (Yemen[Title/Abstract])) OR ("South Africa"[Title/Abstract])) OR (Cape[Title/Abstract])) OR (Zambia[Title/Abstract])) OR (Zimbabwe[Title/Abstract])) OR (Rhodesia[Title/Abstract])) OR (Djibouti[Title/Abstract])) OR ("French Somaliland"[Title/Abstract])) OR (USSR[Title/Abstract])) OR ("Soviet Union"[Title/Abstract])) OR ("Union of Soviet Socialist Republics"[Title/Abstract]) |
| #2 general LMIC terms | (((((((((((((((((((((((((((((((((((((((((((((((((((((((((((((((((((((((((((((((((((((("global south"[Title/Abstract]) OR ("africa south of the sahara"[Title/Abstract])) OR ("sub saharan africa"[Title/Abstract])) OR ("subsaharan africa"[Title/Abstract])) OR ("central africa"[Title/Abstract])) OR ("north africa"[Title/Abstract])) OR ("northern africa"[Title/Abstract])) OR (magreb[Title/Abstract])) OR (maghrib[Title/Abstract])) OR (sahara[Title/Abstract])) OR ("east africa"[Title/Abstract])) OR ("eastern africa"[Title/Abstract])) OR ("west africa"[Title/Abstract])) OR ("western africa"[Title/Abstract])) OR ("west indies"[Title/Abstract])) OR ("indian ocean islands"[Title/Abstract])) OR (caribbean[Title/Abstract])) OR ("central america"[Title/Abstract])) OR ("latin america"[Title/Abstract])) OR ("south america"[Title/Abstract])) OR ("central asia"[Title/Abstract])) OR ("north asia"[Title/Abstract])) OR ("northern asia"[Title/Abstract])) OR ("southeastern asia"[Title/Abstract])) OR ("south eastern asia"[Title/Abstract])) OR ("southeast asia"[Title/Abstract])) OR ("south east asia"[Title/Abstract])) OR ("western asia"[Title/Abstract])) OR ("east europe"[Title/Abstract])) OR ("eastern europe"[Title/Abstract])) OR ("developing countr*"[Title/Abstract])) OR ("developing nation*"[Title/Abstract])) OR ("developing population*"[Title/Abstract])) OR ("developing world"[Title/Abstract])) OR ("less developed countr*"[Title/Abstract])) OR ("less developed nation*"[Title/Abstract])) OR ("less developed world"[Title/Abstract])) OR ("lesser developed countr*"[Title/Abstract])) OR ("lesser developed nation*"[Title/Abstract])) OR ("under developed countr*"[Title/Abstract])) OR ("under developed nation*"[Title/Abstract])) OR ("under developed world"[Title/Abstract])) OR ("underdeveloped countr*"[Title/Abstract])) OR ("underdeveloped nation*"[Title/Abstract])) OR ("underdeveloped population*"[Title/Abstract])) OR ("underdeveloped world"[Title/Abstract])) OR ("middle income countr*"[Title/Abstract])) OR ("middle income nation*"[Title/Abstract])) OR ("middle income population*"[Title/Abstract])) OR ("low income countr*"[Title/Abstract])) OR ("low income nation*"[Title/Abstract])) OR ("low income population*"[Title/Abstract])) OR ("lower income countr*"[Title/Abstract])) OR ("lower income nation*"[Title/Abstract])) OR ("lower income population*"[Title/Abstract])) OR ("underserved countr*"[Title/Abstract])) OR ("underserved nation*"[Title/Abstract])) OR ("underserved population*"[Title/Abstract])) OR ("under served population*"[Title/Abstract])) OR ("deprived countr*"[Title/Abstract])) OR ("deprived population*"[Title/Abstract])) OR ("poor countr*"[Title/Abstract])) OR ("poor nation*"[Title/Abstract])) OR ("poor population*"[Title/Abstract]))) OR ("poor world"[Title/Abstract])) OR ("poorer countr*"[Title/Abstract])) OR ("poorer nation*"[Title/Abstract])) OR ("poorer population*"[Title/Abstract])) OR ("developing econom*"[Title/Abstract])) OR ("less developed econom*"[Title/Abstract])) OR ("underdeveloped econom*"[Title/Abstract])) OR ("middle income econom*"[Title/Abstract])) OR ("low income econom*"[Title/Abstract])) OR ("low gdp"[Title/Abstract])) OR ("low gnp"[Title/Abstract])) OR ("low gross domestic"[Title/Abstract])) OR ("low gross national"[Title/Abstract])) OR ("lower gdp"[Title/Abstract])) OR ("lower gross domestic"[Title/Abstract])) OR (lmic[Title/Abstract])) OR (lmics[Title/Abstract])) OR ("third world"[Title/Abstract])) OR ("lami countr*"[Title/Abstract])) OR ("transitional countr*"[Title/Abstract])) OR ("emerging econom*"[Title/Abstract])) OR ("emerging nation*"[Title/Abstract]) |
| #3 LMIC MESH terms | (poverty[MeSH Terms]) OR (developing countries[MeSH Terms]) |
| #4 ALL LMIC syntax | #1 OR #2 OR #3 |
| **Block 2: non-specialists** | *Rationale:*  *we adapt our definition of non-professional MHC workers from following authors:*  *Purgato et al., 2020* (3)  *Van Ginneken, 2013* (1)  *Javadi, 2017* (4)  *In particular we define non-specialised MHC workers as*  *1. medical workers without extensive education in mental health*  *2. non-medical workers (community workers) without extensive education in mental health*  *we will exlucde: professional specialised mental health care workers such as psychologist, psychiatrist, psychiatric nurse or mental health social worker. Also, we will exclude family members, such as parents or spouses, as the close relationship may have a differential effect.* |
|  | Syntax |
| #5 synonyms for lay-help workers | (((((((((((((("lay worker*"[Title/Abstract]) OR ("lay attendant*"[Title/Abstract])) OR ("lay aid*"[Title/Abstract])) OR ("lay support*"[Title/Abstract])) OR ("lay person*"[Title/Abstract])) OR ("lay helper*"[Title/Abstract])) OR ("lay care giver*"[Title/Abstract])) OR ("lay consultant*"[Title/Abstract])) OR ("lay advisor*"[Title/Abstract])) OR ("lay counsel*"[Title/Abstract])) OR ("lay staff"[Title/Abstract])) OR ("lay assistant*"[Title/Abstract])) OR ("lay volunteer*"[Title/Abstract])) OR ("lay health worker*"[Title/Abstract])) OR ("lay health advisor*"[Title/Abstract]) |
| #6 synonyms for voluntary workers | ((((((("voluntary worker*"[Title/Abstract]) OR ("voluntary aid*"[Title/Abstract])) OR ("voluntary support*"[Title/Abstract])) OR ("voluntary person*"[Title/Abstract])) OR ("voluntary helper*"[Title/Abstract])) OR ("voluntary counsel*"[Title/Abstract])) OR ("voluntary staff"[Title/Abstract])) OR ("voluntary assistant*"[Title/Abstract]) |
| #7 synonyms for community health workers | ((((((((((((("community worker*"[Title/Abstract]) OR ("community aid*"[Title/Abstract])) OR ("community support*"[Title/Abstract])) OR ("community person*"[Title/Abstract])) OR ("community helper*"[Title/Abstract])) OR ("community care giver*"[Title/Abstract])) OR ("community consultant*"[Title/Abstract])) OR ("community advisor*"[Title/Abstract])) OR ("community counsel*"[Title/Abstract])) OR ("community staff"[Title/Abstract])) OR ("community assistant*"[Title/Abstract])) OR ("community outreach worker*"[Title/Abstract])) OR ("community-based practitioner*"[Title/Abstract])) OR ("community based practitioner*"[Title/Abstract]) |
| #8 synonyms for village workers | (((("village worker*"[Title/Abstract]) OR ("village aid*"[Title/Abstract])) OR ("village health worker*"[Title/Abstract])) OR ("village health guide*"[Title/Abstract])) OR ("village health support*"[Title/Abstract]) |
| #9 synonyms for peer counsellors | (("peer counsel*"[Title/Abstract]) OR ("peer educator*"[Title/Abstract])) OR ("peer to peer"[Title/Abstract]) |
| #10 synonyms for untrained workers | ((((("untrained worker*"[Title/Abstract]) OR ("untrained attendant*"[Title/Abstract])) OR ("untrained person*"[Title/Abstract])) OR ("untrained helper*"[Title/Abstract])) OR ("untrained assistant*"[Title/Abstract])) |
| #11 synonyms for non-medical workers | (((("non-medical worker*"[Title/Abstract]) OR ("non-medical support*"[Title/Abstract])) OR ("non-medical person*"[Title/Abstract])) OR ("non-medical consultant*"[Title/Abstract])) OR ("non-medical counsel*"[Title/Abstract]) |
| #12 terms for specific paraprofessionals | ((((((((((nurs*[Title/Abstract]) OR ("home nurs*"[Title/Abstract])) OR ("auxiliary nurs*"[Title/Abstract])) OR ("occupational therapist*"[Title/Abstract])) OR ("physician assistant*"[Title/Abstract])) OR ("birth attendant*"[Title/Abstract])) OR ("nonprofessional home care"[Title/Abstract])) OR ("non-professional home care"[Title/Abstract])) OR (midwife*[Title/Abstract])) OR (midwive*[Title/Abstract])) OR ("social work*"[Title/Abstract]) |
| #13 remaining non-specialist terms | (((((((((((((((Paraprofessional*[Title/Abstract]) OR (Paramedic*[Title/Abstract])) OR ("allied health worker*"[Title/Abstract])) OR ("school staff"[Title/Abstract])) OR (teacher*[Title/Abstract])) OR (trainer*[Title/Abstract])) OR ("lady health worker*"[Title/Abstract])) OR ("health extension worker*"[Title/Abstract])) OR ("home health aid*"[Title/Abstract])) OR ("barefoot doctor*"[Title/Abstract])) OR ("accredited social health activist*"[Title/Abstract])) OR ("health auxiliary worker*"[Title/Abstract])) OR ("front-line health worker*"[Title/Abstract])) OR (Promotora[Title/Abstract])) OR (task-shifting[Title/Abstract])) OR (tasksharing[Title/Abstract]) |
| #14 non-specialists MESH | ((((Home Nursing[MeSH Terms]) OR (Volunteers[MeSH Terms])) OR (community health workers[MeSH Terms])) OR (Allied Health Personnel[MeSH Terms]) ) OR (nurses [MeSH Terms]) OR (Nursing [MeSH Terms]) |
| #15 all non-specialists terms | #5 OR #6 OR #7 OR #8 OR #9 OR #10 OR #11 OR #12 OR #13 OR #14 |
| ***Bloc 3: Technology-based devices*** | *Rationale: We based the search syntax based on two following papers: Agarwal et al.* (5) *and Lehtimaki S. et al.* (6)*.* |
| #16 technology-based devices | (((((((((((((((((((((((((((((((((((((((((("Mobile phone*"[Title/Abstract]) OR ("cell phone*"[Title/Abstract])) OR ("information and communication technology"[Title/Abstract])) OR (technolog*[Title/Abstract])) OR ("cellular phone*"[Title/Abstract])) OR ("mobile device*"[Title/Abstract])) OR (SMS[Title/Abstract])) OR ("text messag*"[Title/Abstract])) OR ("interactive voice response*"[Title/Abstract])) OR (IVR[Title/Abstract])) OR ("Text messaging"[Title/Abstract])) OR (m-health[Title/Abstract])) OR ("mobile health"[Title/Abstract])) OR ("digital assistance"[Title/Abstract])) OR ("electronic diar*"[Title/Abstract])) OR ("medical informatic application*"[Title/Abstract])) OR ("mobile app*"[Title/Abstract])) OR (computer*[Title/Abstract])) OR (smartphone*[Title/Abstract])) OR (ipad*[Title/Abstract])) OR (iphone*[Title/Abstract])) OR (ipod*[Title/Abstract])) OR (Microcomputer*[Title/Abstract])) OR (digital[Title/Abstract])) OR (e-health[Title/Abstract])) OR (web-based[Title/Abstract])) OR (internet-based[Title/Abstract]))) OR ("digital health"[Title/Abstract])) OR (eHealth[Title/Abstract])) OR (mHealth[Title/Abstract])) OR ("internet-based intervention*"[Title/Abstract])) OR ("internet based intervention*"[Title/Abstract])) OR ("web-based intervention*"[Title/Abstract])) OR ("web based intervention*"[Title/Abstract])) OR ("online intervention*"[Title/Abstract])) OR ("internet intervention*"[Title/Abstract])) OR ("digital technolog*"[Title/Abstract])) OR ("cellular telephone*"[Title/Abstract])) OR ("portable cellular phone*"[Title/Abstract])) OR ("mobile phone*"[Title/Abstract])) OR ("mobile telephone*"[Title/Abstract])) OR ("care phone*"[Title/Abstract]) |
| #17 technology-based devices MESH terms | (((digital technology[MeSH Terms]) OR (cell phone[MeSH Terms])) OR (mobile applications[MeSH Terms]) ) OR (Telemedicine[MeSH Terms]) OR (Internet-Based Intervention[MeSH Terms]) |
| #18 ALL digital technology | #16 OR #17 |
| **Block 4: Mental health & illness terms** | *We chose generic mental illness terms, because we focus on mental illness prevention services and treatment.*  *These generic mental illness terms were partly based on Ginniken et al.* (1)*.*  *We additionally added specific terms for anxiety, depression and alcohol use disorder based on the DSM-5 definitions. We chose to specify these disorders, because they were mentioned as the most burdensome mental disorders worldwide in 2019* (7)*:*  *depressive disorders ranked 13*  *alcohol use disorders ranked 20*  *anxiety disorders ranked 24*  *Furthermore, we included generic mental health terms (positive psychology). These terms were selected based on discussions with other researchers and the librarian. We tried to keep the focus on mental health and thus excluded generic health-related terms, such as quality of life etc. as these would go beyond the scope of our search.* |
| #19 generic mental illness terms + specific anxiety, depression and alcohol use terms | ((((((((((((((((((((((((((((((((((((((((((((((((((((((((((((((((((((("psychosocial intervention*"[Title/Abstract]) OR ("psychological side effect*"[Title/Abstract])) OR ("psychosocial factor*"[Title/Abstract])) OR ("psychological factor*"[Title/Abstract])) OR ("psychiatric disease*"[Title/Abstract])) OR ("severe mental disorder*"[Title/Abstract])) OR ("behavioural disorder*"[Title/Abstract])) OR ("psychiatric diagnosis"[Title/Abstract])) OR ("mental health"[Title/Abstract])) OR ("mental disorder*"[Title/Abstract])) OR ("mental issue*"[Title/Abstract])) OR ("psychological distress"[Title/Abstract])) OR ("psychological stress"[Title/Abstract])) OR ("mental stress"[Title/Abstract])) OR ("sleep problem*"[Title/Abstract])) OR ("psychosocial issues"[Title/Abstract])) OR ("psychosocial distress"[Title/Abstract])) OR ("mental distress"[Title/Abstract])) OR ("social-psycholog*"[Title/Abstract])) OR ("subthreshold symptom*"[Title/Abstract])) OR ("subthreshold disorder*"[Title/Abstract])) OR ("subclinical disorder*"[Title/Abstract])) OR ("psychiatric disorder"[Title/Abstract])) OR ("mental Illness"[Title/Abstract])) OR ("mentally ill"[Title/Abstract])) OR ("mentally disabled"[Title/Abstract])) OR ("mentally handicapped"[Title/Abstract])) OR ("mentally retarded"[Title/Abstract])) OR ("mentally disturb*"[Title/Abstract])) OR ("mentally deficient*"[Title/Abstract])) OR ("psychologically ill"[Title/Abstract])) OR ("psychologically handicapped"[Title/Abstract])) OR ("psychologically disturbed"[Title/Abstract])) OR ("psychologically traumati*"[Title/Abstract])) OR ("intellectually disabled"[Title/Abstract])) OR ("intellectually handicapped"[Title/Abstract])) OR ("intellectually retarded"[Title/Abstract])) OR ("intellectually deficient"[Title/Abstract])) OR ("mental retardation"[Title/Abstract])) OR ("mental deficienc*"[Title/Abstract]) OR ("grief"[Title/Abstract]) OR ("grieving"[Title/Abstract]) OR ("emotion*"[Title/Abstract]) OR ("mood disorder*"[Title/Abstract]) OR ("affective disorder*"[Title/Abstract])) OR (depressi*[Title/Abstract])) OR (anxiet*[Title/Abstract])) OR ("post-traumatic stress disorder*"[Title/Abstract])) OR ("post-traumatic stress symptom*"[Title/Abstract])) OR ("major depressive disorder*"[Title/Abstract])) OR (dysthymia*[Title/Abstract])) OR ("major depressive episode*"[Title/Abstract])) OR ("minor depressive episode*"[Title/Abstract])) OR ("dysthymic disorder*"[Title/Abstract])) OR ("selective mutism"[Title/Abstract])) OR ("specific phobia*"[Title/Abstract])) OR (phobia[Title/Abstract])) OR ("social phobia*"[Title/Abstract])) OR ("social anxiet*"[Title/Abstract])) OR ("panic disorder*"[Title/Abstract])) OR (agoraphobia*[Title/Abstract])) OR ("generalized anxiety disorder*"[Title/Abstract])) OR ("generalised anxiety disorder*"[Title/Abstract])) OR ("obsessive-compulsive disorder*"[Title/Abstract])) OR ("body dysmorphic disorder*"[Title/Abstract])) OR ("hoarding disorder*"[Title/Abstract])) OR ("trichotillomania*"[Title/Abstract])) OR ("reactive attachment disorder*"[Title/Abstract])) OR ("disinhibited social engagement disorder*"[Title/Abstract])) OR (PTSD[Title/Abstract])) OR ("acute stress disorder*"[Title/Abstract])) OR ("adjustment disorder*"[Title/Abstract])) OR ("alcohol abuse*"[Title/Abstract])) OR ("alcohol dependenc*"[Title/Abstract])) OR ("alcohol misuse"[Title/Abstract]) |
| #20 generic mental health/well-being terms | ((((((((((("emotional well-being"[Title/Abstract]) OR ("emotional wellbeing"[Title/Abstract])) OR ("psychological well-being"[Title/Abstract])) OR ("psychological wellbeing"[Title/Abstract])) OR ("mental well-being"[Title/Abstract])) OR ("mental wellbeing"[Title/Abstract])) OR ("emotional wellness"[Title/Abstract])) OR ("psychological wellness"[Title/Abstract])) OR ("mental wellness"[Title/Abstract])) OR ("emotional* health*"[Title/Abstract])) OR ("psychological* health*"[Title/Abstract])) OR ("mental* health*"[Title/Abstract]) |
| #21 mental health/illness MESH terms | ((((psychosocial interventions[MeSH Terms]) OR (psychology[MeSH Terms])) OR (mental disorder[MeSH Terms])) OR (Mental Health Recovery[MeSH Terms])) OR (psychiatry[MeSH Terms]) OR (Psychology, Positive [MeSH Terms]) OR (Mental health [MeSH Terms]) |
| #22 all mental health terms together | #19 OR #20 OR #21 |
| #23 all blocks together | First search: #4 AND #15 AND #18 AND #22 (*filter: from inception until 02.12.2021)  Second search: (#4 AND #15 AND #18 AND #22 ) AND (("2021/12/02"[Date - Publication] : "2023/09/18"[Date - Publication])) |

1. van Ginneken N, Tharyan P, Lewin S, et al. Non-specialist health worker interventions for the care of mental, neurological and substance-abuse disorders in low- and middle-income countries. Cochrane Database Syst Rev. 2013; doi: 10.1002/14651858.CD009149.pub2.

2. Sutton A, Campbell F. The ScHARR LMIC filter: Adapting a low- and middle-income countries geographic search filter to identify studies on preterm birth prevention and management. Res Syn Meth. 2022; 13 (4):447-456; doi: 10.1002/jrsm.1552.

3. Purgato M, Uphoff E, Singh R, et al. Promotion, prevention and treatment interventions for mental health in low- And middle-income countries through a task-shifting approach. Epidemiol Psychiatr Sci. 2020; doi: 10.1017/S204579602000061X.

4. Javadi D, Feldhaus I, Mancuso A, Ghaffar A, et al. Applying systems thinking to task shifting for mental health using lay providers: a review of the evidence. Glob Ment Heal. 2017; doi: 10.1017/gmh.2017.15.

5. Agarwal S, Perry HB, Long LA, et al. Evidence on feasibility and effective use of mHealth strategies by frontline health workers in developing countries:

Systematic review. Trop Med Int Heal. 2015; doi: 10.1111/tmi.12525.

6. Lehtimaki S, Martic J, Wahl B, et al. Evidence on digital mental health interventions for adolescents and young people: Systematic overview. JMIR Ment Heal.

2021;8(4): e25847; doi: 10.2196/25847.

7. Abbafati C, Abbas KM, Abbasi-Kangevari M, et al. Global burden of 369 diseases and injuries in 204 countries and territories, 1990–2019: a systematic

analysis for the Global Burden of Disease Study 2019. Lancet. 2020;396(10258):1204–22; doi: https://doi.org/10.1016/S0140-6736(20)30925-9
