# Supplementary material for: The effectiveness of mental health interventions involving non-specialists and digital technology in low-and middle-income countries – a systematic review
Source: BMC Public Health. 2024 Jan 3;24:77. doi: 10.1186/s12889-023-17417-6 (PMC10763181; doi:10.1186/s12889-023-17417-6)
Supplement: Supplementary file 4 — Additional file 4. [file 12889_2023_17417_MOESM4_ESM.docx]

# **ADDITIONAL FILE 4: REASONS FOR EXCLUSION IN FULL-TEXT SCREENING**

**Table S4. Reason for exclusion in the full-text screening**

| **First author name, year of publication and article reference** | **Reason for exclusion** |
| --- | --- |
| Darvish (1), Janevic (2), Fang (3), Baksi (4), Jeekratok (5), Chae (6), Mehrotra (7), Xu (8), Dince (9), Cai, (10), Imamura(11), Latif, 2021 (12), Rahmadiana (13), Hwang(14), Choi (15), Ghanbari (16), Liang(17), Budiyarti (18), Altinbas (19), Cai (20) | No non-specialists |
| Unützer (21), Stasiak (22), MacLean (23), Choi (24), Van Voorhees (25), Stallard (26), O’Connell (27), Nobis (28), Raue (29), Cumming (30), Alexopoulos (31), Smith (32), Fortney (33), Gum (34), Lubans (35) | Wrong setting |
| Sikander (36), Adewuya (37), Patel (38), Gureje (39), Patel (40), Matsuzaka (41), Rahman(42), Gureje (43), Chibanda (44), Dang (45), Acarturk (46), Michelson (47), Hamdani (48), Joag (49), Chen(50), Asher (51), Vinikoor (52) | No digital component |
| Diez-Canseco (53), Nurbaeti (54), Bhardwaj (55), Rocha (56), Evans (57), Tewari (58), Malla (59), Katz (60), Malla (61), Al Mahmud (62), Booc (63), Rodriguez (64) | Wrong study design |
| Chang (65), Wainberg (66) | Wrong outcome |
| Liu (67), Bolbol-Haghighi (68) | No mental health promotion, prevention or treatment focus |
| Poudyal (69), Suleman (70), Sivakumar (71), Li (72) Arjadi (73), Zafar (74), Patel (75), Scazufca (76), Kumar (77), Reynolds (78), Gureje (79), Green (80), Xu (81), Imamura (82) | Protocols identified from registers used for citation chaining |

**References:**

1. Darvish A, Khodadadi-Hassankiadeh N, Abdoosti S, et al.. Effect of Text Messaging-based Psychiatric Nursing Program on Quality of Life in Veterans with Post-Traumatic Stress Disorder: A Randomized Controlled Trial. Int J Community Based Nurs Midwifery. 2019; 7(1):52-62. doi: 10.30476/IJCBNM.2019.40846.

2. Janevic MR, Aruquipa Yujra AC, Marinec N, et al. Feasibility of an interactive voice response system for monitoring depressive symptoms in a lower-middle income Latin American country. Int J Ment Health Syst. 2016;10 (59). doi: https://doi.org/10.1186/s13033-016-0093-3

3. Fang ML, Tapalova O, Zhiyenbayeva N, Kozlovskaya S. Impact of digital game-based learning on the social competence and behavior of preschoolers. Educ Inf Technol. 2021; doi: 10.21203/rs.3.rs-707659/v1

4. Baksi A, Gumus F, Zengin L. Effectiveness of the Preparatory Clinical Education on Nursing Students Anxiety: A Randomized Controlled Trial. Int J Caring Sci. 2017;10(2):1003–12.

5. Jeekratok K, Chanchalor S, Murphy E. Web-based social stories and games for children with autism. Int J Web-Based Learn Teach Technol. 2014; doi: 10.4018/ijwltt.2014100103

6. Chae YM, Park HJ, Cho JG, et al. The reliability and acceptability of telemedicine for patients with schizophrenia in Korea. J Telemed Telecare. 2000; doi: 10.1258/1357633001935095.

7. Mehrotra K, Chand P, Bandawar M, et al. Effectiveness of NIMHANS ECHO blended tele-mentoring model on Integrated Mental Health and Addiction for counsellors in rural and underserved districts of Chhattisgarh, India. Asian J Psychiatr. 2018; doi: https://doi.org/10.3389/fpsyt.2022.869685

8. Xu D, Xiao S, He H, et al. Lay health supporters aided by mobile text messaging to improve adherence, symptoms, and functioning among people with schizophrenia in a resource-poor community in rural China (LEAN): A randomized controlled trial. PLoS Med. 2019; doi: 10.1371/journal.pmed.1002785.

9. Dincer B, Inangil D. The effect of Emotional Freedom Techniques on nurses’ stress, anxiety, and burnout levels during the COVID-19 pandemic: A randomized controlled trial. Explor. 2021;. doi: 10.1016/j.explore.2020.11.012

10. Cai Y, Gong W, He H, Hughes JP, Simoni J, Xiao S, et al. Mobile texting and lay health supporters to improve schizophrenia care in a resource-poor community in rural China (LEAN Trial): Randomized controlled trial extended implementation. J Med Internet Res. 2020; doi: 10.2196/22631.

11. Imamura K, Tran TTT, Nguyen HT, et al. Effect of smartphone-based stress management programs on depression and anxiety of hospital nurses in Vietnam: a three-arm randomized controlled trial. Sci Rep. 2021; doi: https://doi.org/10.1038/s41598-021-90320-5

12. Latif M, Awan F, Gul M, et al. Preliminary evaluation of a culturally adapted CBT-based online programme for depression and anxiety from a lower middle-income country. Cogn Behav Ther. 2021; doi: 10.1017/S1352465820000685.

13. Rahmadiana M, Karyotaki E, Schulte M, et al. Transdiagnostic Internet Intervention for Indonesian University Students With Depression and Anxiety: Evaluation of Feasibility and Acceptability. JMIR Ment Heal. 2021; doi: 10.2196/20036

14. Hwang SY, Kim JS. Risk Factor-tailored Small Group Education for Patients with First-time Acute Coronary Syndrome. Asian Nurs Res (Korean Soc Nurs Sci). 2015; doi: 10.1016/j.anr.2015.07.005.

15. Choi H, Kim S, Ko H, Kim Y, et al. Development and preliminary evaluation of culturally specific web-based intervention for parents of adolescents. J Psychiatr Ment Heal Nurs. 2016; doi: 10.1111/jpm.12327.

16. Ghanbari E, Yektatalab S, Mehrabi M. Effects of Psychoeducational Interventions Using Mobile Apps and Mobile-Based Online Group Discussions on Anxiety and Self-Esteem in Women With Breast Cancer: Randomized Controlled Trial. JMIR Mhealth Uhealth. 2021; doi: 10.2196/19262.

17. Liang D, Han H, Du J, Zhao M, et al. A pilot study of a smartphone application supporting recovery from drug addiction. J Subst Abuse Treat. 2018; doi: https://doi.org/10.1016/j.jsat.2018.02.006

18. Budiyarti L, Agustini N, Hayati H, et al. Effectiveness of web-based play therapy intervention in supporting the development of children with attention deficit/hyperactivity disorder. Pediatr Med Chir. 2023 Mar 28;45(s1). doi: 10.4081/pmc.2023.316. PMID: 36974914.

19. Altinbas BC, Gürsoy A. Nurse-led web-based patient education reduces anxiety in thyroidectomy patients: A randomized controlled study. Int J Nurs Pract. 2023 Jun;29(3):e13131. doi: 10.1111/ijn.13131. Epub 2023 Jan 23. PMID: 36691286.

20. Cai Y, Gong W, He W, et al. Residual Effect of Texting to Promote Medication Adherence for Villagers with Schizophrenia in China: 18-Month Follow-up Survey After the Randomized Controlled Trial Discontinuation. JMIR Mhealth Uhealth. 2022 Apr 19;10(4):e33628. doi: 10.2196/33628. PMID: 35438649; PMCID: PMC9066323.

21. Unützer J, Katon W, Callahan CM, et al. Collaborative care management of late-life depression in the primary care setting: a randomized controlled trial. JAMA. 2002; doi: 10.1001/jama.288.22.2836.

22. Stasiak K, Hatcher S, Frampton C, et al. A pilot double blind randomized placebo controlled trial of a prototype computer-based cognitive behavioural therapy program for adolescents with symptoms of depression. Behav Cogn Psychother. 2014; doi: 10.1017/S1352465812001087

23. MacLean S, Corsi DJ, Litchfield S, et al. Coach-Facilitated Web-Based Therapy Compared With Information About Web-Based Resources in Patients Referred to Secondary Mental Health Care for Depression: Randomized Controlled Trial. J Med Internet Res. 2020; doi: 10.2196/15001

24. Choi NG, Marti CN, Wilson NL, et al. Effect of Telehealth Treatment by Lay Counselors vs by Clinicians on Depressive Symptoms Among Older Adults Who Are Homebound: A Randomized Clinical Trial. JAMA Netw Open. 2020; doi: 10.1001/jamanetworkopen.2020.15648.

25. Van Voorhees BW, Gollan J, Fogel J. Pilot study of Internet-based early intervention for combat-related mental distress. J Rehabil Res Dev. 2012; doi: 10.1682/jrrd.2011.05.0095.

26. Stallard P, Richardson T, Velleman S, et al. Computerized CBT (Think, Feel, Do) for depression and anxiety in children and adolescents: outcomes and feedback from a pilot randomized controlled trial. Behav Cogn Psychother. 2011; doi: 10.1017/S135246581000086X

27. O’Connell J, Shafran R, Pote H. A Randomized Controlled Trial Evaluating the Effectiveness of Face-to-Face and Digital Training in Improving Child Mental Health Literacy Rates in Frontline Pediatric Hospital Staff. Front Psychiatry. 2020;doi: https://doi.org/10.3389/fpsyt.2020.570125

28. Nobis S, Lehr D, Ebert DD, et al. Efficacy of a web-based intervention with mobile phone support in treating depressive symptoms in adults with type 1 and type 2 diabetes: a randomized controlled trial. Diabetes Care. 2015; doi: 10.2337/dc14-1728.

29. Raue PJ, Sirey JA, Dawson A, Berman J, Bruce ML. Lay-delivered behavioral activation for depressed senior center clients: Pilot RCT. Int J Geriatr Psychiatry. 2019;34(11):1715–23.

30. Cumming TM. Social skills of students with emotional disabilities: A technology-based intervention. Diss Abstr Int Sect A Humanit Soc Sci (online). 2007;68(3-A):951. Available: https://digitalscholarship.unlv.edu/cgi/viewcontent.cgi?article=3696&context=rtds (last accessed: 01.10.2023)

31. Alexopoulos GS, Reynolds 3rd CF, Bruce ML, et al. Reducing suicidal ideation and depression in older primary care patients: 24-month outcomes of the PROSPECT study. Am J Psychiatry. 2009; doi: 10.1176/appi.ajp.2009.08121779

32. Smith P, Scott R, Eshkevari E, et al. Computerised CBT for depressed adolescents: Randomised controlled trial. Behav Res Ther. 2015; doi: 10.1016/j.brat.2015.07.009

33. Fortney JC, Pyne JM, Mouden SB, et al. Practice-based versus telemedicine-based collaborative care for depression in rural federally qualified health centers: a pragmatic randomized comparative effectiveness trial. Am J Psychiatry. 2013; doi: https://doi.org/10.1176/appi.ajp.2012.12050696

34. Gum AM, Jensen C, Schonfeld L, et al. A Pilot Study of Brief, Stepped Behavioral Activation for Primary Care Patients with Depressive Symptoms. J Clin Psychol Med Settings. 2022; doi: 10.1007/s10880-022-09864-x.

35. Lubans DR, Smith JJ, Morgan PJ, et al. Mediators of Psychological Well-being in Adolescent Boys. J Adolesc Heal. 2016; doi: 10.1016/j.jadohealth.2015.10.010.

36. Sikander, S., Ahmad, I., Atif, N., et al. Delivering the Thinking Healthy Programme for perinatal depression through volunteer peers: a cluster randomised controlled trial in Pakistan. The Lancet Psychiatry. 2019; doi: 10.1016/S2215-0366(18)30467-X.

37. Adewuya AO, Ola BA, Coker O, et al. A stepped care intervention for non-specialist health workers’ management of depression in the Mental Health in Primary Care (MeHPriC) project, Lagos, Nigeria: A cluster randomised controlled trial. Gen Hosp Psychiatry. 2019; doi: 10.1016/j.genhosppsych.2019.07.012

38. Patel V, Weobong B, Weiss HA, et al. The Healthy Activity Program (HAP), a lay counsellor-delivered brief psychological treatment for severe depression, in primary care in India: a randomised controlled trial. Lancet. 2017; doi:https://doi.org/10.1016/S0140-6736(16)31589-6

39. Gureje O, Oladeji BD, Montgomery AA, et al. High- versus low-intensity interventions for perinatal depression delivered by non-specialist primary maternal care providers in Nigeria: cluster randomised controlled trial (the EXPONATE trial). Br J Psychiatry. 2019; doi: 10.1192/bjp.2019.4

40. Patel V, Weiss HA, Chowdhary N, et al. Effectiveness of an intervention led by lay health counsellors for depressive and anxiety disorders in primary care in Goa, India (MANAS): A cluster randomised controlled trial. Lancet. 2010; doi: 10.1016/S0140-6736(10)61508-5

41. Matsuzaka CT, Wainberg M, Norcini Pala A, et al. Task shifting interpersonal counseling for depression: a pragmatic randomized controlled trial in primary care. BMC Psychiatry. 2017; doi: https://doi.org/10.1186/s12888-017-1379-y

42. Rahman A, Hamdani SU, Awan NR, et al. Effect of a Multicomponent Behavioral Intervention in Adults Impaired by Psychological Distress in a Conflict-Affected Area of Pakistan: A Randomized Clinical Trial. JAMA. 2016; doi: 10.1001/jama.2016.17165.

43. Gureje O, Oladeji BD, Montgomery AA, Bello T, Kola L, Ojagbemi A, et al. Effect of a stepped-care intervention delivered by lay health workers on major depressive disorder among primary care patients in Nigeria (STEPCARE): a cluster-randomised controlled trial. Lancet Glob Heal. 2019; doi: 10.1016/S2214-109X(19)30148-2

44. Chibanda D, Mesu P, Kajawu L, et al. Problem-solving therapy for depression and common mental disorders in Zimbabwe: piloting a task-shifting primary mental health care intervention in a population with a high prevalence of people living with HIV. BMC Public Health. 2011; doi: 10.1186/1471-2458-11-828.

45. Dang H-M, Weiss B, Lam T, et al. Mental health literacy and intervention program adaptation in the internationalization of school psychology for Vietnam. Psychol Sch. 2018; doi: 10.1002/pits.22156

46. Acarturk C, Uygun E, Ilkkursun Z, et al. Group problem management plus (PM+) to decrease psychological distress among Syrian refugees in Turkey: a pilot randomised controlled trial. BMC Psychiatry. 2022; doi: https://doi.org/10.1186/s12888-021-03645-w

47. Michelson D, Malik K, Parikh R, Weiss HA, Doyle AM, Bhat B, et al. Effectiveness of a brief lay counsellor-delivered, problem-solving intervention for adolescent mental health problems in urban, low-income schools in India: a randomised controlled trial. Lancet Child Adolesc Heal. 2020;. doi: https://doi.org/10.1016/S2352-4642(20)30173-5

48. Hamdani SU, Huma Z, Warraitch Aet al. Technology-assisted teachers’ training to promote socioemotional well-being of children in public schools in rural Pakistan. Psychiatr Serv. 2021; doi: 10.1176/appi.ps.202000005

49. Joag K, Shields-Zeeman L, Kapadia-Kundu N, Kawade R, et al. Feasibility and acceptability of a novel community-based mental health intervention delivered by community volunteers in Maharashtra, India: The Atmiyata programme. BMC Psychiatry. 2020; doi: 10.1186/s12888-020-2466-z.

50. Chen S, Conwell Y, He J,et al. Depression care management for adults older than 60 years in primary care clinics in urban China: a cluster-randomised trial. Lancet Psychiatry. 2015;2(4 PG-332–9):332–9. doi: 10.1016/S2215-0366(15)00002-4

51. Asher L, Birhane R, Weiss HA, et al. . Community-based rehabilitation intervention for people with schizophrenia in Ethiopia (RISE): results of a 12-month cluster-randomised controlled trial. Lancet Glob Health. 2022 Apr;10(4):e530-e542. doi: 10.1016/S2214-109X(22)00027-4. Erratum in: Lancet Glob Health. 2022 Jun;10(6):e797. PMID: 35303462; PMCID: PMC8938762.

52. Vinikoor MJ, Sharma A, Murray LK, et al. Alcohol-focused and transdiagnostic treatments for unhealthy alcohol use among adults with HIV in Zambia: A 3-arm randomized controlled trial. Contemp Clin Trials. 2023 Apr;127:107116. doi: 10.1016/j.cct.2023.107116. Epub 2023 Feb 13. PMID: 36791907; PMCID: PMC10065929.

53. Diez-Canseco F, Toyama M, Ipince A, et al. Integration of a technology-based mental health screening program into routine practices of primary health care services in Peru (the Allillanchu Project): Development and implementation. J Med Internet Res. 2018;20(3). doi: 10.2196/jmir.9208.

54. Nurbaeti I, Syafii M, Lestari KB. Developing an android-based application for early detection of postpartum depression symptoms in Indonesia. Belitung Nurs J. 2021;7(2):118–24. doi: https://doi.org/10.33546/bnj.1308

55. Bhardwaj A, Subba P, Rai S, et al. Lessons learned through piloting a community-based SMS referral system for common mental health disorders used by female community health volunteers in rural Nepal. BMC Res Notes. 2020;13(1):309. doi: https://doi.org/10.1186/s13104-020-05148-5

56. Rocha TIU, Aschar SC de AL, Hidalgo-Padilla L, et al. Recruitment, training and supervision of nurses and nurse assistants for a task-shifting depression intervention in two RCTs in Brazil and Peru. Hum Resour Health. 2021;19(1):1–7. doi: 10.1186/s12960-021-00556-5.

57. Evans EC, Deutsch NL, Drake E, et al. Nurse-Patient Interaction as a Treatment for Antepartum Depression: A Mixed-Methods Analysis. J Am Psychiatr Nurses Assoc. 2017;23(5):347–59. Doi: 10.1177/1078390317705449

58. Tewari A, Kallakuri S, Devarapalli S, et al. . Process evaluation of the systematic medical appraisal, referral and treatment (SMART) mental health project in rural India. BMC Psychiatry. 2017;17. doi: 10.1186/s12888-017-1525-6.

59. Malla A, Margoob M, Iyer S, et al. Testing the Effectiveness of Implementing a Model of Mental Healthcare Involving Trained Lay Health Workers in Treating Major Mental Disorders Among Youth in a Conflict-Ridden, Low-Middle Income Environment: Part II Results. Can J Psychiatry. 2019;64(9):630–7. doi: 10.1177/0706743719839314.

60. Katz CL, Washington B, Sacco M, et al. A resident-based telepsychiatry supervision pilot program in Liberia. Psychiatr Serv. 2019;70(3):243–6. doi: 10.1176/appi.ps.201800363.

61. Malla A, Margoob M, Iyer S, et al. A model of mental health care involving trained lay health workers for treatment of major mental disorders among youth in a conflict-ridden, low-middle income environment: Part I adaptation and implementationCan J Psychiatry / La Rev Can Psychiatr. 2019;64(9):621–9. doi: 10.1177/0706743719839318

62. Al Mahmud A, S Abdullah. Poma: A tangible user interface to improve social and cognitive skills of Sri Lankan children with ASD.. Int J Hum Comput Stud. 2020;144. 10.1016/j.ijhcs.2020.102486

63. Booc CER, Diego C, Tee ML, et al. A Mobile Application for Campus-based Psychosocial Wellness Program. 7th Int Conf Information, Intell Syst Appl. 2016.

64. Rodriguez MA, Eisenlohr-Moul TA, Su K, et al. Using non-specialist providers to improve treatment engagement in an online mindfulness intervention in China. Behav Res Ther. 2020;130. Doi:10.1016/j.brat.2020.103644

65. Chang O, Patel VL, Iyengar S, et al. Impact of a mobile-based (mHealth) tool to support community health nurses in early identification of depression and suicide risk in Pacific Island Countries. Australas Psychiatry. 2021 Apr;29(2):200–3. Doi: 10.1177/1039856220956458

66. Wainberg ML, Gouveia ML, Stockton MA, et al. Technology and implementation science to forge the future of evidence- based psychotherapies: the PRIDE scale- up study. Evid Based Ment Healh. 2021;24(1):19–24. doi: 10.1136/ebmental-2020-300199

67. Liu T, Xie S, Wang Y, et al. Effects of App-Based Transitional Care on the Self-Efficacy and Quality of Life of Patients With Spinal Cord Injury in China: Randomized Controlled Trial. JMIR Mhealth Uhealth. 2021;9(4):e22960. doi: 10.2196/22960.

68. Bolbol-Haghighi N, Masoumi SZ, Kazemi F. Effect of Continued Support of Midwifery Students in Labour on the Childbirth and Labour Consequences: A Randomized Controlled Clinical Trial. J Clin Diagn Res. 2016;10(9):Qc14-qc17. doi: 10.7860/JCDR/2016/19947.8495

69. Poudyal A, van Heerden A, Hagaman A, et al. Wearable Digital Sensors to Identify Risks of Postpartum Depression and Personalize Psychological Treatment for Adolescent Mothers: Protocol for a Mixed Methods Exploratory Study in Rural Nepal. JMIR Res Protoc. 2019;8(8):e14734. doi: 10.2196/14734.

70. Suleman A, Mootz JJ, Feliciano P, et al. Scale-Up Study Protocol of the Implementation of a Mobile Health SBIRT Approach for Alcohol Use Reduction in Mozambique. Psychiatr Serv. 2021 Oct;72(10):1199–208. doi: 10.1176/appi.ps.202000086

71. Sivakumar T, Thirthalli J, Kumar CN, et al. Community-Based Rehabilitation for Persons with Severe Mental Illness in a Rural Community of Karnataka: Methodology of a Randomized Controlled Study. Indian J Psychol Med. 2020;42(6 Suppl):S73-s79. doi: 10.1177/0253717620971203.

72. Li YH, Mu TY, Zhang L, et al. Internet-based intervention for postpartum depression in China (“Mommy go”): Protocol for a randomized controlled trial. J Adv Nurs. 2020;76(9):2416–25.

73. Arjadi R, Nauta MH, Scholte WF, et al. Guided Act and Feel Indonesia (GAF-ID) - Internet-based behavioral activation intervention for depression in Indonesia: study protocol for a randomized controlled trial. Trials [online]. 2016; 17(17):1–10. https://search.ebscohost.com/login.aspx?direct=true&db=cin20&AN=118308543&site=ehost-live (last accessed 01.02.2023)

74. Zafar S, Sikander S, Hamdani SU, et al. The effectiveness of Technology-assisted Cascade Training and Supervision of community health workers in delivering the Thinking Healthy Program for perinatal depression in a post-conflict area of Pakistan - study protocol for a randomized controlled trial. Trials [online]. 2016; 6(17):1–8. Available from: https://search.ebscohost.com/login.aspx?direct=true&db=cin20&AN=114353306&site=ehost-live

75. Patel V, Weobong B, Nadkarni A, Weiss HA, Anand A, Naik S, et al. The effectiveness and cost-effectiveness of lay counsellor-delivered psychological treatments for harmful and dependent drinking and moderate to severe depression in primary care in India: PREMIUM study protocol for randomized controlled trials. Trials [Internet]. 2014 Jan;15(1):101. https://search.ebscohost.com/login.aspx?direct=true&db=cin20&AN=104062660&site=ehost-live (last accessed 02.02.2023)

76. Scazufca M, Nakamura CA, Peters TJ, et al. A collaborative care psychosocial intervention to improve late life depression in socioeconomically deprived areas of Guarulhos, Brazil: the PROACTIVE cluster randomised controlled trial protocol. Trials [Online]. 2020 Nov 5;21(1):N.PAG-N.PAG. https://search.ebscohost.com/login.aspx?direct=true&db=cin20&AN=146853498&site=ehost-live (last accessed 02.02.2023)

77. Kumar CN, Chand PK, Manjunatha N, et al. Impact Evaluation of VKN-NIMHANS-ECHO Model of Capacity Building for Mental Health and Addiction: Methodology of Two Randomized Controlled Trials. Indian J Psychol Med. 2020;42(6 Suppl):S80-s86. doi: 10.1177/0253717620969066.

78. Reynolds NR, Satyanarayana V, Duggal M, et al. MAHILA: a protocol for evaluating a nurse-delivered mHealth intervention for women with HIV and psychosocial risk factors in India. BMC Health Serv Res. 2016 4;16:1–9. doi: 10.1186/s12913-016-1605-1.

79. Gureje O, Oladeji BD, Araya R, et al. Expanding care for perinatal women with depression (EXPONATE): Study protocol for a randomized controlled trial of an intervention package for perinatal depression in primary care. BMC Psychiatry. 2015;15. doi: 10.1186/s12888-015-0537-3.

80. Green EP, Pearson N, Rajasekharan S, et al. Expanding Access to Depression Treatment in Kenya Through Automated Psychological Support: Protocol for a Single-Case Experimental Design Pilot Study. JMIR Res Protoc. 2019;8(4):e11800. doi: 10.2196/11800

81. Xu DR, Gong W, Caine ED, et al. Lay health supporters aided by a mobile phone messaging system to improve care of villagers with schizophrenia in Liuyang, China: protocol for a randomised control trial. BMJ Open. 2016;6(1):e010120. doi: 10.1136/bmjopen-2015-010120

82. Imamura K, Tran TTT, Nguyen HT et al. Effects of two types of smartphone-based stress management programmes on depressive and anxiety symptoms among hospital nurses in Vietnam: a protocol for three-arm randomised controlled trial. BMJ Open. 2019;9(4):e025138. Doi: 10.1136/bmjopen-2018-025138.
