# Supplementary material for: The effectiveness of mental health interventions involving non-specialists and digital technology in low-and middle-income countries – a systematic review
Source: BMC Public Health. 2024 Jan 3;24:77. doi: 10.1186/s12889-023-17417-6 (PMC10763181; doi:10.1186/s12889-023-17417-6)
Supplement: Supplementary file 5 — Additional file 5. [file 12889_2023_17417_MOESM5_ESM.docx]

# **ADDITIONAL FILE 5: CALCULATION OF EFFECT SIZES**

This file shows the calculations for Cohen’s d using one outcome from each study as an example. If different approaches were used to compute outcomes from one study, these will be presented as well.

## **A-priori assumptions**

1. Following rules were applied:

- For all outcomes Cohen’s d was calculated.
- In some instances, we additionally used following statistical corrections:
- When sample sizes are small (<20), and standard deviations between the groups are equal, Hedges g’ will be calculated
- If standard deviations between the groups are significantly different, Glass’s delta will be calculated.(1)

1. We use the baseline sample, defined as those participants who did the baseline assessment, in all calculations requiring the sample size.
2. We assumed r = 0.5 in all cases that require this information for the calculations.

**Abbreviations and additional notes**

Df= degrees of freedom

CI= confidence interval

SD= standard deviation

SD(difference)= standard deviation pre-post difference

Pooled SD= pooled standard deviation

SD(within)= Within group standard deviation

SD(Int)= standard deviation of the intervention group

SD(Cont)= standard deviation of the control group

Mean_difference_ refers to the difference in means

SE= standard error

OR= odds ratio

## **Calculations**

### Study: Rahman, 2020

#### Unadjusted outcome: Competence

**Available data:**

Mean in intervention group at follow-up = 44.48

Corresponding SD in intervention group at follow-up = 3.97

Mean in control group at follow-up= 43.63

Corresponding SD in control group at follow-up= 6.34

Unadjusted mean difference between intervention and control group at follow-up= 0.85

Unadjusted corresponding 95% CI lower limit = -1.88

Unadjusted corresponding 95% CI upper limit = 3.59

N intervention group at baseline= 40

N control group at baseline= 40

**Testing assumption of equal variances**

Variance of intervention group = 15.76

Variance of control group= 40.19

**Calculating df numerator and df denominator**

df(numerator) = 39

df (denominator) =39

**Conducting F-test**

$F=\frac{Variance group X (larger number)}{Variance of group Y}$ (2)

F= 2.55

F(39, 39) at alpha 0.025 = 1.89

**Interpretation**

Because F>F(39,39) at alpha = 0.025, we can assume that the variances between the two groups are significantly different.

**Calculating the Glass’s delta**

$\Delta=\frac{difference in mean outcome between groups}{standard deviation of control group}$ (3)

Delta = 0.1340694

**Pooled standard deviation**

$pooled SD\boldsymbol{=}\sqrt{\frac{{SD \left( Int \right)}^{2}+ {SD \left( Cont \right)}^{2}}{\boldsymbol{2}}}$ (4)

Pooled SD= 5.29

**Calculation of Cohen’s d**

Cohens d = $\frac{difference in mean outcome between groups}{standard deviation of outcome among participants}$ (3)

**d= 0.16**

### Study: Muke, 2020 DGT+ intervention

#### Unadjusted outcome: competence

**Available data:**

Mean at baseline = 23.31

Mean at follow-up = 36.02

Pre-post mean change = 12.7

Pre-post SD change = 18.2

Wilcoxon signed ranked test Z score = 2.271

P-value = 0.0231

N at baseline = 14

N at follow-up = 13

**Calculating the standard deviation within groups**

$SD\left( within \right)=\frac{SD(difference)}{\surd2(1-r)}$ (5)

Under the assumption that r= 0.5

Swithin= 18.2

**Calculation of Cohen‘s d**

$d=\frac{difference in mean outcome between groups}{SD(within)}$ (5)

**d=0.7**

**Transforming Cohen’s d to Hedge’s g**

Because of the small sample size (n<20) we will transform Cohens d to Hedges g.

$J=1-\frac{3}{4df-1}$ (5)

and

$g=J x d$ (5)

*Where df= number of comparisons - 1; Hence, using the baseline sample size, we can calculate 14-1=13=df.

Hence g= 0.66

### Study: Muke, 2020 DGT intervention

#### Unadjusted outcome: Competence

**Available data:**

Mean at baseline = 26.45

Mean at follow-up = 29

Pre-post mean change = 2.5

Pre-post SD change = 7.8

Wilcoxon signed ranked test Z score = 0.863

P value = 0.3882

N at baseline = 14

N at follow-up = 12

**Calculating the standard deviation within groups**

$\mathrm{SD}\left( \mathrm{within} \right)=\frac{SD(difference)}{\surd2(1-r)}$ (5)

Under the assumption that r= 0.5

SD(within)= 7.8

**Calculation of Cohen‘s d**

$d=\frac{difference in mean outcome between groups}{SD(within)}$ (5)

**d= 0.32**

**Transforming Cohen’s d to Hedge’s g**

Because of the small sample size (n<20) we will transform Cohens d to Hedges g.

$J=1-\frac{3}{4df-1}$ (5)

and

$g=J x d$ (5)

*Where df= number of comparisons - 1; Hence, using the baseline sample size, we can calculate 14-1=13=df.

Hence g= 0.30

### Study: Nisar, 2020

#### Unadjusted Outcome: Competence

**Available data:**

Mean (SD) intervention group: 42.16 (4.85)

Mean (SD) control group: 42.65 (4.56)

Unadjusted mean difference between intervention and control group = -0.48

SD of outcome among participants (total n): 4.68

N of intervention group at baseline= 50

N of control group at baseline= 50

**Calculation of the unadjusted Cohen’s d**

Cohen’s d = $\frac{difference in mean outcome between groups}{standard deviation of outcome among participants}$ (3)

**d = -0.1**

#### Adjusted outcome: Competence

**Available data:**

Adjusted mean difference= −0.32

Corresponding 95% CI lower limit = -2.23

Corresponding 95% CI upper limit= 1.59

SD intervention group: 4.85

SD control group: 4.56

N intervention group at baseline= 50

N control group at baseline= 50

**Testing the assumption of equal variances**

Variance of intervention group = 23.52

Variance of control group= 20.79

**Calculating df numerator and df denominator**

df(numerator)= 49*

df (denominator)= 49*

**we used the sample with the larger variance (hence the intervention group) as the numerator.*

**Conducting F-test**

$F=\frac{Variance group X (larger number)}{Variance of group Y}$ (2)

F=1.1312375

F(49, 49) at alpha 0.025 = 1.76

**Interpretation**

Because F<F(43,46) at alpha = 0.025, we can assume that the variances between the two groups are not significantly different. Hence, we can assume that the adjusted standard deviation of that outcome is the same in both groups.

**Calculating standard error based on 95% CI**

$SE=\frac{(95\%CI upper limit-lower limit)}{3.92}$ (6)

SE= 0.97

**Calculation of the pooled standard deviation**

$SD\boldsymbol{=}\frac{SE}{\sqrt{\frac{1}{N\left( int \right)}+\frac{1}{N(cont)}}}$ (6)

SD= 4.87

**Calculation of adjusted Cohen’s d**

Cohen‘s d = $\frac{difference in mean outcome between groups}{standard deviation of outcome among participants}$ (3)

**d= -0.1**

### Study: Pereira, 2015 WBIE intervention

#### Adjusted outcome: Knowledge

**Available data:**

Adjusted regression coefficient = 0.44

Adjusted robust standard error = 0.45

Corresponding 95% CI lower limit = -0.6

Corresponding 95% CI upper limit = 1.49

N of intervention group (WBIE) at baseline = 52

N of control (WL) at baseline = 31

**Calculating the SD of the outcome**

$SD=\frac{SE}{\sqrt{\frac{1}{N\left( int \right)}+\frac{1}{N(cont)}}}$ (6)

*we are using the robust SE as SE in this formula

SD= 1.98

**Calculating the SD of the predictor (binary outcome: being allocated to WBIE or WL group)**

$SD=$√np(1-p) (7)

SD= 4.4

**Calculating the unstandardized B from regression coefficient**

$beta1=beta2\frac{SD predictor}{SD outcome}$ (8)

*Where beta1= standardized beta, beta 2= unstandardized beta*

Unstandardized b**=** 0.2

**Calculating Cohens d based on the unstandardized beta**

$d=b/SD$ (9)

**d= 0.1**

### Study: Pereira, 2015 TVBE intervention

#### Adjusted outcome: knowledge

**Available data:**

Adjusted regression coefficient = -0.32

Adjusted robust standard error = 0.72

Corresponding 95% CI lower limit = -1.98

Corresponding 95% CI upper limit = 1.34

N of intervention group (TVBE) at baseline = 32

N of control group (WL) at baseline = 31

**Calculating the SD of the outcome**

$\mathbf{SD=}\frac{\mathbf{SE}}{\sqrt{\frac{1}{N\left( \mathrm{int} \right)}+\frac{1}{N(cont)}}}$ (6)

*we are using the robust SE as SE in this formula

SD= 2.86

**Calculating the SD of the predictor (binary outcome: being allocated to WBIE or WL group)**

$SD=$√np(1-p) (7)

SD= 3.97

**Calculating the unstandardized B from regression coefficient**

$beta1=beta2\frac{SD predictor}{SD outcome}$ (8)

*Where beta1= standardized beta, beta 2= unstandardized beta*

Unstandardized b**=** -0.23

**Calculating Cohen’s d based on the unstandardized beta**

$d=b/SD$ (9)

**d= -0.08**

### Study: Maulik, 2017

#### Unadjusted outcome: MHC-use

**Available data:**

Proportion at baseline = 2

Proportion at follow-up = 30

Sample size of screen positives = 238

**Calculating the unadjusted OR**

|  | MHC-use/event | no MHC use/ no event |
| --- | --- | --- |
| follow-up /exposure | 30 | 208 |
| baseline/ non-exposure | 2 | 236 |

What are the Odds of using MHC after receiving the intervention?

OR= 17.02

**Converting the unadjusted OR to Cohen’s d**

$d=LogOddsRatio\frac{\surd3}{\pi}$ (5)

**d = 0.68**

### Study: Maulik, 2020

**Available data:**

Proportion of MHC user pre-baseline = 30

Proportion of MHC use post-baseline = 731

N participants screened positive = 900

Unadjusted Odds ratio for pre-post difference = 133.3

Unadjusted 95% CI lower limit = 89

Unadjusted 95% CI upper limit = 199.7

Unadjusted P-value = 0.001

Adjusted odds ratio pre-post baseline difference = 137.8

Adjusted 95% CI lower limit = 91.4

Adjusted 95% CI upper limit = 207.7

P-value = 0.001

#### Unadjusted outcome: MHC-use

**Converting unadjusted OR to Cohen’s d**

$d=LogOddsRatio\frac{\surd3}{\pi}$ (5)

**d = 1.17**

#### Adjusted outcome: MHC use

**Converting adjusted OR to Cohen’s d**

$d=LogOddsRatio\frac{\surd3}{\pi}$ (5)

**d = 1.18**

### Study: Doukani, 2021

#### Adjusted outcome: Severity of anxiety

Mean of intervention group at baseline = 11.89

Corresponding SD baseline = 4.72

Mean of intervention group follow-up = 7

Corresponding SD follow-up = 6.65

t(59) value for mean difference in both groups at FU = 3.33

p-value for mean difference in both groups at FU = P < 0.001

N baseline= 60

**Calculating SD change**

$SD\left( difference \right)= \sqrt{SD\left( int \right)+SD \left( cont \right)-2 r SD\left( int \right)SD(cont)}$ (6)

Under the assumption that r= 0.5

SDdiff= 5.93

**Calculating the standard deviation within groups**

$SD\left( within \right)=\frac{SD(difference)}{\surd2(1-r)}$ (5)

Under the assumption that r= 0.5

SD(within)= 5.93

**Calculation the mean change score between FU and baseline**

Mean_change_= -4.89

**Calculation of Cohen’s d**

$d=\frac{difference in mean outcome between groups}{SD(within)}$ (5)

**d= -0.83**

#### Adjusted outcome: Severity of depression

Mean of intervention group at baseline = 11.86

Corresponding SD baseline = 6.28

Mean of intervention group follow-up = 6.62

Corresponding SD follow-up = 6.13

t(59) value for mean difference in both groups at FU = 3.98

p-value for mean difference in both groups at FU = P < 0.001

N baseline= 80

**Calculating SD change**

$SD\left( difference \right)= \sqrt{SD\left( int \right)+SD \left( cont \right)-2 r SD\left( int \right)SD(cont)}$ (6)

Under the assumption that r= 0.5

SD(difference)= 6.21

**Calculating the standard deviation within groups**

$SD\left( within \right)=\frac{SD(difference)}{\surd2(1-r)}$ (5)

Under the assumption that r= 0.5

SD(within)= 6.21

**Calculation the mean change score between FU and baseline**

Mean_change_= -5.24

**Calculation of Cohen’s d**

$d=\frac{difference in mean outcome between groups}{SD(within)}$ (5)

**d= -0.84**

#### Adjusted outcome: Severity of CMD

Mean of intervention group at baseline = 12.13

Corresponding SD baseline = 2.75

Mean of intervention group follow-up = 7.5

Corresponding SD follow-up = 5.33

t(59) value for mean difference in both groups at FU = 6.94

p-value for mean difference in both groups at FU = P < 0.001

N baseline= 60

**Calculating SD change**

$S\left( difference \right)= \sqrt{SD\left( int \right)+SD \left( cont \right)-2 r SD\left( int \right)SD(cont)}$ (6)

Under the assumption that r= 0.5

SD(difference)= 4.62

**Calculating the standard deviation within groups**

$SD\left( within \right)=\frac{SD(difference)}{\surd2(1-r)}$ (5)

Under the assumption that r= 0.5

SD(within)= 4.62

**Calculation the mean change score between FU and baseline**

Mean_change_= -4.63

**Calculation of Cohen’s d**

$d=\frac{difference in mean outcome between groups}{SD(within)}$ (5)

d= -1

### Study: Dambi, 2022 Inuka intervention

#### Unadjusted outcome: Severity of CMD

**Available data:**

intervention baseline mean = 8

intervention baseline SD = 2.6

intervention follow-up mean = 5.4

intervention follow-up SD = 2.1

baseline intervention n = 45

**Calculating SD change**

$SD\left( difference \right)= \sqrt{SD\left( int \right)+SD \left( cont \right)-2 r SD\left( int \right)SD(cont)}$ (6)

Under the assumption that r= 0.5

SDdiff= 2.39

**Calculating the standard deviation within groups**

$SD\left( within \right)=\frac{SD(difference)}{\surd2(1-r)}$ (5)

Under the assumption that r= 0.5

SD(within)= 2.39

**Calculation the mean change score between FU and baseline**

Mean_change_= -2.6

**Calculation of Cohen’s d**

$d=\frac{difference in mean outcome between groups}{SD(within)}$ (5)

d= -1.08

#### Unadjusted outcome: Severity of depression

**Available data:**

intervention baseline mean = 10.4

intervention baseline SD = 5.5

intervention follow-up mean = 6.9

intervention follow-up SD = 4

baseline intervention n = 45

**Calculating SD change**

$SD\left( difference \right)= \sqrt{SD\left( int \right)+SD \left( cont \right)-2 r SD\left( int \right)SD(cont)}$ (6)

Under the assumption that r= 0.5

SD(difference)= 4.92

**Calculating the standard deviation within groups**

$SD\left( within \right)=\frac{SD(difference)}{\surd2(1-r)}$ (5)

Under the assumption that r= 0.5

SD(within)= 4.92

**Calculation the mean change score between FU and baseline**

Mean_change_= -3.5

**Calculation of Cohen’s d**

$d=\frac{difference in mean outcome between groups}{SD(within)}$ (5)

d= -0.71

#### Unadjusted outcome: Severity of anxiety

**Available data:**

intervention baseline mean = 10.5

intervention baseline SD = 5.8

intervention follow-up mean = 7.2

intervention follow-up SD = 4.4

baseline intervention n = 45

**Calculating SD change**

$SD\left( difference \right)= \sqrt{SD\left( int \right)+SD \left( cont \right)-2 r SD\left( int \right)SD(cont)}$ (6)

Under the assumption that r= 0.5

SD(difference)= 5.24

**Calculating the standard deviation within groups**

$SD\left( within \right)=\frac{SD(difference)}{\surd2(1-r)}$ (5)

Under the assumption that r= 0.5

SD(within)= 5.24

**Calculation the mean change score between FU and baseline**

Mean_change_= -3.3

**Calculation of Cohen’s d**

$d=\frac{difference in mean outcome between groups}{SD(within)}$ (5)

d= -0.63

#### Unadjusted outcome: disability

**Available data:**

intervention baseline mean = 23.1

intervention baseline SD = 9

intervention follow-up mean = 18.4

intervention follow-up SD = 5.1

baseline intervention n = 45

**Calculating SD change**

$SD\left( difference \right)= \sqrt{SD\left( int \right)+SD \left( cont \right)-2 r SD\left( int \right)SD(cont)}$ (6)

Under the assumption that r= 0.5

SD(difference)= 7.82

**Calculating the standard deviation within groups**

$SD\left( within \right)=\frac{SD(difference)}{\surd2(1-r)}$ (5)

Under the assumption that r= 0.5

SD(within)= 7.82

**Calculation the mean change score between FU and baseline**

Mean_change_= -4.7

**Calculation of Cohen’s d**

$d=\frac{difference in mean outcome between groups}{SD(within)}$ (5)

d= -0.6

#### Unadjusted outcome: Quality of life

**Available data:**

intervention baseline mean = 0.807

intervention baseline SD = 0.139

intervention follow-up mean = 0.859

intervention follow-up SD = 0.124

baseline intervention n = 45

**Calculating SD change**

$SD\left( difference \right)= \sqrt{SD\left( int \right)+SD \left( cont \right)-2 r SD\left( int \right)SD(cont)}$ (6)

Under the assumption that r= 0.5

SD(difference)= 0.13

**Calculating the standard deviation within groups**

$SD\left( within \right)=\frac{SD(difference)}{\surd2(1-r)}$ (5)

Under the assumption that r= 0.5

SD(within)= 0.13

**Calculation the mean change score between FU and baseline**

Mean_change_= 0.05

**Calculation of Cohen’s d**

$d=\frac{difference in mean outcome between groups}{SD(within)}$ (5)

d= 0.39

### Study: Dambi, 2022 Friendship bench Whatsapp Intervention

#### Unadjusted outcome: Severity of CMD

**Available data:**

intervention baseline mean = 8.1

intervention baseline SD = 2

intervention follow-up mean = 6.48

intervention follow-up SD = 1.6

baseline intervention n = 31

**Calculating SD change**

$SD\left( difference \right)= \sqrt{SD\left( int \right)+SD \left( cont \right)-2 r SD\left( int \right)SD(cont)}$ (6)

Under the assumption that r= 0.5

SD(difference)= 1.83

**Calculating the standard deviation within groups**

$SD\left( within \right)=\frac{SD(difference)}{\surd2(1-r)}$ (5)

Under the assumption that r= 0.5

SD(within)= 1.83

**Calculation the mean change score between FU and baseline**

Mean_change_= -1.62

**Calculation of Cohen’s d**

$d=\frac{difference in mean outcome between groups}{SD(within)}$ (5)

d= -0.88

#### Unadjusted outcome: Severity of depression

**Available data:**

intervention baseline mean = 13.2

intervention baseline SD = 2.3

intervention follow-up mean = 9,7

intervention follow-up SD = 2.8

baseline intervention n = 31

**Calculating SD change**

$SD\left( difference \right)= \sqrt{SD\left( int \right)+SD \left( cont \right)-2 r SD\left( int \right)SD(cont)}$ (6)

Under the assumption that r= 0.5

SD(difference)= 2.58

**Calculating the standard deviation within groups**

$SD\left( within \right)=\frac{SD(difference)}{\surd2(1-r)}$ (5)

Under the assumption that r= 0.5

SD(within)= 2.58

**Calculation the mean change score between FU and baseline**

Mean_change_= -3.5

**Calculation of Cohen’s d**

$d=\frac{difference in mean outcome between groups}{SD(within)}$ (5)

d= -1.35

#### Unadjusted outcome: Severity of anxiety

**Available data:**

intervention baseline mean = 12.1

intervention baseline SD = 2.7

intervention follow-up mean = 9.1

intervention follow-up SD = 2.7

baseline intervention n = 31

**Calculating SD change**

$SD\left( difference \right)= \sqrt{SD\left( int \right)+SD \left( cont \right)-2 r SD\left( int \right)SD(cont)}$ (6)

Under the assumption that r= 0.5

SD(difference)= 2.7

**Calculating the standard deviation within groups**

$SD\left( within \right)=\frac{SD(difference)}{\surd2(1-r)}$ (5)

Under the assumption that r= 0.5

SD(within)= 2.7

**Calculation the mean change score between FU and baseline**

Mean_change_= -3

**Calculation of Cohen’s d**

$d=\frac{difference in mean outcome between groups}{SD(within)}$ (5)

d= -1.1

#### Unadjusted outcome: disability

**Available data:**

intervention baseline mean = 22.1

intervention baseline SD = 5.4

intervention follow-up mean = 17.8

intervention follow-up SD = 2.1

baseline intervention n = 31

**Calculating SD change**

$SD\left( difference \right)= \sqrt{SD\left( int \right)+SD \left( cont \right)-2 r SD\left( int \right)SD(cont)}$ (6)

Under the assumption that r= 0.5

SD(difference)=4.71

**Calculating the standard deviation within groups**

$SD\left( within \right)=\frac{SD(difference)}{\surd2(1-r)}$ (5)

Under the assumption that r= 0.5

S_within_= 4.71

**Calculation the mean change score between FU and baseline**

Mean_change_= -4.3

**Calculation of Cohen’s d**

$d=\frac{difference in mean outcome between groups}{SD(within)}$ (5)

d= -0.91

#### Unadjusted outcome: Quality of life

**Available data:**

intervention baseline mean = 0.745

intervention baseline SD =1.69

intervention follow-up mean = 0.85

intervention follow-up SD = 0.077

baseline intervention n = 31

**Calculating SD change**

$SD\left( difference \right)= \sqrt{SD\left( int \right)+SD \left( cont \right)-2 r SD\left( int \right)SD(cont)}$ (6)

Under the assumption that r= 0.5

SD(difference)= 1.65

**Calculating the standard deviation within groups**

$SD\left( within \right)=\frac{SD(difference)}{\surd2(1-r)}$ (5)

Under the assumption that r= 0.5

SD_within_= 1.65

**Calculation the mean change score between FU and baseline**

Mean_change_= 0.11

**Calculation of Cohen’s d**

$d=\frac{difference in mean outcome between groups}{SD(within)}$ (5)

d= 0.06

### Study: Chibanda, 2016

#### Unadjusted outcome: Severity of CMD

**Available data:**

Mean in intervention group at follow-up= 3.81

Corresponding 95% CI lower limit = 3.28

Corresponding 95% CI upper limit = 4.34

Mean in control group at follow-up = 8.9

Corresponding 95% CI lower limit = 8.33

Corresponding 95% CI upper limit = 9.47

Unadjusted mean difference between intervention & control group at follow-up= -5.09

Unadjusted corresponding 95% CI lower limit = -5.86

Unadjusted corresponding 95% CI upper limit = 4.31

N intervention group at baseline = 286

N control group at baseline= 287

**Calculating the intervention SD based on the 95% CI**

$SD= \sqrt{N} x\left( upper limit-lower limit \right)/3.92$ (6)

SD= 4.57

**Calculating the control SD at 6 months from the 95% CI**

$SD= \sqrt{N} x\left( upper limit-lower limit \right)/3.92$ (6)

SD= 4.93

**Testing assumption of equal variance**

Variance of intervention group = 19.01

Variance of control group = 22.07

**Calculating df numerator and df denominator**

df(numerator) = 286

df (denominator) =285

**Conducting F-test**

$F=\frac{Variance group X (larger number)}{Variance of group Y}$ (2)

F= 1.26173

F(29, 29) at alpha 0.025 = 1.28

**Interpretation**

Because F<F (260,259) at alpha = 0.025, we can assume that the variances between the two groups are not significantly different.

**Calculating the standard error based on 95% CI**

$SE=\frac{(95\%CI upper limit-lower limit)}{3.92}$ (6)

SE=0.4

**Calculating the unadjusted within-group standard deviation**

$SD\boldsymbol{=}\frac{SE}{\sqrt{\frac{1}{N\left( int \right)}+\frac{1}{N(cont)}}}$ (6)

SD= 4.73

**Calculating of Cohen’s**

d = $\frac{difference in mean outcome between groups}{standard deviation of outcome among participants}$ (6)

d= -1.07

#### Adjusted outcome: Severity of CMD

Available data:

Adjusted mean difference between intervention & control group = -4.86

Unadjusted corresponding 95% CI lower limit = -5.63

Unadjusted corresponding 95% CI upper limit = -4.1

n intervention group at baseline = 286

n control group at baseline = 287

**Calculating standard error based on 95% CI**

$SE=\frac{(95\%CI upper limit-lower limit)}{3.92}$ (6)

SE= 0.39

**Calculation of the pooled standard deviation**

$SD\boldsymbol{=}\frac{SE}{\sqrt{\frac{1}{N\left( int \right)}+\frac{1}{N(cont)}}}$ (6)

SD= 4.67

**Calculation of adjusted Cohen’s d**

Cohen‘s d = $\frac{difference in mean outcome between groups}{standard deviation of outcome among participants}$ (3)

**d= -1.04**

### Study: Ross, 2013

#### Unadjusted outcome: Severity of depression at 1 month

**Available data:**

Mean in intervention group at baseline = 25.3

Corresponding SD in intervention group at baseline = 11.63

Mean in intervention group at follow-up= 20.1

Corresponding SD in intervention group at follow-up = 9.43

Mean in control group at baseline = 18.4

Corresponding SD in control group at baseline = 8.85

Mean in control group at follow-up = 17.8

Corresponding SD in control at follow-up = 7.83

N intervention group at baseline = 20

N control group at baseline = 20

**Calculating mean difference between intervention and control group at FU**

Mean_difference_= 2.3

**Calculating the Variance**

Variance of intervention group = 88.92

Variance of control group= 61.31

**Calculating df numerator and df denominator**

df(numerator)= 19

df (denominator)= 19

**Conducting F-test**

$F=\frac{Variance group X (larger number)}{Variance of group Y}$ (2)

F= 1.45

F(19, 19) at alpha 0.025 = 2.53

**Interpretation**

Because F<F(19,19) at alpha = 0.025, we can assume that the variances between the two groups are not significantly different.

**Pooling the standard deviations**

$pooled SD\boldsymbol{=}\sqrt{\frac{{SD \left( Int \right)}^{2}+ {SD \left( Cont \right)}^{2}}{\boldsymbol{2}}}$ (4)

Pooled SD= 8.67

**Calculating of Cohen’s d**

d = $\frac{difference in mean outcome between groups}{standard deviation of outcome among participants}$ (6)

**d= 0.27**

#### Unadjusted outcome: Severity of depression at 2 month

**Available data:**

Mean in intervention group at baseline = 25.3

Corresponding SD in intervention group at baseline = 11.63

Mean in intervention group at follow-up= 18.09

Corresponding SD in intervention group at follow-up = 8.63

Mean in control group at baseline = 18.4

Corresponding SD in control group at baseline = 8.85

Mean in control group at follow-up = 21.2

Corresponding SD in control at follow-up = 10.26

N intervention group at baseline = 20

N control group at baseline = 20

**Calculating mean difference between intervention and control group at FU**

Mean_difference_= -2.3

**Calculating the Variance**

Variance of intervention group = 74.48

Variance of control group= 105.27

**Calculating df numerator and df denominator**

df(numerator)= 19

df (denominator)= 19

**Conducting F-test**

$F=\frac{Variance group X (larger number)}{Variance of group Y}$ (2)

F= 1.41

F(19, 19) at alpha 0.025 = 2.53

**Interpretation**

Because F<F(19,19) at alpha = 0.025, we can assume that the variances between the two groups are not significantly different.

**Pooling the standard deviations**

$pooled SD\boldsymbol{=}\sqrt{\frac{{SD \left( Int \right)}^{2}+ {SD \left( Cont \right)}^{2}}{\boldsymbol{2}}}$ (4)

Pooled SD= 9.48

**Calculating of Cohen’s d**

d = $\frac{difference in mean outcome between groups}{standard deviation of outcome among participants}$ (6)

**d= -0.24**

### Study: Ebrahem

#### Unadjusted outcome: Presence of depression

**Available data:**

normal depression pre-intervention n=63

mild depression pre-intervention n=68

moderate depression pre-intervention n=49

severe depression pre-intervention n=29

normal depression post-intervention n=121

mild depression post-intervention n=52

moderate depression post-intervention n=34

severe depression post-intervention n=2

**Calculating the unadjusted OR**

|  | Depression (mild-severe)/event | Normal depression/ no event |
| --- | --- | --- |
| follow-up /exposure | 88 | 121 |
| baseline/ non-exposure | 146 | 36 |

What are the Odds of having depression (mild-severe) after receiving the intervention?

OR= 0.31

**Converting the unadjusted OR to Cohen’s d**

$d=LogOddsRatio\frac{\surd3}{\pi}$ (5)

**d = -0.28**

#### Unadjusted outcome: Presence of anxiety

**Available data:**

normal anxiety pre-intervention n=79

mild anxiety pre-inervention n= 14

moderate anxiety pre-intervention n=63

severe anxiety pre-intervention n=13

extremely severe anxiety pre-intervention n=40

normal anxiety post-intervention n=139

mild anxiety post-inervention n=25

moderate anxiety post-intervention n=33

severe anxiety post-intervention n=6

extremely severe anxiety post-intervention n=6

**Calculating the unadjusted OR**

|  | Anxiety (mild- extremely severe)/event | Normal anxiety/ no event |
| --- | --- | --- |
| follow-up /exposure | 70 | 139 |
| baseline/ non-exposure | 130 | 79 |

What are the Odds of having anxiety (mild-extremely severe) after receiving the intervention?

OR= 0.31

**Converting the unadjusted OR to Cohen’s d**

$d=LogOddsRatio\frac{\surd3}{\pi}$ (5)

**d = -0.28**

#### Unadjusted outcome: Presence of stress

**Available data:**

normal stress pre-intervention n=131

mild stress pre-intervention n=24

moderate stress pre-intervention n=7

severe stress pre-intervention n=47

normal stress post-intervention n=178

mild stress post-intervention n=22

moderate stress post-intervention n=2

severe stress post-intervention n=7

**Calculating the unadjusted OR**

|  | Stress (mild- severe)/event | Normal stress/ no event |
| --- | --- | --- |
| follow-up /exposure | 31 | 178 |
| baseline/ non-exposure | 78 | 131 |

What are the Odds of having stress (mild- severe) after receiving the intervention?

OR= 0.29

**Converting the unadjusted OR to Cohen’s d**

$d=LogOddsRatio\frac{\surd3}{\pi}$ (5)

**d = -0.29**

### Study: Scazufca, 2019

#### Unadjusted outcome: Severity of depression

**Available data:**

Mean of intervention group at baseline = 15.5

SD of intervention group at baseline = 3.5

Mean of intervention group at follow-up= 3.8

SD of intervention group at follow-up = 3.9

Mean of control group at baseline = 13.9

SD of control group at baseline = 3.7

Mean of control group at follow-up = 12.3

SD of control group at follow-up = 3.7

Mean of both groups at follow-up =7.4

SD of both groups at follow-up = 5.7

N intervention group at baseline = 33

N intervention group at follow-up = 31

N control group at baseline = 25

N control group at follow-up = 23

**Calculating the mean difference at follow-up**

Mean_difference_= -8.5

SD of outcome among participants **=** 5.7

**Calculation of Cohen’s**

d = $\frac{difference in mean outcome between groups}{standard deviation of outcome among participants}$ (6)

**d= -1.49**

#### **Unadjusted outcome: Quality of life**

**Available data:**

Mean of intervention group at baseline = 0.71

SD of intervention group at baseline = 0.16

Mean of intervention group at follow-up = 0.81

SD of intervention group at follow-up = 0.14

Mean of control group at baseline = 0.81

SD of control group at baseline = 0.13

Mean of control group at follow-up = 0.82

SD of control group at follow-up = 0.13

mean of both groups at follow-up = 0.8156

SD of both groups at follow-up = 0.14

N of intervention group at baseline = 33

N of intervention group at follow-up = 31

N of control group at baseline = 25

N of control group at follow-up = 23

**Calculating the mean difference at follow-up**

Mean_difference_= -0.008

SD of outcome among participants **=** 0.14

**Calculation of Cohen’s**

d = $\frac{difference in mean outcome between groups}{standard deviation of outcome among participants}$ (6)

**d= -0.06**

#### Unadjusted outcome: Capability

**Available data:**

Mean of intervention group at baseline = 0.56

SD of intervention group at baseline = 0.18

Mean of intervention group at follow-up = 0.73

SD of intervention group at follow-up = 0.16

Mean of control group at baseline = 0.81

SD of control group at baseline = 0.13

Mean of control group at follow-up = 0.71

SD of control group at follow-up = 0.15

mean of both groups at follow-up = 0.72

SD of both groups at follow-up = 0.15

N of intervention group at baseline = 33

N of intervention group at follow-up = 31

N of control group at baseline = 25

N of control group at follow-up = 23

**Calculating the mean difference at follow-up**

Mean_difference_= 0.026

SD of outcome among participants **=** 0.15

**Calculation of Cohen’s**

d = $\frac{difference in mean outcome between groups}{standard deviation of outcome among participants}$ (6)

**d= 0.17**

### Study: Garg, 2022

#### Unadjusted outcome: Severity of CMD

**Available data:**

intervention baseline mean = 6.31

intervention baseline SD = 2.44

intervention follow-up mean = 3.31

intervention follow-up SD = 2.81

baseline intervention n = 161

**Calculating SD change**

$SD\left( difference \right)= \sqrt{SD\left( int \right)+SD \left( cont \right)-2 r SD\left( int \right)SD(cont)}$ (6)

Under the assumption that r= 0.5

SD(difference)= 2.64

**Calculating the standard deviation within groups**

$SD\left( within \right)=\frac{SD(difference)}{\surd2(1-r)}$ (5)

Under the assumption that r= 0.5

SD(within)= 2.64

**Calculation the mean change score between FU and baseline**

Mean_change_= -3

**Calculation of Cohen’s d**

$d=\frac{difference in mean outcome between groups}{SD(within)}$ (5)

d= -1.13

#### Unadjusted outcome: Alcohol use disorder

**Available data:**

intervention baseline mean = 21.5

intervention baseline SD = 2.35

intervention follow-up mean = 9.83

intervention follow-up SD = 4.17

baseline intervention n = 161

**Calculating SD change**

$SD\left( difference \right)= \sqrt{SD\left( int \right)+SD \left( cont \right)-2 r SD\left( int \right)SD(cont)}$ (6)

Under the assumption that r= 0.5

SD(difference)= 3.62

**Calculating the standard deviation within groups**

$SD\left( within \right)=\frac{SD(difference)}{\surd2(1-r)}$ (5)

Under the assumption that r= 0.5

SD(within)= 3.62

**Calculation the mean change score between FU and baseline**

Mean_change_= -11.67

**Calculation of Cohen’s d**

$d=\frac{difference in mean outcome between groups}{SD(within)}$ (5)

d= -3.22

#### Unadjusted outcome: Disability and functioning

**Available data:**

intervention baseline mean = 23.77

intervention baseline SD = 9.25

intervention follow-up mean = 18.12

intervention follow-up SD = 6.45

baseline intervention n = 161

**Calculating SD change**

$SD\left( difference \right)= \sqrt{SD\left( int \right)+SD \left( cont \right)-2 r SD\left( int \right)SD(cont)}$ (6)

Under the assumption that r= 0.5

SD(difference)= 8.22

**Calculating the standard deviation within groups**

$SD\left( within \right)=\frac{SD(difference)}{\surd2(1-r)}$ (5)

Under the assumption that r= 0.5

SD(within)= 8.22

**Calculation the mean change score between FU and baseline**

Mean_change_= -5.65

**Calculation of Cohen’s d**

$d=\frac{difference in mean outcome between groups}{SD(within)}$ (5)

d= -0.69

### Study: Liu, 2023

#### Unadjusted outcome: Severity of depression immediately after intervention

**Available data:**

intervention baseline mean = 15.41

intervention baseline SD = 12.64

intervention follow-up mean = 15.65

intervention follow-up SD = 11.38

mean change of intervention group = 0.24

SD change of intervention group = 9.031

control baseline mean = 14.1

control baseline SD = 13.7

control follow-up mean = 16.04

control follow-up SD = 11.19

mean change of control group = 1.94

SD change of control group = 9.986

Baseline intervention n = 49

baseline control n = 49

difference in mean change between both groups = -1.7

**Calculating the Variance**

Variance of intervention group = 81.56

Variance of control group= 99.72

**Calculating df numerator and df denominator**

df(numerator)= 48

df (denominator)= 48

**Conducting F-test**

$F=\frac{Variance group X (larger number)}{Variance of group Y}$ (2)

F= 1.22

F(48, 48) at alpha 0.025 = 1.77

**Interpretation**

Because F<F(48,48) at alpha = 0.025, we can assume that the variances between the two groups are not significantly different.

**Pooling the standard deviations**

$pooled SD\boldsymbol{=}\sqrt{\frac{{SD \left( Int \right)}^{2}+ {SD \left( Cont \right)}^{2}}{\boldsymbol{2}}}$ (4)

Pooled SD= 9.52

**Calculating of Cohen’s d**

d = $\frac{difference in mean outcome between groups}{standard deviation of outcome among participants}$ (6)

**d= -0.18**

#### Unadjusted outcome: Severity of depression immediately after intervention

**Available data:**

intervention baseline mean = 15.41

intervention baseline SD = 12.64

intervention follow-up mean = 14.39

intervention follow-up SD = 12.79

mean change of intervention group = -1.02

SD change of intervention group = 10.816

control baseline mean = 14.1

control baseline SD = 13.7

control follow-up mean = 18.84

control follow-up SD = 12.68

mean change of control group = 4.74

SD change of control group = 10.692

Baseline intervention n = 49

baseline control n = 49

difference in mean change between both groups = -5.76

**Calculating the Variance**

Variance of intervention group = 116.99

Variance of control group= 114.32

**Calculating df numerator and df denominator**

df(numerator)= 48

df (denominator)= 48

**Conducting F-test**

$F=\frac{Variance group X (larger number)}{Variance of group Y}$ (2)

F= 1.02

F(48, 48) at alpha 0.025 = 1.77

**Interpretation**

Because F<F(48,48) at alpha = 0.025, we can assume that the variances between the two groups are not significantly different.

**Pooling the standard deviations**

$pooled SD\boldsymbol{=}\sqrt{\frac{{SD \left( Int \right)}^{2}+ {SD \left( Cont \right)}^{2}}{\boldsymbol{2}}}$ (4)

Pooled SD= 10.76

**Calculating of Cohen’s d**

d = $\frac{difference in mean outcome between groups}{standard deviation of outcome among participants}$ (6)

**d= -0.54**

### Study: Ötzoprak, 2023

#### Unadjusted outcome: Quality of life 10 weeks after baseline

**Available data:**

intervention follow-up mean = 27.85

intervention follow-up SD = 1.14

control follow-up mean = 25.29

control follow-up SD = 1.15

Baseline intervention n = 31

baseline control n = 30

**Calculating the Variance**

Variance of intervention group = 57

Variance of control group= 152.77

**Calculating df numerator and df denominator**

df(numerator)= 29

df (denominator)= 30

**Conducting F-test**

$F=\frac{Variance group X (larger number)}{Variance of group Y}$ (2)

F= 1.02

F(29, 30) at alpha 0.025 = 2.08

**Interpretation**

Because F<F(29,30) at alpha = 0.025, we can assume that the variances between the two groups are not significantly different.

**Pooling the standard deviations**

$pooled SD\boldsymbol{=}\sqrt{\frac{{SD \left( Int \right)}^{2}+ {SD \left( Cont \right)}^{2}}{\boldsymbol{2}}}$ (4)

Pooled SD= 1.15

**Calculating of Cohen’s d**

d = $\frac{difference in mean outcome between groups}{standard deviation of outcome among participants}$ (6)

**d= 2.24**

#### Unadjusted outcome: Quality of life 16 weeks after baseline

**Available data:**

intervention follow-up mean = 29.3

intervention follow-up SD = 0.45

control follow-up mean = 27.1

control follow-up SD = 0.8

Baseline intervention n = 31

baseline control n = 30

**Calculating the Variance**

Variance of intervention group = 0.2

Variance of control group= 0.46

**Calculating df numerator and df denominator**

df(numerator)= 29

df (denominator)= 30

**Conducting F-test**

$F=\frac{Variance group X (larger number)}{Variance of group Y}$ (2)

F= 3.16

F(29, 30) at alpha 0.025 = 2.08

**Interpretation**

Because F>F(29,30) at alpha = 0.025, we can assume that the variances between the two groups are significantly different.

**Pooling the standard deviations**

$pooled SD\boldsymbol{=}\sqrt{\frac{{SD \left( Int \right)}^{2}+ {SD \left( Cont \right)}^{2}}{\boldsymbol{2}}}$ (4)

Pooled SD= 0.65

**Calculating of Cohen’s d**

d = $\frac{difference in mean outcome between groups}{standard deviation of outcome among participants}$ (6)

**d= 3.39**

**Calculating Glass delta**

$\Delta=\frac{difference in mean outcome between groups}{standard deviation of control group}$ (3)

Delta= 2.75

#### Unadjusted outcome: Anxiety 4 weeks after baseline

**Available data:**

intervention follow-up mean = 67.35

intervention follow-up SD = 8.11

control follow-up mean = 90.43

control follow-up SD = 7.76

Baseline intervention n = 31

baseline control n = 30

**Calculating the Variance**

Variance of intervention group = 65.77

Variance of control group= 60.22

**Calculating df numerator and df denominator**

df(numerator)= 30

df (denominator)= 29

**Conducting F-test**

$F=\frac{Variance group X (larger number)}{Variance of group Y}$ (2)

F= 1.09

F(30, 29) at alpha 0.025 = 2.09

**Interpretation**

Because F<F(30,29) at alpha = 0.025, we can assume that the variances between the two groups are not significantly different.

**Pooling the standard deviations**

$pooled SD\boldsymbol{=}\sqrt{\frac{{SD \left( Int \right)}^{2}+ {SD \left( Cont \right)}^{2}}{\boldsymbol{2}}}$ (4)

Pooled SD= 7.94

**Calculating of Cohen’s d**

d = $\frac{difference in mean outcome between groups}{standard deviation of outcome among participants}$ (6)

**d= -2.91**

#### Unadjusted outcome: Anxiety 5 weeks after baseline

**Available data:**

intervention follow-up mean = 85.25

intervention follow-up SD = 7.25

control follow-up mean = 76.9

control follow-up SD = 7.69

Baseline intervention n = 31

baseline control n = 30

**Calculating the Variance**

Variance of intervention group = 52.56

Variance of control group= 59.14

**Calculating df numerator and df denominator**

df(numerator)= 29

df (denominator)= 30

**Conducting F-test**

$F=\frac{Variance group X (larger number)}{Variance of group Y}$ (2)

F= 1.13

F(30, 29) at alpha 0.025 = 2.08

**Interpretation**

Because F<F(30,29) at alpha = 0.025, we can assume that the variances between the two groups are not significantly different.

**Pooling the standard deviations**

$pooled SD\boldsymbol{=}\sqrt{\frac{{SD \left( Int \right)}^{2}+ {SD \left( Cont \right)}^{2}}{\boldsymbol{2}}}$ (4)

Pooled SD= 7.47

**Calculating of Cohen’s d**

d = $\frac{difference in mean outcome between groups}{standard deviation of outcome among participants}$ (6)

**d= -2.5**

#### Unadjusted outcome: Anxiety 10 weeks after baseline

**Available data:**

intervention follow-up mean = 53.09

intervention follow-up SD = 3.11

control follow-up mean = 71.5

control follow-up SD = 7.61

Baseline intervention n = 31

baseline control n = 30

**Calculating the Variance**

Variance of intervention group = 9.67

Variance of control group= 57.91

**Calculating df numerator and df denominator**

df(numerator)= 29

df (denominator)= 30

**Conducting F-test**

$F=\frac{Variance group X (larger number)}{Variance of group Y}$ (2)

F= 5.99

F(30, 29) at alpha 0.025 = 2.09

**Interpretation**

Because F>F(30,29) at alpha = 0.025, we can assume that the variances between the two groups are significantly different.

**Pooling the standard deviations**

$pooled SD\boldsymbol{=}\sqrt{\frac{{SD \left( Int \right)}^{2}+ {SD \left( Cont \right)}^{2}}{\boldsymbol{2}}}$ (4)

Pooled SD= 5.81

**Calculating of Cohen’s d**

d = $\frac{difference in mean outcome between groups}{standard deviation of outcome among participants}$ (6)

**d= -3.17**

**Calculating Glass delta**

$\Delta=\frac{difference in mean outcome between groups}{standard deviation of control group}$ (3)

Delta= -2.42

#### Unadjusted outcome: Anxiety 16 weeks after baseline

**Available data:**

intervention follow-up mean = 50.74

intervention follow-up SD = 1.61

control follow-up mean = 62.6

control follow-up SD = 4.85

Baseline intervention n = 31

baseline control n = 30

**Calculating the Variance**

Variance of intervention group = 2.59

Variance of control group= 23.52

**Calculating df numerator and df denominator**

df(numerator)= 30

df (denominator)= 29

**Conducting F-test**

$F=\frac{Variance group X (larger number)}{Variance of group Y}$ (2)

F= 9.07

F(30, 29) at alpha 0.025 = 2.09

**Interpretation**

Because F>F(30,29) at alpha = 0.025, we can assume that the variances between the two groups are significantly different.

**Pooling the standard deviations**

$pooled SD\boldsymbol{=}\sqrt{\frac{{SD \left( Int \right)}^{2}+ {SD \left( Cont \right)}^{2}}{\boldsymbol{2}}}$ (4)

Pooled SD= 3.61

**Calculating of Cohen’s d**

d = $\frac{difference in mean outcome between groups}{standard deviation of outcome among participants}$ (6)

**d= -3.28**

**Calculating Glass delta**

$\Delta=\frac{difference in mean outcome between groups}{standard deviation of control group}$ (3)

Delta= -2.45

#### Unadjusted outcome: Depression 5 weeks after baseline

**Available data:**

intervention follow-up mean = 4.45

intervention follow-up SD = 2.91

control follow-up mean = 7.2

control follow-up SD = 1.79

Baseline intervention n = 31

baseline control n = 30

**Calculating the Variance**

Variance of intervention group = 8.47

Variance of control group= 3.2

**Calculating df numerator and df denominator**

df(numerator)= 30

df (denominator)= 29

**Conducting F-test**

$F=\frac{Variance group X (larger number)}{Variance of group Y}$ (2)

F= 2.64

F(30, 29) at alpha 0.025 = 2.09

**Interpretation**

Because F>F(30,29) at alpha = 0.025, we can assume that the variances between the two groups are significantly different.

**Pooling the standard deviations**

$pooled SD\boldsymbol{=}\sqrt{\frac{{SD \left( Int \right)}^{2}+ {SD \left( Cont \right)}^{2}}{\boldsymbol{2}}}$ (4)

Pooled SD= 2.42

**Calculating of Cohen’s d**

d = $\frac{difference in mean outcome between groups}{standard deviation of outcome among participants}$ (6)

**d= -1.14**

**Calculating Glass delta**

$\Delta=\frac{difference in mean outcome between groups}{standard deviation of control group}$ (3)

Delta= -1.54

#### Unadjusted outcome: Depression 10 weeks after baseline

**Available data:**

intervention follow-up mean = 0.81

intervention follow-up SD = 1.6

control follow-up mean = 3.47

control follow-up SD = 1.36

Baseline intervention n = 31

baseline control n = 30

**Calculating the Variance**

Variance of intervention group = 2.56

Variance of control group= 1.85

**Calculating df numerator and df denominator**

df(numerator)= 30

df (denominator)= 29

**Conducting F-test**

$F=\frac{Variance group X (larger number)}{Variance of group Y}$ (2)

F= 1.38

F(30, 29) at alpha 0.025 = 2.09

**Interpretation**

Because F<F(30,29) at alpha = 0.025, we can assume that the variances between the two groups are not significantly different.

**Pooling the standard deviations**

$pooled SD\boldsymbol{=}\sqrt{\frac{{SD \left( Int \right)}^{2}+ {SD \left( Cont \right)}^{2}}{\boldsymbol{2}}}$ (4)

Pooled SD= 1.48

**Calculating of Cohen’s d**

d = $\frac{difference in mean outcome between groups}{standard deviation of outcome among participants}$ (6)

**d= -1.79**

#### Unadjusted outcome: Depression 16 weeks after baseline

**Available data:**

intervention follow-up mean = 0.16

intervention follow-up SD = 0.58

control follow-up mean = 1.6

control follow-up SD = 0.93

Baseline intervention n = 31

baseline control n = 30

**Calculating the Variance**

Variance of intervention group = 0.34

Variance of control group= 0.86

**Calculating df numerator and df denominator**

df(numerator)= 30

df (denominator)= 29

**Conducting F-test**

$F=\frac{Variance group X (larger number)}{Variance of group Y}$ (2)

F= 2.57

F(30, 29) at alpha 0.025 = 2.09

**Interpretation**

Because F>F(30,29) at alpha = 0.025, we can assume that the variances between the two groups are significantly different.

**Pooling the standard deviations**

$pooled SD\boldsymbol{=}\sqrt{\frac{{SD \left( Int \right)}^{2}+ {SD \left( Cont \right)}^{2}}{\boldsymbol{2}}}$ (4)

Pooled SD= 0.78

**Calculating of Cohen’s d**

d = $\frac{difference in mean outcome between groups}{standard deviation of outcome among participants}$ (6)

**d= -1.86**

**Calculating Glass delta**

$\Delta=\frac{difference in mean outcome between groups}{standard deviation of control group}$ (3)

Delta= -1.55

### Study: Hong, 2023

#### Adjusted Outcome: Depression

Available data:

Unstandardized beta = -0.06

T-test for difference in mean change (from baseline to FU) between intervention and control = -0.056

Baseline intervention n = 21

Baseline control n = 23

$d=t \sqrt{\left( \frac{n\left( int \right)+n\left( cont \right)}{n\left( int \right) n\left( cont \right)} \right)\left( \frac{n\left( int \right)+n\left( cont \right)}{n\left( int \right)+n\left( cont \right)-2} \right)}$ (10)

d = -0.02

### Study: Hanita, 2022

#### Unadjusted outcome: Depression

**Available data:**

intervention follow-up mean = 3.9

intervention follow-up SD = 3.1

control follow-up mean = 8.2

control follow-up SD = 3.25

Baseline intervention n = 23

baseline control n = 22

**Calculating the Variance**

Variance of intervention group = 9.61

Variance of control group= 10.56

**Calculating df numerator and df denominator**

df(numerator)= 21

df (denominator)= 22

**Conducting F-test**

$F=\frac{Variance group X (larger number)}{Variance of group Y}$ (2)

F= 1.1

F(21, 22) at alpha 0.025 = 2.37

**Interpretation**

Because F<F(21,22) at alpha = 0.025, we can assume that the variances between the two groups are significantly different.

**Pooling the standard deviations**

$pooled SD\boldsymbol{=}\sqrt{\frac{{SD \left( Int \right)}^{2}+ {SD \left( Cont \right)}^{2}}{\boldsymbol{2}}}$ (4)

Pooled SD= 3.18

**Calculating of Cohen’s d**

d = $\frac{difference in mean outcome between groups}{standard deviation of outcome among participants}$ (6)

**d= -1.35**

#### Unadjusted outcome: Anxiety

**Available data:**

intervention follow-up mean = 7.3

intervention follow-up SD = 4.7

control follow-up mean = 8.1

control follow-up SD = 3.21

Baseline intervention n = 23

baseline control n = 22

**Calculating the Variance**

Variance of intervention group = 22.09

Variance of control group= 10.3

**Calculating df numerator and df denominator**

df(numerator)= 22

df (denominator)= 21

**Conducting F-test**

$F=\frac{Variance group X (larger number)}{Variance of group Y}$ (2)

F= 2.14

F(22, 21) at alpha 0.025 = 2.39

**Interpretation**

Because F<F(22,21) at alpha = 0.025, we can assume that the variances between the two groups are significantly different.

**Pooling the standard deviations**

$pooled SD\boldsymbol{=}\sqrt{\frac{{SD \left( Int \right)}^{2}+ {SD \left( Cont \right)}^{2}}{\boldsymbol{2}}}$ (4)

Pooled SD= 3.18

**Calculating of Cohen’s d**

d = $\frac{difference in mean outcome between groups}{standard deviation of outcome among participants}$ (6)

**d= -0.2**

### Study: Xu, 2021

#### Unadjusted outcome: drug positive test

**Available data:**

Mean of intervention group at follow-up= 3.3

Corresponding SD of intervention group at follow-up= 5

Mean of control group at follow-up = 7.5

SD of control at follow-up= 7.5

F-score = 4.358

P-value = .04

N intervention = 20

N control = 20

**testing assumption of equal variance**

Variance of intervention group = 25

Variance of control group = 56.25

**Calculating df numerator and df denominator**

df(numerator) = 19

df (denominator) =19

**Conducting F-test**

$F=\frac{Variance group X (larger number)}{Variance of group Y}$ (2)

F= 2.25

F(19, 19) at alpha 0.025 = 2.53

**Interpretation**

Because F<F (19,19) at alpha = 0.025, we can assume that the variances between the two groups are not significantly different.

**Pooling the standard deviations**

$pooled SD\boldsymbol{=}\sqrt{\frac{{SD \left( Int \right)}^{2}+ {SD \left( Cont \right)}^{2}}{\boldsymbol{2}}}$ (4)

Pooled SD= 6.37

**Mean difference between intervention and control group at FU**

Mean_dufference_= -4.2

**Calculation of Cohen’s d**

d = $\frac{difference in mean outcome between groups}{standard deviation of outcome among participants}$ (6)

**d= -0.66**

#### Unadjusted outcome: longest period of abstinence

**Available data:**

Intervention mean at FU = 24.65

Intervention SD at FU = 2.21

Control mean at FU = 22.8

Control SD at FU = 3.59

F-score = 3,8510

P-value = 0.06

N intervention = 20

N control = 20

**Testing assumption of equal variance**

Variance of intervention group = 4.8841

Variance of control group = 12.8881

**Calculating df numerator and df denominator**

df(numerator) = 19

df (denominator) =19

**Conducting F-test**

$F=\frac{Variance group X (larger number)}{Variance of group Y}$ (2)

F= 2.638787085

F(19, 19) at alpha 0.025 = 2.53

**Interpretation**

because F>F(19,19) at alpha = 0.025, we can assume that the variances between the two groups are significantly different.

**Mean difference between intervention and control group at follow-up**

Mean_difference_= 1.85

**Calculating Glass delta**

$\Delta=\frac{difference in mean outcome between groups}{standard deviation of control group}$ (3)

Delta= 0.52

**Calculating pooled SD**

$pooled SD\boldsymbol{=}\sqrt{\frac{{SD \left( Int \right)}^{2}+ {SD \left( Cont \right)}^{2}}{\boldsymbol{2}}}$ (4)

Pooled SD= 2.98

**Calculation of Cohen’s d**

d = $\frac{difference in mean outcome between groups}{standard deviation of outcome among participants}$ (6)

**d=0.62**

### Study: Rodriguez et al., 2021

#### Uadjusted outcome: depression severity

Available data:

intervention MIND + pre-post mean change score = -4.13

control MIND pre-post mean change score = -2.38

total sample SD change score = 5.29

Cohen‘s d = $\frac{difference in mean outcome between groups}{standard deviation of outcome among participants}$ (3)

**d=-0.33**

#### Unadjusted outcome: anxiety severity

Available data:

intervention MIND + pre-post mean change score = -2.91

control MIND pre-post mean change score = -2.62

total sample SD change score = 3.97

Cohen‘s d = $\frac{difference in mean outcome between groups}{standard deviation of outcome among participants}$ (3)

d=-0.07

#### Unadjusted outcome: indices of depression

Available data:

intervention MIND + pre-post mean change score = -2.87

control MIND pre-post mean change score = -2.62

total sample SD change score = 3.84

Cohen‘s d = $\frac{difference in mean outcome between groups}{standard deviation of outcome among participants}$ (3)

**d=-0.07**

#### Unadjusted outcome: indices of anxiety

Available data:

intervention MIND + pre-post mean change score = -1.65

control MIND pre-post mean change score = -1.29

total sample SD change score = 3.44

Cohen‘s d = $\frac{difference in mean outcome between groups}{standard deviation of outcome among participants}$ (3)

**d=-0.1**

#### Unadjusted outcome: indices of stress

Available data:

intervention MIND + pre-post mean change score = -2.34

control MIND pre-post mean change score = -2

total sample SD change score = 3.79

Cohen‘s d = $\frac{difference in mean outcome between groups}{standard deviation of outcome among participants}$ (3)

d=-0.09

#### Unadjusted outcome: mindfulness

Available data:

intervention MIND + pre-post mean change score = 3.59

control MIND pre-post mean change score = 6.04

total sample SD change score = 13.18

Cohen‘s d = $\frac{difference in mean outcome between groups}{standard deviation of outcome among participants}$ (3)

**d=-0.19**

### Study: Anttila, 2019 DepisNet Thai intervention group

#### Unadjusted outcome: severity of depression

**Available data:**

Baseline mean = 7.8

Baseline SD = 4.5

Follow-up mean = 7.5

Follow-up SD = 3.7

Mean change = 0.3

N baseline = 54

**Calculating SD change**

$SD\left( difference \right)= \sqrt{SD\left( int \right)+SD \left( cont \right)-2 r SD\left( int \right)SD(cont)}$ (6)

Under the assumption that r= 0.5

SD (difference)= 4.16

**Calculating the standard deviation within groups**

$SD\left( within \right)=\frac{SD(difference)}{\surd2(1-r)}$ (5)

Under the assumption that r= 0.5

SD(within)= 4.16

**Calculating the mean change**

Intervention at follow-up – intervention at baseline

Mean change= -0.3

**Calculation of Cohen’s d**

$d=\frac{difference in mean outcome between groups}{SD(within)}$ (5)

d= -0.07

#### Unadjusted outcome: severity of stress

**Available data:**

Baseline mean = 15.6

Baseline SD = 4.9

Follow-up mean = 16.3

Follow-up SD = 5.1

N baseline = 54

**Calculating SD change**

$SD\left( difference \right)= \sqrt{SD\left( int \right)+SD \left( cont \right)-2 r SD\left( int \right)SD(cont)}$ (6)

Under the assumption that r= 0.5

SD (difference)= 5

**Calculating the standard deviation within groups**

$SD\left( within \right)=\frac{SD(difference)}{\surd2(1-r)}$ (5)

Under the assumption that r= 0.5

SD(within)= 5

**Calculating the mean change**

Intervention at follow-up – intervention at baseline

Mean change= 0.7

**Calculation of Cohen’s d**

$d=\frac{difference in mean outcome between groups}{SD(within)}$ (5)

d= 0.14

### Study: Anttila, 2019 active control group

#### Unadjusted outcome: severity of depression

**Available data:**

Baseline mean = 7.8

Baseline SD = 3

Follow-up mean = 6.9

Follow-up SD = 3.5

N baseline = 55

**Calculating SD change**

$SD\left( difference \right)= \sqrt{SD\left( int \right)+SD \left( cont \right)-2 r SD\left( int \right)SD(cont)}$ (6)

Under the assumption that r= 0.5

SD (difference)= 3.28

**Calculating the standard deviation within groups**

$SD\left( within \right)=\frac{SD(difference)}{\surd2(1-r)}$ (5)

Under the assumption that r= 0.5

SD(within)= 3.28

**Calculating the mean change**

Intervention at follow-up – intervention at baseline

Mean change= -0.9

**Calculation of Cohen’s d**

$d=\frac{difference in mean outcome between groups}{SD(within)}$ (5)

d= -0.27

#### Unadjusted outcome: severity of stress

**Available data:**

Baseline mean = 15.9

Baseline SD = 4.3

Follow-up mean = 15.5

Follow-up SD = 4.8

N baseline = 55

**Calculating SD change**

$SD\left( difference \right)= \sqrt{SD\left( int \right)+SD \left( cont \right)-2 r SD\left( int \right)SD(cont)}$ (6)

Under the assumption that r= 0.5

SD (difference)= 4.57

**Calculating the standard deviation within groups**

$SD\left( within \right)=\frac{SD(difference)}{\surd2(1-r)}$ (5)

Under the assumption that r= 0.5

SD(within)= 4.57

**Calculating the mean change**

Intervention at follow-up – intervention at baseline

Mean change= -0.4

**Calculation of Cohen’s d**

$d=\frac{difference in mean outcome between groups}{SD(within)}$ (5)

d= -0.09

### Study: Menezes, 2019 Brazil and Peru (2 trials)

#### Outcome: proportion of people with disability at baseline who had no disability at follow-up

**Available data:**

Brazil setting:

Number of people with disability (yes/no) at baseline= 20

Number of people without disability at follow-up = 18

Peru 1 setting:

Number of people with disability (yes/no) at baseline = 15

Number of people without disability at follow-up = 14

Peru 2 setting:

Number of people with disability (yes/no) at baseline = 16

Number of people without disability at follow-up = 16

**Calculating proportion of people without disability at follow-up in each setting**

Brazil= 10%

Peru 1= 6.67%

Peru 2 = 0%

### Study: Zhou, 2019

#### Adjusted outcome: level of resilience

**Available data:**

Mean of intervention group at baseline = 57.69

SD of intervention group at baseline = 9.87

Mean of control group at baseline = 58.2

SD of control group at baseline = 10.01

Mean of intervention group at follow-up= 90.01

SD of intervention at follow-up = 10.10

Mean of control group at follow-up = 63.14

SD of control group at follow-up = 9.97

Adjusted mean difference = 26.87

Corresponding 95% CI lower limit = 7.2

Corresponding 95% CI upper limit = 46.54

N intervention baseline = 66

N control baseline = 66

**Testing assumption of equal variances**

Variance of intervention group= 97.42

Variance of control group = 100.2

**Calculating df numerator and df denominator**

df(numerator) = 65

df (denominator) =65

**Conducting F-test**

$F=\frac{Variance group X (larger number)}{Variance of group Y}$ (2)

F= 1.02856999

F (65, 65) at alpha 0.025 = 1.63

**Interpretation**

Because F<F(65,65) at alpha = 0.025, we can assume that the variances between the two groups are not significantly different.

**Pooling the standard deviations**

$pooled SD\boldsymbol{=}\sqrt{\frac{{SD \left( Int \right)}^{2}+ {SD \left( Cont \right)}^{2}}{\boldsymbol{2}}}$ (4)

Pooled SD= 10.04

**Calculation of Cohen’s d**

d = $\frac{difference in mean outcome between groups}{standard deviation of outcome among participants}$ (6)

**d= 2.68**

### Study: Gonsalves, 2021

#### Unadjusted outcome: psychosocial problem severity at 2-3 weeks post-intervention

Available data:

Mean at baseline = 6.63

SD at baseline = 2.07

Mean at follow-up = 3.27

SD at follow-up = 1.72

mean difference = -3.36

**Calculating SD change**

$SD\left( difference \right)= \sqrt{SD\left( int \right)+SD \left( cont \right)-2 r SD\left( int \right)SD(cont)}$ (6)

Under the assumption that r= 0.5

SD(difference)= 1.92

**Calculating the standard deviation within groups**

$SD\left( within \right)=\frac{SD(difference)}{\surd2(1-r)}$ (5)

Under the assumption that r= 0.5

SD(within)= 1.92

**Calculation the mean change score between FU and baseline**

Mean_change_= -3.36

**Calculation of Cohen’s d**

$d=\frac{difference in mean outcome between groups}{SD(within)}$ (5)

d= -1,75

#### Unadjusted outcome: Mental health symptoms at 2-3 weeks post-intervention

Available data:

Mean at baseline = 15.71

SD at baseline = 5.56

Mean at follow-up = 13.23

SD at follow-up = 5.4

**Calculating SD change**

$SD\left( difference \right)= \sqrt{SD\left( int \right)+SD \left( cont \right)-2 r SD\left( int \right)SD(cont)}$ (6)

Under the assumption that r= 0.5

SD(difference)= 5.48

**Calculating the standard deviation within groups**

$SD\left( within \right)=\frac{SD(difference)}{\surd2(1-r)}$ (5)

Under the assumption that r= 0.5

SD(within)= 5.48

**Calculation the mean change score between FU and baseline**

Mean_change_= -2.48

**Calculation of Cohen’s d**

$d=\frac{difference in mean outcome between groups}{SD(within)}$ (5)

d= -0.45

#### Unadjusted outcome: Stress level at 2-3 weeks post-intervention

Available data:

Mean at baseline = 7.62

SD at baseline = 2.66

Mean at follow-up = 6.32

SD at follow-up =2.47

**Calculating SD change**

$SD\left( difference \right)= \sqrt{SD\left( int \right)+SD \left( cont \right)-2 r SD\left( int \right)SD(cont)}$ (6)

Under the assumption that r= 0.5

SD(difference)= 2.57

**Calculating the standard deviation within groups**

$SD\left( within \right)=\frac{SD(difference)}{\surd2(1-r)}$ (5)

Under the assumption that r= 0.5

SD(within)= 2,57

**Calculation the mean change score between FU and baseline**

Mean_change_= -1,3

**Calculation of Cohen’s d**

$d=\frac{difference in mean outcome between groups}{SD(within)}$ (5)

d= -0.51

#### Unadjusted outcome: Mental wellbeing at 2-3 weeks post-intervention

Available data:

Mean at baseline = 22.21

SD at baseline = 4.35

Mean at follow-up = 23.8

SD at follow-up =4.48

**Calculating SD change**

$SD\left( difference \right)= \sqrt{SD\left( int \right)+SD \left( cont \right)-2 r SD\left( int \right)SD(cont)}$ (6)

Under the assumption that r= 0.5

SD(difference)= 22.22

**Calculating the standard deviation within groups**

$SD\left( within \right)=\frac{SD(difference)}{\surd2(1-r)}$ (5)

Under the assumption that r= 0.5

SD(within)= 22.22

**Calculation the mean change score between FU and baseline**

Mean_change_= 1.59

**Calculation of Cohen’s d**

$d=\frac{difference in mean outcome between groups}{SD(within)}$ (5)

**d= 0.07**

#### Unadjusted outcome: psychosocial problem severity at 9-10 weeks post-intervention

Available data:

Mean at baseline = 6.62

SD at baseline = 2.08

Mean at follow-up = 2.93

SD at follow-up = 1.65

**Calculating SD change**

$SD\left( difference \right)= \sqrt{SD\left( int \right)+SD \left( cont \right)-2 r SD\left( int \right)SD(cont)}$ (6)

Under the assumption that r= 0.5

SD(difference)= 1.9

**Calculating the standard deviation within groups**

$SD\left( within \right)=\frac{SD(difference)}{\surd2(1-r)}$ (5)

Under the assumption that r= 0.5

SD(within)_=_ 1.9

**Calculation the mean change score between FU and baseline**

Mean_change_= -3.36

**Calculation of Cohen’s d**

$d=\frac{difference in mean outcome between groups}{SD(within)}$ (5)

d= -1.75

#### Unadjusted outcome: Mental health symptoms at 9-10 weeks post-intervention

Available data:

Mean at baseline = 15.93

SD at baseline = 5.42

Mean at follow-up = 12.6

SD at follow-up = 5.65

**Calculating SD change**

$SD\left( difference \right)= \sqrt{SD\left( int \right)+SD \left( cont \right)-2 r SD\left( int \right)SD(cont)}$ (6)

Under the assumption that r= 0.5

SD(difference)= 5.54

**Calculating the standard deviation within groups**

$SD\left( within \right)=\frac{SD(difference)}{\surd2(1-r)}$ (5)

Under the assumption that r= 0.5

SD(within)= 5.54

**Calculation the mean change score between FU and baseline**

Mean_change_= -3.33

**Calculation of Cohen’s d**

$d=\frac{difference in mean outcome between groups}{SD(within)}$ (5)

d= -0.6

#### Unadjusted outcome: Stress level at 9-10 weeks post-intervention

Available data:

Mean at baseline = 7.66

SD at baseline = 2.41

Mean at follow-up = 6.19

SD at follow-up =2.52

**Calculating SD change**

$SD\left( difference \right)= \sqrt{SD\left( int \right)+SD \left( cont \right)-2 r SD\left( int \right)SD(cont)}$ (6)

Under the assumption that r= 0.5

SD(difference)= 2.47

**Calculating the standard deviation within groups**

$SD\left( within \right)=\frac{SD(difference)}{\surd2(1-r)}$ (5)

Under the assumption that r= 0.5

SD(within)= 2.47

**Calculation the mean change score between FU and baseline**

Mean_change_= -1.47

**Calculation of Cohen’s d**

$d=\frac{difference in mean outcome between groups}{SD(within)}$ (5)

d= -0.6

#### Unadjusted outcome: Mental wellbeing at 9-10 weeks post-intervention

Available data:

Mean at baseline = 21.74

SD at baseline = 4.06

Mean at follow-up = 23.23

SD at follow-up = 5.02

**Calculating SD change**

$SD\left( difference \right)= \sqrt{SD\left( int \right)+SD \left( cont \right)-2 r SD\left( int \right)SD(cont)}$ (6)

Under the assumption that r= 0.5

SD(difference= 4.62

**Calculating the standard deviation within groups**

$SD\left( within \right)=\frac{SD(difference)}{\surd2(1-r)}$ (5)

Under the assumption that r= 0.5

SD(within)= 4.62

**Calculation the mean change score between FU and baseline**

Mean_change_= 1.49

**Calculation of Cohen’s d**

$d=\frac{difference in mean outcome between groups}{SD(within)}$ (5)

**d= 0.32**

### Study: Arjadi et al., 2019

#### Adjusted outcome: Severity of depression

Available data:

Mean of intervention group at follow-up = 8.5

SD of intervention group at follow-up = 5.75

Mean of control group at follow-up = 10.83

SD of control group at follow-up = 6.21

**Pooling the standard deviations**

$pooled SD\boldsymbol{=}\sqrt{\frac{{SD \left( Int \right)}^{2}+ {SD \left( Cont \right)}^{2}}{\boldsymbol{2}}}$ (4)

Pooled SD= 5.98

**Calculation of Cohen’s d**

d = $\frac{difference in mean outcome between groups}{standard deviation of outcome among participants}$ (6)

d= -0.39

### Study: Araya, 2020 Brazil setting

#### Adjusted outcome: Severity of depression

**Available data:**

Proportion with outcome in intervention group at 3 months = 159

Sample size intervention group at 3 months = 391

Proportion with outcome in control group at 3 months = 114

Sample size control group at 3 months = 399

Adjusted Odds ratio = 1.6

Corresponding 95% CI lower limit = 1.2

Corresponding 95% CI upper limit = 2.2

**Converting the unadjusted OR to Cohen’s d**

$d=LogOddsRatio\frac{\surd3}{\pi}$ (5)

d = 0.11

### Study: Araya, 2020 Peru setting

#### Adjusted outcome: Severity of depression

**Available data:**

Proportion with outcome in intervention group at 3 months = 108

Sample size intervention group at 3 months = 205

Proportion with outcome in control group at 3 months = 70

Sample size control group at 3 months = 205

Adjusted Odds ratio = 2.1

Corresponding 95% CI lower limit = 1.4

Corresponding 95% CI upper limit = 3.2

**Converting the unadjusted OR to Cohen’s d**

$d=LogOddsRatio\frac{\surd3}{\pi}$ (5)

d = 0.18

### Study: Khan et al. 2019

#### Adjusted outcome: Psychological distress in terms of states of anxiety and depression

**Available data:**

Mean of intervention group at baseline = 20.15

SD of intervention group at baseline = 7.13

Mean of intervention mean at follow-up = 15.7

SD of intervention group at follow-up = 9.04

Mean of control group at baseline = 24.15

SD of control group at baseline = 7.58

Mean of control group at follow-up = 23.21

SD of control at follow-up = 8.32

Adjusted mean difference between groups at follow-up = -4.65

Corresponding 95%CI lower limit = -7.35

Corresponding 95%CI upper limit = -1.95

N intervention group baseline = 59

N intervention group FU = 54

N control group baseline = 60

N control group FU = 58

**Testing assumption of equal variances**

Variance of intervention group= 50.84

Variance of control group= 57.46

**Calculating df numerator and df denominator**

df(numerator) = 59

df (denominator) =57

**Conducting F-test**

$F=\frac{Variance group X (larger number)}{Variance of group Y}$ (2)

F= 1.13

F(59, 57) at alpha 0.025 = 1.68

**Interpretation**

Because F<F(59,57) at alpha = 0.025, we can assume that the variances between the two groups are not significantly different.

**Calculating the standard error based on 95% CI**

$SE=\frac{(95\%CI upper limit-lower limit)}{3.92}$ (6)

SE= 1.38

**Calculation of the pooled standard deviation**

$SD=\frac{SE}{\sqrt{\frac{1}{N\left( int \right)}+\frac{1}{N(cont)}}}$ (6)

SD= 7.28

**Calculation of Cohen’s d**

d = $\frac{difference in mean outcome between groups}{standard deviation of outcome among participants}$ (6)

d = -0.62

*Adjusted outcome: Psychological distress in terms of anxiety*

Available data:

Intervention mean baseline = 10.81

Intervention SD baseline = 3.68

Intervention mean FU = 7.59

Intervention SD FU = 4.66

Control mean baseline = 13.13

Control SD baseline = 3.94

Control mean FU = 11.52

Control SD FU = 4.59

Adjusted mean difference between groups = -2.62

Corresponding 95%CI lower limit = -4.37

Corresponding 95%CI upper limit = -0.86

N intervention group baseline = 59

N intervention group FU = 54

N control group baseline = 60

N control group FU = 58

**Testing assumption of equal variances**

Variance of intervention group= 13.5424

Variance of control group= 15.5236

**Calculating df numerator and df denominator**

df(numerator) = 59

df (denominator) =57

**Conducting F-test**

$F=\frac{Variance group X (larger number)}{Variance of group Y}$ (2)

F= 1.146296078

F(59, 57) at alpha 0.025 = 1.678891

**Interpretation**

Because F<F(59,57) at alpha = 0.025, we can assume that the variances between the two groups are not significantly different.

**Calculating standard error based on 95% CI**

$SE=\frac{(95\%CI upper limit-lower limit)}{3.92}$ (6)

SE= 0.89540816

**Calculation of SD**

$SD=\frac{SE}{\sqrt{\frac{1}{N\left( int \right)}+\frac{1}{N(cont)}}}$ (6)

SD= 4.883702495

**Calculation of Cohen’s d**

d = $\frac{difference in mean outcome between groups}{standard deviation of outcome among participants}$ (6)

d= -0.54

*Adjusted outcome: Psychological distress in terms of depression*

**Available data:**

Intervention mean baseline = 9.34

Intervention SD baseline = 4.38

Intervention mean FU = 8.11

Intervention SD FU = 5.13

Control mean baseline = 11.02

Control SD baseline = 4.32

Control mean FU = 11.69

Control SD FU = 4.42

Adjusted mean difference between groups = -2.48

Corresponding 95%CI lower limit = -4

Corresponding 95%CI upper limit = -0.96

N intervention group baseline = 59

N intervention group FU = 54

N control group baseline = 60

N control group FU = 58

**Testing the assumption of equal variances**

Variance of intervention group= 19.1844

Variance of control group= 18.6624

**Calculating df numerator and df denominator**

df(numerator) = 57

df (denominator) =59

**Conducting F-test**

$F=\frac{Variance group X (larger number)}{Variance of group Y}$ (2)

F= 1.027970679

F(57, 59) at alpha 0.025 = 1.6769

**Interpretation**

Because F<F(59,57) at alpha = 0.025, we can assume that the variances between the two groups are not significantly different.

**Calculating standard error based on 95% CI**

$SE=\frac{(95\%CI upper limit-lower limit)}{3.92}$ (6)

SE= 0.7755102

**Calculation of standard deviation**

$SD=\frac{SE}{\sqrt{\frac{1}{N\left( int \right)}+\frac{1}{N(cont)}}}$ (6)

SD= 4.194059509

**Calculation of Cohen’s d**

d = $\frac{difference in mean outcome between groups}{standard deviation of outcome among participants}$ (6)

d= -0.6

*Adjusted Outcome: functional disability*

**Available data:**

Intervention mean baseline = 30.24

Intervention SD baseline = 6.62

Intervention mean FU = 24.44

Intervention SD FU = 8.9

Control mean baseline = 31.92

Control SD baseline = 7.2

Control mean FU = 30.86

Control SD FU = 8.64

Adjusted mean difference between groups = -5.37

Corresponding 95%CI lower limit = -8.97

Corresponding 95%CI upper limit = -1.76

N intervention group baseline = 59

N intervention group FU = 54

N control group baseline = 60

N control group FU = 58

**Testing the assumption of equal variance**

Variance of intervention group= 43.8244

Variance of control group= 51.84

**Calculating df numerator and df denominator**

df(numerator) = 59

df (denominator) =57

**Conducting F-test**

$F=\frac{Variance group X (larger number)}{Variance of group Y}$ (2)

F= 1.182902675

F(57, 59) at alpha 0.025 = 1.67889

**Interpretation**

Because F<F(59,57) at alpha = 0.025, we can assume that the variances between the two groups are not significantly different.

**Calculating standard error based on 95% CI**

$SE=\frac{(95\%CI upper limit-lower limit)}{3.92}$ (6)

SE= 1.839285714

**Calculation of standard deviation**

$SD=\frac{SE}{\sqrt{\frac{1}{N\left( int \right)}+\frac{1}{N(cont)}}}$ (6)

SD= 10.03176495

**Calculating Cohen’s d**

d = $\frac{difference in mean outcome between groups}{standard deviation of outcome among participants}$ (6)

d = -0.53

*Adjusted outcome: psychological outcome profile*

**Available data:**

Intervention mean baseline = 14.69

Intervention SD baseline = 3.31

Intervention mean FU = 9.22

Intervention SD FU = 4.97

Control mean baseline = 15.63

Control SD baseline = 3.39

Control mean FU = 14.21

Control SD FU = 3.98

Adjusted mean difference between groups = -4.49

Corresponding 95%CI lower limit = -6.41

Corresponding 95%CI upper limit = -2.58

N intervention group baseline = 55

N intervention group FU = 54

N control group baseline = 60

N control group FU = 57

**Testing assumption of equal variances at baseline**

Variance of intervention group= 10.9561

Variance of control group= 11.4921

**Calculating df numerator and df denominator**

df(numerator) = 59

df (denominator) =54

**Conducting F-test**

$F=\frac{Variance group X (larger number)}{Variance of group Y}$ (2)

F= 1.048922518

F(59, 54) at alpha 0.025 = 1.69968

**Interpretation**

Because F<F(59,54) at alpha = 0.025, we can assume that the variances between the two groups are not significantly different.

**Calculating standard error based on 95% CI**

$SE=\frac{(95\%CI upper limit-lower limit)}{3.92}$ (6)

SE= 0.97704082

**Calculation of the standard deviation**

$SD=\frac{SE}{\sqrt{\frac{1}{N\left( int \right)}+\frac{1}{N(cont)}}}$ (6)

SD= 5.233843793

**Calculating Cohen’s d**

d = $\frac{difference in mean outcome between groups}{standard deviation of outcome among participants}$ (6)

d = -0.86

*Adjusted outcome: PTSD symptoms*

**Available data:**

Intervention mean baseline = 28.47

Intervention SD baseline = 15.8

Intervention mean FU = 17.65

Intervention SD FU = 15.59

Control mean baseline = 36.53

Control SD baseline = 16.73

Control mean FU = 24.02

Control SD FU = 16.26

Adjusted mean difference between groups = -2.79

Corresponding 95%CI lower limit = -9.51

Corresponding 95%CI upper limit = 3.94

N intervention group baseline = 59

N intervention group FU = 54

N control group baseline = 60

N control group FU = 58

**Testing assumption of equal variances at baseline**

Variance of intervention group= 249.64

Variance of control group= 279.8929

**Calculating df numerator and df denominator**

df(numerator) = 59

df (denominator) =58

**Conducting F-test**

$F=\frac{Variance group X (larger number)}{Variance of group Y}$ (2)

F= 1.121186108

F(59, 58) at alpha 0.025 = 1.67889

**Interpretation**

Because F<F(59,57) at alpha = 0.025, we can assume that the variances between the two groups are not significantly different.

**Calculating standard error based on 95% CI**

$SE=\frac{(95\%CI upper limit-lower limit)}{3.92}$ (6)

SE= 3.43112245

**Calculation of the pooled standard deviation**

$SD=\frac{SE}{\sqrt{\frac{1}{N\left( int \right)}+\frac{1}{N(cont)}}}$ (6)

SD= 18.71390272

**Calculating Cohen’s d**

d = $\frac{difference in mean outcome between groups}{standard deviation of outcome among participants}$ (6)

d= -0.15

*Adjusted outcome: generalised distress*

**Available data:**

Intervention mean baseline = 11.42

Intervention SD baseline = 5.71

Intervention mean FU = 10.06

Intervention SD FU = 6.45

Control mean baseline = 13.23

Control SD baseline = 6.92

Control mean FU = 12.05

Control SD FU = 6.09

Adjusted mean difference between groups = -1.06

Corresponding 95%CI lower limit = -3.59

Corresponding 95%CI upper limit = 1.48

N intervention group baseline = 59

N intervention group FU = 54

N control group baseline = 60

N control group FU = 58

**Testing assumption of equal variances at baseline**

Variance of intervention group= 32.6041

Variance of control group= 47.8864

**Calculating df numerator and df denominator**

df(numerator) = 59

df (denominator) =58

**Conducting F-test**

$F=\frac{Variance group X (larger number)}{Variance of group Y}$ (2)

F= 1.468723259

F(59, 58) at alpha 0.025 = 1.67889

**Interpretation**

Because F<F(59,58) at alpha = 0.025, we can assume that the variances between the two groups are not significantly different.

**Calculating standard error based on 95% CI**

$SE=\frac{(95\%CI upper limit-lower limit)}{3.92}$ (6)

SE= 1.29336735

**Calculation of the pooled standard deviation**

$SD=\frac{SE}{\sqrt{\frac{1}{N\left( int \right)}+\frac{1}{N(cont)}}}$ (6)

SD= 7.054236937

**Calculating Cohen’s d**

d = $\frac{difference in mean outcome between groups}{standard deviation of outcome among participants}$ (6)

d= -0.15

### Study: Rhaman et al., 2019

#### Adjusted outcome: psychological distress depression and anxiety at 1 week post baseline

Available data:

Intervention group mean at follow-up = 10.58

SD of intervention group at follow-up = 8.05

Control group mean at follow-up = 17

SD of control group at follow-up = 8.3

intervention sample size at baseline = 306

control sample size at baseline = 305

**Pooling the standard deviations**

$pooled SD\boldsymbol{=}\sqrt{\frac{{SD \left( Int \right)}^{2}+ {SD \left( Cont \right)}^{2}}{\boldsymbol{2}}}$ (4)

Pooled SD= 8.18

**Calculation of Cohen’s d**

d = $\frac{difference in mean outcome between groups}{standard deviation of outcome among participants}$ (6)

d= -0.79

#### Adjusted outcome: psychological distress depression and anxiety at 1 week post baseline

Available data:

Intervention group mean at follow-up = 10.01

SD of intervention group at follow-up = 7.54

Control group mean at follow-up = 14.75

SD of control group at follow-up = 8.3

intervention sample size at baseline = 306

control sample size at baseline = 305

**Pooling the standard deviations**

$pooled SD\boldsymbol{=}\sqrt{\frac{{SD \left( Int \right)}^{2}+ {SD \left( Cont \right)}^{2}}{\boldsymbol{2}}}$ (4)

Pooled SD= 7.83

**Calculation of Cohen’s d**

d = $\frac{difference in mean outcome between groups}{standard deviation of outcome among participants}$ (6)

d= -0.6

### Study: Chen, 2022

#### Unadjusted outcome: depression at 3 months

**Available data:**

Mean of intervention group at follow-up = 17.97

SD of intervention group at follow-up = 5.74

Mean of control group at follow-up = 19.51

SD of control group at follow-up = 4.87

Observed mean difference = -1.54

Adjusted estimated mean difference = -2.07

95% CI = -3.36 , -0.78

Baseline intervention n = 1232

Baseline control n = 1133

**Calculating the Variance**

Variance of intervention group = 32.95

Variance of control group= 23.72

**Calculating df numerator and df denominator**

df(numerator)= 1231

df (denominator)= 1132

**Conducting F-test**

$F=\frac{Variance group X (larger number)}{Variance of group Y}$ (2)

F= 1.39

F(1231, 1132) at alpha 0.025 = 1.12

**Interpretation**

Because F>F(1231,1132) at alpha = 0.025, we can assume that the variances between the two groups are significantly different.

**Pooling the standard deviations**

$pooled SD\boldsymbol{=}\sqrt{\frac{{SD \left( Int \right)}^{2}+ {SD \left( Cont \right)}^{2}}{\boldsymbol{2}}}$ (4)

Pooled SD= 3.44

**Calculating of Cohen’s d**

d = $\frac{difference in mean outcome between groups}{standard deviation of outcome among participants}$ (6)

**d= -0.45**

**Calculating Glass delta**

$\Delta=\frac{difference in mean outcome between groups}{standard deviation of control group}$ (3)

Delta= -0.32

#### Adjusted outcome: depression at 3 months

**Calculating the standard error based on 95% CI**

$SE=\frac{(95\%CI upper limit-lower limit)}{3.92}$ (6)

SE=0.66

**Calculating the unadjusted within-group standard deviation**

$SD\boldsymbol{=}\frac{SE}{\sqrt{\frac{1}{N\left( int \right)}+\frac{1}{N(cont)}}}$ (6)

SD= 15.99

**Calculating of Cohen’s**

d = $\frac{difference in mean outcome between groups}{standard deviation of outcome among participants}$ (6)

d= -0.13

#### Unadjusted outcome: depression at 6 months

Available data:

Mean of intervention group at follow-up = 15.63

SD of intervention group at follow-up = 5.02

Mean of control group at follow-up = 19.58

SD of control group at follow-up = 4.64

Observed mean difference = -3.95

Adjusted estimated mean difference = -4.05

95% CI = -5.79 , -3.2

Baseline intervention n = 1232

Baseline control n = 1133

**Calculating the Variance**

Variance of intervention group = 25.2

Variance of control group= 21.53

**Calculating df numerator and df denominator**

df(numerator)= 1231

df (denominator)= 1132

**Conducting F-test**

$F=\frac{Variance group X (larger number)}{Variance of group Y}$ (2)

F= 1.17

F(1231, 1132) at alpha 0.025 = 1.12

**Interpretation**

Because F>F(1231,1132) at alpha = 0.025, we can assume that the variances between the two groups are significantly different.

**Pooling the standard deviations**

$pooled SD\boldsymbol{=}\sqrt{\frac{{SD \left( Int \right)}^{2}+ {SD \left( Cont \right)}^{2}}{\boldsymbol{2}}}$ (4)

Pooled SD= 4.83

**Calculating of Cohen’s d**

d = $\frac{difference in mean outcome between groups}{standard deviation of outcome among participants}$ (6)

**d= -0.82**

**Calculating Glass delta**

$\Delta=\frac{difference in mean outcome between groups}{standard deviation of control group}$ (3)

Delta= -0.85

#### Adjusted outcome: depression at 6 months

**Calculating the standard error based on 95% CI**

$SE=\frac{(95\%CI upper limit-lower limit)}{3.92}$ (6)

SE=0.66

**Calculating the unadjusted within-group standard deviation**

$SD\boldsymbol{=}\frac{SE}{\sqrt{\frac{1}{N\left( int \right)}+\frac{1}{N(cont)}}}$ (6)

SD= 16.05

**Calculating of Cohen’s**

d = $\frac{difference in mean outcome between groups}{standard deviation of outcome among participants}$ (6)

d= -0.25

#### Unadjusted outcome: depression at 9 months

**Available data:**

Mean of intervention group at follow-up = 13.77

SD of intervention group at follow-up = 4.73

Mean of control group at follow-up = 18.85

SD of control group at follow-up = 4.61

Observed mean difference = -5.08

Adjusted estimated mean difference = -5.71

95% CI = -7.1 , -4.41

Baseline intervention n = 1232

Baseline control n = 1133

**Calculating the Variance**

Variance of intervention group = 22.37

Variance of control group= 21.25

**Calculating df numerator and df denominator**

df(numerator)= 1231

df (denominator)= 1132

**Conducting F-test**

$F=\frac{Variance group X (larger number)}{Variance of group Y}$ (2)

F= 1.05

F(1231, 1132) at alpha 0.025 = 1.12

**Interpretation**

Because F<F(1231,1132) at alpha = 0.025, we can assume that the variances between the two groups are not significantly different.

**Pooling the standard deviations**

$pooled SD\boldsymbol{=}\sqrt{\frac{{SD \left( Int \right)}^{2}+ {SD \left( Cont \right)}^{2}}{\boldsymbol{2}}}$ (4)

Pooled SD= 4.67

**Calculating of Cohen’s d**

d = $\frac{difference in mean outcome between groups}{standard deviation of outcome among participants}$ (6)

**d= -1.09**

#### Adjusted outcome: depression at 9 months

**Calculating the standard error based on 95% CI**

$SE=\frac{(95\%CI upper limit-lower limit)}{3.92}$ (6)

SE=0.69

**Calculating the unadjusted within-group standard deviation**

$SD\boldsymbol{=}\frac{SE}{\sqrt{\frac{1}{N\left( int \right)}+\frac{1}{N(cont)}}}$ (6)

SD= 16.67

**Calculating of Cohen’s**

d = $\frac{difference in mean outcome between groups}{standard deviation of outcome among participants}$ (6)

d= -0.34

#### Unadjusted outcome: depression at 12 months

Available data:

Mean of intervention group at follow-up = 12.69

SD of intervention group at follow-up = 4.22

Mean of control group at follow-up = 18.77

SD of control group at follow-up = 4.67

Observed mean difference = -6.08

Adjusted estimated mean difference = -6.67

95% CI = -7.97 , -5.37

Baseline intervention n = 1232

Baseline control n = 1133

**Calculating the Variance**

Variance of intervention group = 17.8

Variance of control group= 21.8

**Calculating df numerator and df denominator**

df(numerator)= 1132

df (denominator)= 1231

**Conducting F-test**

$F=\frac{Variance group X (larger number)}{Variance of group Y}$ (2)

F= 1.22

F(1231, 1132) at alpha 0.025 = 1.12

**Interpretation**

Because F>F(1231,1132) at alpha = 0.025, we can assume that the variances between the two groups are not significantly different.

**Pooling the standard deviations**

$pooled SD\boldsymbol{=}\sqrt{\frac{{SD \left( Int \right)}^{2}+ {SD \left( Cont \right)}^{2}}{\boldsymbol{2}}}$ (4)

Pooled SD= 4.45

**Calculating of Cohen’s d**

d = $\frac{difference in mean outcome between groups}{standard deviation of outcome among participants}$ (6)

**d= -1.37**

**Calculating Glass delta**

$\Delta=\frac{difference in mean outcome between groups}{standard deviation of control group}$ (3)

Delta= -1.3

#### Adjusted outcome: depression at 12 months

**Calculating the standard error based on 95% CI**

$SE=\frac{(95\%CI upper limit-lower limit)}{3.92}$ (6)

SE=0.66

**Calculating the unadjusted within-group standard deviation**

$SD\boldsymbol{=}\frac{SE}{\sqrt{\frac{1}{N\left( int \right)}+\frac{1}{N(cont)}}}$ (6)

SD= 16.11

**Calculating of Cohen’s**

d = $\frac{difference in mean outcome between groups}{standard deviation of outcome among participants}$ (6)

**d= -0.41**

# Overview of statistical parameters

| **Study** | **Observed vs. estimated outcomes used for effect size calculation** | **Variables adjusted for** | **Variables used to calculate Cohen’s d** |
| --- | --- | --- | --- |
| Rahman (11) | Observed |  | Mean and SD for two groups at FU |
| Muke (12) | Observed |  | Mean and SD for one group at baseline and FU, fixed r=0.5 |
| Nisar (13) | Estimated | Demographics, work experience, prior mental health training, knowledge of perinatal depression | Unadjusted outcome: Mean for two groups and pooled SD at FU; Adjusted: Adjusted mean difference of both groups at FU, corresponding 95% CI |
| Pereira (14) | Estimated | Clusters: Schools | Adjusted regression coefficient for difference in outcome between two groups at FU, corresponding robust standard error |
| Maulik (15) | Observed |  | Proportion of outcome at baseline and FU in one group |
| Maulik (16) | Estimated | Clusters: villages, marital status | Odds ratio |
| Doukani (17) | Observed |  | Mean and SD for one group at baseline and FU, fixed r=0.5 |
| Dambi (18) | Observed |  | Mean and SD for one group at baseline and FU, fixed r=0.5 |
| Chibanda (19) | Estimated | HIV status, demographics, and baseline common mental disorder score | Unadjusted: Mean for two group and corresponding 95% CI at FU; Adjusted: adjusted mean difference and corresponding 95% CI of both groups at FU |
| Ross (20) | Estimated |  | Mean and SD for two groups at FU |
| Ebrahem (21) | Observed |  | Proportion of outcome at baseline and FU in one group |
| Scazufca(22) | Observed |  | Mean and SD for two groups at FU |
| Garg (23) | Observed |  | Mean and SD for one group at baseline and FU, fixed r=0.5 |
| Liu (24) | Observed |  | Mean change and SD change from baseline to FU of two groups separately |
| Öztoprak (25) | Observed |  | Mean and SD of two groups at FU |
| Hong (26) | Estimated |  | t-test, baseline n for intervention and control |
| Hanita (27) | Observed |  | Mean and SD for two groups at FU |
| Xu (28) | Observed |  | Mean and SD for two groups at FU |
| Rodrgiuez (29) | Estimated |  | Mean change from baseline to FU of two groups separately and total sample SD change |
| Anttila (30) | Observed |  | Mean, SD for one group at baseline and FU, fixed r=0.5 |
| Menezes (31) | Observed |  | Number of people with outcomes at baseline and FU |
| Zhou (32) | Estimated | Baseline resilience, anxiety, depression | Adjusted mean difference between two groups at FU, SD of both groups at FU |
| Gonsalves (33) | Estimated |  | Mean and SD for one group at baseline and FU, fixed r=0.5 |
| Arjadi (34) | Estimated | Demographics, marital status, occupation, baseline depression, anxiety | Mean and SD for two groups at FU |
| Araya (35) | Estimated | Brazil trial: Cluster: residency programs; Peru trial: health services; both trials: baseline depression severity | Odds ratio |
| Khan (36) | Estimated | Baseline individual psychological distress | Adjusted mean difference between two groups at FU, corresponding 95% CI |
| Rahman (37) | Estimated |  | Mean and SD for two groups at FU |
| Chen (38) | Observed and estimated | religion, employment status, economic satisfaction, quality of life, social support, Social Network Size, count of comorbidities, hypertension control | Unadjusted outcome: mean and SD for two groups at FU; Adjusted mean difference of two groups at FU, corresponding 95% CI |

Notes: abbreviations: FU = follow-up, CI = confidence-interval, SD= standard deviation

**References:**

1. Glen S. Hedges’ g: Definition, Formula. From StatisticsHowTo.com: Elementary Statistics for the rest of us! [Online]. Available from: https://www.statisticshowto.com/hedges-g/ (last accessed 01.01.2023)

2. Glen S. “F-Test”. From StatisticsHowTo.com: Elementary Statistics for the rest of us! [Online]. Available from: https://www.statisticshowto.com/probability-and-statistics/hypothesis-testing/f-test/ (last accessed 02.01.2023)

3. Lakens D. Calculating and reporting effect sizes to facilitate cumulative science: A practical primer for t-tests and ANOVAs. Front Psychol. 2013;4(NOV):1–12. doi: 10.3389/fpsyg.2013.00863.

4. Glen S. Pooled Standard Deviation [Online]. StatisticsHowTo.com: Elementary Statistics for the rest of us!https://www.statisticshowto.com/pooled-standard-deviation/ (last accessed 05.01.2023)

5. Borenstein, M., Hedges, L.V., Higgins, J.P.T. and Rothstein HR. Effect Sizes Based on Means. In: M. Borenstein, L.V. Hedges JPTH and HRR, editor. Introduction to Meta‐Analysis. 2009. p. 21–32. doi: https://doi.org/10.1002/9780470743386.ch4

6. Higgins J, Thomas J, Chandler J, et al. Cochrane Handbook for Systematic Reviews of Interventions version 5.1.0 (updated March 2011). [Online]. The Cochrane collaboration. 2011. www.handbook.cochrane.org. (last accessed: 19.02.2023)

7. Rosner B. Fundamentals of biostatistics. Brooks/Cole, Boston; 2010. ISBN: 978-0-538-73349-6

8. Rockefeller University. PAD 705 Handout : Standardized Coefficients. Rockefeller College University at Albany (online). 2004. Available from: https://www.albany.edu/faculty/kretheme/PAD705/SupportMat/StandardizedCeof.pdf (last accessed: 20.03.2023)

9. Feingold A. Confidence Interval Estimation for Standardized Effect Sizes in Multilevel and Latent Growth Modeling. J Consult Clin Psychol. 2015;83(1):157–68. doi: 10.1037/a0037721

10. Thalheimer W, Cook S. How to calculate effect sizes from published research: A simplified methodology (online). 2002 Work Learning Recearch. Available from: www.worklearning.com (last accessed 01.11.2023)

11. Rahman A, Akhtar P, Hamdani SU, et al. Using technology to scale-up training and supervision of community health workers in the psychosocial management of perinatal depression: a non-inferiority, randomized controlled trial. Glob Ment Heal. 2019; doi: 10.1017/gmh.2019.7

12. Muke SS, Tugnawat D, Joshi U, et al. Digital Training for Non-Specialist Health Workers to Deliver a Brief Psychological Treatment for Depression in Primary Care in India:Findings from a Randomized Pilot Study. Environ Res public Heal. 2020; doi: 10.3390/ijerph17176368.

13. Nisar A, Yin J, Nan Y, et al. Standardising Training of Nurses in an Evidence-Based Psychosocial Intervention for Perinatal Depression : Randomized Trial of Electronic vs . Face-to-Face Training in China. Int J Environ Res Public Heal. 2022; doi: 10.3390/ijerph19074094.

14. Pereira CA, Wen CL, Miguel EC, et al. A randomised controlled trial of a web ‑ based educational program in child mental health for schoolteachers. Eur Child Adolesc Psychiatry. 2015; doi: 10.1007/s00787-014-0642-8.

15. Maulik PK, Kallakuri S, Devarapalli S, Jha V, Patel A. Increasing use of mental health services in remote areas using mobile technology : a pre – post evaluation of the SMART Mental Health project in rural India. J Glob Health. 2017;7(1).

16. Maulik PK, Devarapalli S, Kallakuri S. The Systematic Medical Appraisal Referral and Treatment Mental Health Project : Quasi-Experimental Study to Evaluate a Technology-Enabled Mental Health Services Delivery Model Implemented in Rural India Corresponding Author : J Med Internet Res. 2020;22(e15553):1–11.

17. Doukani A, Sera F, Chibanda D. A community health volunteer delivered problem-solving therapy mobile application based on the Friendship Bench ‘ Inuka Coaching ’ in Kenya : A pilot cohort study. Glob Ment Heal. 2022;8(e9):1–11.

18. Dambi J, Norman C, Doukani A, Potgieter S, Turner J, Musesengwa R, et al. A Digital Mental Health Intervention (Inuka) for Common Mental Health Disorders in Zimbabwean Adults in Response to the COVID-19 Pandemic: Feasibility and Acceptability Pilot Study. JMIR Ment Heal. 2022;9(10): doi: https://doi.org/10.2196/37968.

19. Chibanda D, Weiss HA, Verhey R, et al. Effect of a Primary Care–Based Psychological Intervention on Symptoms of Common Mental Disorders in Zimbabwe A Randomized Clinical Trial. JAMA. 2016; doi: 10.1001/jama.2016.19102.

20. Ross R, Sawatphanit W, Suwansujarid T, et al. The Effect of Telephone Support on Depressive Symptoms Among HIV-Infected Pregnant Women in Thailand: An Embedded Mixed Methods Study. JANAC J Assoc Nurses AIDS Care. 2013; doi: 10.1016/j.jana.2012.08.005.

21. Ebrahem SM, Badawy SA, Hassan RA, et al.. Effect of Telehealth Nursing Intervention on Psychological Status and Coping Strategies Among Parents During COVID-19 Pandemic. Holist Nurs Pract. 2023; doi: 10.1097/HNP.0000000000000561.

22. Scazufca M, Clara M, Couto PDP, et al. Pilot study of a two-arm non-randomized controlled cluster trial of a psychosocial intervention to improve late life depression in socioeconomically deprived areas of São Paulo , Brazil ( PROACTIVE ): feasibility study of a psychosocial intervention for lntervention for late life depression in Sao Pãulo. BMC Public Health. 2019; doi: 10.1186/s12889-019-7495-5.

23. Garg A, Agrawal R, Velleman R, et al. Integrating assisted tele-psychiatry into primary healthcare in Goa, India: a feasibility study. Glob Ment Heal. 2022; doi: 10.1017/gmh.2021.47.

24. Liu Y, Hasimu M, Joa M, Tang J, Wang Y, He X, et al. The effect of a APP-Based Intervention for Depression Among Community-Dwelling Individuals With Spinal Cord Injury: A randomized Controlled Trial. Arch Phys Med Rehabil. 2023; doi: 10.1016/j.apmr.2022.10.005.

25. Öztoprak PU, Koç G, Erkaya S. Evaluation of the effect of a nurse navigation program developed for postpartum mothers on maternal health: A randomized controlled study. Public Health Nurs. 2023; doi: 10.1111/phn.13226.

26. Hong S, Lee S, Song K, et al. A nurse-led mHealth intervention to alleviate depressive symptoms in older adults living alone in the community: A quasi-experimental study. Int J Nurs Stud. 2023; doi: 10.1016/j.ijnurstu.2022.104431.

27. Noor Hanita Z, Khatijah LA, Kamaruzzaman S. A pilot study on development and feasibility of the ‘MyEducation: CABG application’ for patients undergoing coronary artery bypass graft (CABG) surgery. BMC Nurs. 2022; doi: 10.1186/s12912-022-00814-4.

28. Xu X, Chen S, Chen J, et al.Feasibility and Preliminary Efficacy of a Community-Based Addiction Rehabilitation Electronic System in Substance Use Disorder : Pilot Randomized Controlled Trial. JMIR mHealth uHealth. 2021; doi: 10.2196/21087.

29. Rodriguez M, Eisenlohr-moul TA, Weisman J, et al. The Use of Task Shifting to Improve Treatment Engagement in an Internet-Based Mindfulness Intervention Among Chinese University Students : Randomized Controlled Trial. JMIR Form Res. 2021; doi: 10.2196/25772.

30. Anttila M, Sittichai R, Katajisto J, et al. Impact of a Web Program to Support the Mental Wellbeing of High School Students : A Quasi Experimental Feasibility Study. Environ Res public Heal. 2019; doi: 10.3390/ijerph16142473.

31. Menezes P, Quayle J, Paulo S. Use of a Mobile Phone App to Treat Depression Comorbid With Hypertension or Diabetes : A Pilot Study in Brazil and Peru JMIR Ment Heal. 2019; doi: 10.2196/11698.

32. Zhou K, Li J, Li X. Effects of cyclic adjustment training delivered via a mobile device on psychological resilience , depression , and anxiety in Chinese post ‑ surgical breast cancer patients. Breast Cancer Res Treat. 2019; https://doi.org/10.1007/s10549-019-05368-9

33. Gonsalves PP, Hodgson ES, Bhat B, et al. App- based guided problem- solving intervention for adolescent mental health: a pilot cohort study in Indian schools. Evid Based Ment Heal. 2021; doi: 10.1136/ebmental-2020-300194.

34. Arjadi R, Nauta MH, Scholte WF, et al. Internet-based behavioural activation with lay counsellor support versus online minimal psychoeducation without support for treatment of depression : a randomised controlled trial in Indonesia. The Lancet Psychiatry. 2018; doi: 10.1016/S2215-0366(18)30223-2.

35. Araya R, Menezes PR, Claro HG, et al. Effect of a Digital Intervention on Depressive Symptoms in Patients With Comorbid Hypertension or Diabetes in Brazil and Peru Two Randomized Clinical Trials. JAMA. 2022; doi: 10.1001/jama.2021.4348.

36. Khan MN, Hamdani SU, Chiumento A, et al. Evaluating feasibility and acceptability of a group WHO trans-diagnostic intervention for women with common mental disorders in rural Pakistan: A cluster randomised controlled feasibility trial. Epidemiol Psychiatr Sci. 2019; doi: 10.1017/S2045796017000336.

37. Rahman A, Khan MN, Hamdani SU, Chiumento A, Akhtar P, Nazir H, et al. Effectiveness of a brief group psychological intervention for women in a post-conflict setting in Pakistan: a single-blind, cluster, randomised controlled trial. Lancet. 2019; doi: 10.1016/S0140-6736(18)32343-2.

38. Chen S, Conwell Y, Xue J, et al. Effectiveness of integrated care for older adults with depression and hypertension in rural China: A cluster randomized controlled trial. PLoS Med. 2022;doi: http://dx.doi.org/10.1371/journal.pmed.1004019
