# Supplementary material for: The effectiveness of mental health interventions involving non-specialists and digital technology in low-and middle-income countries – a systematic review
Source: BMC Public Health. 2024 Jan 3;24:77. doi: 10.1186/s12889-023-17417-6 (PMC10763181; doi:10.1186/s12889-023-17417-6)
Supplement: Supplementary file 6 — Additional file 6. [file 12889_2023_17417_MOESM6_ESM.docx]

# **ADDITIONAL FILE 6: DETAILED INTERVENTION DESCRIPTION**

**Table S6. Detailed intervention descriptions**

| **Author,**  **year** | **Name of intervention** | **Intervention type and summary** | **Control type and summary** | **Duration and frequency of intervention** | **Mode of intervention-delivery** | **Type and characteristics of non-specialist** | **Role of non-specialist** | **Type of training and supervision of non-specialists** | **Type of technology** | **Role of technology** |
| --- | --- | --- | --- | --- | --- | --- | --- | --- | --- | --- |
| **Rahman, 2019** (1) | Technology-Assisted Cascade Training and Supervision (TACTS) | Type: Training  Intervention to train and supervise non-specialists through group sessions on the provision of treatment for perinatal depression based on the thinking Healthy Program (THP). | Conventional face-to-face training: receiving training by a THP master trainer through lectures, group discussions, role plays, and feedback on the role plays by  the trainers and peers, using THP training materials (THP training  manual and job aid). | 5 days (20h in total) | Face-to-face | Lady health workers (LHW) | Face-to-face:  Receives training from non-specialist THP trainer in form of narrative THP scripts, fictional scenarios of training material using AVATARs, short videos, interactive role-plays, discussions with peers and lay health supervisors, reflections, and problem-solving strategies*.* | Training: *see role of non-specialist;* time: *see duration and frequency of intervention*  Supervision: face-to-face peer group supervision led by Lay help workers including reflections, discussions and motivation promotion; time: 1x month | Multimedia android-based application | Mobile application:  Delivering training content and exercises on THP. |
| **Muke, 2020** (2) | Digital training with remote support (DGT+) | Type: Training  *Same as above* + remote support. | n.a | 30 days (total of 48h) | Remotely | ASHA^3^, ASHA^3^ Facilitators, Multi-Purpose Health Workers | Face-to-face:  Receives short orientation by research staff.  Remotely:  Receives digitalized HAP training manuals including expert lecture videos, role-play videos of clinical scenarios, PowerPoint presentation,  reading materials, interactive quizzes, and assessment questions. Training was provided by research staff and the digital tool + Receives regular supervision. | Training:  *see role of non-specialist;* time: *see duration and frequency of intervention;*  Supervision: regular phone calls with research staff for technical and content assistance, adherence and motivational support; time: weekly | Mobile application, phone calls | Mobile application:  *Same as above*  Phone calls: Enables communication for remote supervision purposes. |
|  | Digital training (DGT) | Type: Training  Intervention to train non-specialists through group sessions on the provision of brief psychological treatment based on the Healthy Activity program (HAP). |  |  |  |  | Face-to-face:  Receives short orientation by research staff.  Remotely:  Same as above without regular group supervision (only on request) | Training: *see role of non-specialist;* time: *see duration and frequency of intervention*  Supervision: phone calls with research staff for technical assistance, if required; time: only on request | Mobile application, phone | Mobile application: delivering training on HAP.  Phone: communication with research staff for technical assistance |
| **Nisar, 2022** (3) | E-training | Type: Training  Intervention to train non-specialists through group sessions on the provision of treatment for women with perinatal depression based on the thinking healthy program (THP). | Face-to-face class-room based training by a specialist | 5 days | Face-to-face | Nursing students | Face-to-face:  Receives training in form of videos, exercises, discussions and role-plays with support by regular tutor. Training was provided by regular tutor + tablet-based multimedia tool. | Training & duration: *see role of non-specialist;* time: *see duration and frequency of intervention*  Supervision: n.m. | Tablet-based multimedia tool | Delivering training content and exercises on THP. |
| **Pereira, 2015** (4) | Web-based interactive education (WBIE) | Type: Education and training  Intervention to educate and train  teachers through group sessions on (handling & identifying) child mental disorders. | n.a | 3h per week for 3 weeks (9h in total) | remotely | teachers | Remotely:  Receives training and education through educational videos + booklet on child mental disorders, instructions on how to use *the Strength and Difficulty Questionnaire*, classroom management principles, discussions with all teachers and psychiatrists | Training: *see role of non-specialist;* time: *see duration and frequency of intervention*  Supervision & time: n.m. | Website | Delivering educational program on child mental health and enabling interaction with professionals and peers through web-conferences. |
|  | Text- and video-based education (TVBE) | Same as above | n.a | same | remotely | same | *The intervention includes all the above-mentioned components only using video-and text-without the web-conference with peers and psychiatrists* | Same | DVD | Visualisation tool for educational purpose on child mental health disorders. |
| **Maulik, 2020 and Maulik, 2017** (5,6) | The Systematic medical appraisal referral and treatment (SMART) | Type: Promotion, prevention and treatment  Intervention aiming to increase the use of MHC^2^ services and treat people with CMD^1^ using following components:  1) Delivery of anti-stigma campaign through group sessions  2) ASHAs^3^ screen for CMD^1^ through individual sessions  3) Doctors diagnose and treat people with CMD^1^ through individual sessions  4) Doctors and ASHAs^3^ following up on patients through individual sessions | n.a | Maulik 2017: 3 months, frequency: unclear; Maulik 2020: 1 year, frequency: unclear | Face-to-face | ASHA^3^:  Lay female village health workers who receive basic health  care training (primary focus on maternal and child health) with 8-10 years of education.  Doctors:  medical doctors without specialization in mental health | ASHA^3^:  Face-to-face:  screens population for CMD^1^.  Doctors:  Face-to-face:  Diagnoses and treats CMD^1^. | ASHA^3^:  Training: identification and management of CMDs^1^ using videos, presentation, and case vignettes discussions delivered by research staff; time: 2 -10 days  Supervision & time: n.m.  Doctors:  Training:  On the use of the mhGAP-IG through presentations and case vignettes discussion delivered by clinical psychiatrist; time: 1 day  Supervision: By psychiatrists + research team; time: not specified, but decreases over time | Application on android tablet, videos | Application: ASHA^3^  use the electronic decision support systems (EDSS) which is based on PHQ-9^4^ and GAD-7^5^ to screen patients. Doctors use the EDSS, which is based on mhGAP-IG, to diagnose and treat patients. Both collect treatment and adherence data with the application.  Videos: showing videos to reduce the mental health stigma  *Note: for our systematic review we just investigated how the screening by non-specialist influenced healthcare use, hence patient-related outcomes after receiving care provided by the non-specialised doctors or stigma-related was not assessed. Additionally, stigma-related outcomes after receiving anti-stigma campaigns were not assessed. |
| **Doukani, 2021** (7) | Inuka coaching app | Type: Treatment  Intervention aiming to treat CMD^1^ through low-intensity individual treatment sessions based on problem-solving techniques. | n.a. | 4 weekly or biweekly sessions (60 minutes per session) | Remotely | Community health volunteer:  Existing Community health volunteers,  ability to speak and write in English, owns and regularly uses smartphone | Remotely:  Delivers low-intensity problem-solving therapy to treat common mental disorders and refers them to specialists in case of non-adherence or aggravation of symptoms. | Training: introduction to problem-solving therapy, step-by-step guide, mock practice session by senior training specialist and through text material; time: 3 days  Supervision: regular meetings with psychologist and research team member + one-to-one meeting with psychologist for own mental well-being; time: 4 supervisor meetings + 2 psychological meetings. | Mobile app, Chat-based service on mobile phone | Mobile app:  Decision support system (including screening), data collection & monitoring (including monitoring disease progression and referral to specialists)  Chat-function: communication between non-specialist & receiver (The non-specialist delivers the treatment using the chat-function) |
| **Dambi. 2022** (8) | Inuka coaching app | Type: Treatment and prevention  Intervention aiming to treat common mental disorders through individual sessions based on problem-solving techniques. | n.a | 6 sessions (duration & frequency not stated) | remotely | Community volunteers | Remotely: Delivers the digital intervention | Training: trained in problem-solving therapy; time: unclear  Supervision: by psychologists and psychiatrists | Smartphone app | Mobile app:  Decision support system (including screening), data collection & monitoring (including monitoring disease progression and referral to specialists)  Chat-function: communication between non-specialist & receiver (The non-specialist delivers the treatment using the chat-function) |
|  | Friendship-based whatsapp intervention | Type: treatment and prevention  Intervention aiming to treat common mental disorders through individual sessions based on problem-solving techniques. | n.a | 6 sessions (duration & frequency not stated) | remotely | Community volunteers | Remotely: Delivers the digital intervention | Training: trained in problem-solving therapy; time: unclear  Supervision: by psychologists and psychiatrists | Smartphone app (Whatsapp) |  |
| **Chibanda, 2016** (11) | The friendship bench intervention | Type: Treatment  Intervention aiming to treat common mental disorders through individual sessions based on problem-solving techniques. | Enhanced usual care: standard care including nurse-led evaluation, brief support counselling, medication, education and support on CMD^1^, referral to specialist if needed. | 6 sessions, 4-6 weeks | Face-to-face and remotely | Lay health worker:  Female, mean age: 53 years,  mean education period: 10 years, ability to use mobile phone, resides near clinical setting | Face-to-face:  Provides problem solving therapy to patients and refers them to specialists in case of non-adherence or aggravation of symptoms.  Remotely:  Supports therapy adherence. | Training:  based on an intervention manual about common mental disorders, counselling skills, problem solving therapy, and self-care; time: 9 days  Supervision: individual meetings with trained senior health supervision officer; time: n.m. | Mobile phone (text-messages or phone calls) | 1. Sending motivational texts; 2. Sending reminders in case of non-adherence 3. Encouraging treatment adherence |
| **Ross, 2013** (9) | Telephone support intervention | Type: Prevention and treatment  Intervention to improve depressive symptoms in HIV-infected pregnant women through individual sessions using emotional and informational support strategies. | Routine prenatal service: regular prenatal care + education on HIV self-care | 1 x week + as needed (15-30 min) for 2 months | remotely | Registered nurse | Remotely:  Provides emotional and informational support for pregnant women with HIV | Training & time: n.m  Supervision & time: n.m. | Phone | Communication between non-specialist and service receiver |
| **Ebrahem, 2023** (10) | Telehealth nursing intervention | Type: prevention  Intervention to support psychological wellbeing of parents during COVID pandemic through emotional support | n.a. | Not mentioned | Remotely | nurses | Remotely: provides COVID-related information, provides coping strategies (i.e., breathing exercises etc.) | Training & time: n.m  Supervision & time: n.m. | Phone | Communication between non-specialist and service receiver |
| **Scazufca, 2019** (12) | Psychosocial intervention | Type: Treatment  Intervention to treat depression in older adults through individual sessions based  on behavioural activation, psychoeducation and relapse prevention techniques using a stepped care approach. | Enhanced usual care: (1) identification of depression and (2) additional training of nurses and family doctors to the usual care. | 17 weeks; Weekly (and biweekly for less-severe cases); 60 minutes on average | Face-to-face | Nurse assistant or community health worker | Face-to-face:  Delivers psychosocial treatment based on the stepped-care approach (with different non-specialists) | Training:  Face-to-face training + lecturer on (identification of) depression, depression care for elderly, principles of the intervention by research psychologists and psychiatrist; time: 3 days + 90 min lecturer + 1h discussion  Supervision:  Group discussions on cases and session contents delivered by clinical psychologist + individual support as required; time: first weekly, then biweekly | Tablet application | Provides a decision-support tool, managing and monitoring appointments, monitoring adherence and psychosocial data, notification to clinic manager about patients and intervention deliverer (psychological state, adherence etc.), audio-recording sessions for supervision purposes |
| **Garg, 2022** (14) | Tele-psychiatric treatment | Type: Treatment  Assisted tele-psychiatry provided by specialist who is supported by non-specialist | n.a | Based on individual needs | Face-to-face and remotely | Non-specialists who were educated at least on senior secondary level and have experience in providing other task-shifting interventions | Remotely: mental health status (family-history, medical history, suicide risk) assessment, briefing psychiatrist on current mental health status  Face-to-face: physical examination (weight, pulse, blood pressure), liaising with service-receivers family members, providing counselling for depression or harmful drinking. | Training: received training on delivering task-shifting interventions (Healthy Activity Program and Counselling for alcohol problems); time: n.m  Supervision: n.m. | Electronic Medical Record (EMR) and telemedicine web application | Care coordination: appointing schedules, medicine prescriptions; providing psychiatrist with necessary health-related information; Data collection: history taking format, generating and sharing notes and files. |
| **Liu, 2023** (16) | Together application | Type: prevention  Nurse-led collaborative care (with specialised care worker) + digital program for education and mental health assessment purposes | Care as usual for people with spinal cord injury+ 1  telephone follow-up by a nurse at week 12 (after hospital discharge) for additional spinal cord injury care (advice on skin care, defecation management, selfcare and functioning training) + health education CDs | 5 sessions (at week 2,4,6,8 and 12 after hospital discharge) for 12 weeks | remotely | nurses, rehabilitation physicians, physiotherapists, and occupational therapist | Remotely: providing usual care + guiding participants with the self-management app + providing structured support based on the decision support app + referral to specialists | Training: based on implementation process and the use of the health app (+ other important matters that are not specified), duration: n.m; supervision: n.m. | Mobile application | 1.Provides mental health education + structured mental health follow-up assessments, 2. Communication with health workers, 2. Referral function, |
| **Öztroprak** (13) | Nurse navigation program-based interventions | Type: prevention  Nurses provide post-natal care including psychosocial components | Care as usual:  standard hospital post-natal care (without psychosocial components) + counselling booklet on request | 12 weeks | Face-to-face and remotely | Nurses | Face-to-face: prepares mothers for childbirth, trains them in self-care and baby care, evaluates home environment, provides health information and training and counselling for maternal and baby care, accompanies mother to doctors appointment; remotely: sending supportive and reminder SMS for medication adherence, phone counselling on problems | Training: n.m.; Supervision: n.m. | Phone call & SMS | Phone: Communication for counselling sessions, SMS: alerts and reminders for medication adherence and providing supportive messages |
| **Hong, 2023** (15) | mHealth Intervention | Type: Treatment  Nurse-led mHealth intervention for elderly people with depression | Care as usual for elderly people at community healthcare centre + general information on available mental health services in the community | 1-2 hours 5 days a week for 4 weeks | Face-to-face and remotely | Nurses + community nurses | Face-to-face: provision of mHealth services, consultation of intervention content, teaching and resolving technical issues, supporting adherence and motivation. | Training: based on clinical research information service on how to provide the device training, personalise intervention content, assisting participants with mHealth intervention and technical issues; time: n.m. | mHealth app + other health-related apps | mHealth app: cognitive behavioural therapy such as problem-solving skills, relaxation techniques, and practising social skills that address depression, online/offline art activities such as drawing, painting, and colouring, and some non-pharmacological apps |
| **Hanita, 2022** (17) | MyEducation: CABG | Type: prevention  App-based educational intervention including psychosocial related tasks for people with coronary artery bypass  graft surgery | Care as usual: regular care for people undergoing a coronary artery bypass  grafting surgery | 2 education sessions of 15-20 minutes + daily diary writing (for 1 month) | Remote + face-to-face | nurses | Implementing the digital intervention (i.e., explaining installation) + emergency contact for patients | Training & supervision: n.m., | Mobile application | 1.Sending alerts to nurses; 3. Providing education on CABG pre and post-surgery + delivers self-management skills including diary function to record feelings. |
| **Xu, 2021** (18) | Community-based addiction rehabilitation electronic system  (CAREs) + Community-Based Rehabilitation | Type: Treatment  Intervention to provide community-based rehabilitation for people with drug abuse disorders. | Usual community-based Rehabilitation | >=1x weekly for 6 months | Face-to-face and remotely | Social worker from the community-based addiction rehabilitation program in China | Face-to-face:  Provides care as usual (weekly urine screen, application for social benefits)  Remotely:  using the CARE website to obtain health-related information, track location of participant, track and support adherence to treatment, and supports motivation. | Training & time: n.m.  Supervision & time: n.m. | Mobile application, website | Molbile application: provides education, assessment,  coping skills, (peer and social worker) support using text and videos. Provides the possibility to use a SOS module to immediately connect with family member, social worker, and doctor if necessary.  Website: Social worker obtains all health-related information of their patient + uses the platform to communicate with patient. |
| **Rodriguez, 2021** (19) | MIND+ | Type: Prevention and treatment  Intervention that aims to reduce stress and depression and increase mindfulness through individual sessions based on elements of mindfulness-based cognitive therapy with non-specialist support. | MIND intervention: individual sessions with digital app based on elements of mindfulness-based cognitive therapy without non-specialist support | 4 weeks | remotely | Students:  Inclusion currently enrolled at the university, has a smartphone and regular internet access, ability to read and communicate in Mandarin and English, willing to participate and complete this intervention  Exclusion  Aged <18 years, reports previous or current training in mindfulness or psychotherapy, reports current treatment for a mental health problem, unable to attend the training. | Remotely:  Encourages and supports treatment adherence. | Training:  Face-to face training on mindfulness, ethics & confidentiality, counselling skills, validation techniques, motivational interviewing based on including lecturers, practice sessions, feedback & coaching from research group members; time: 8h  Supervision:  Remote supervision meetings based on dialectical behaviour therapy  consultation team meetings including discussions, reflections, support; time: weekly | Multi-media website, zoom, phone (we-chat, calls) | Website: Delivering mindfulness intervention  Zoom: Enables communication for supervision purposes.  Phone (we-chat & calls): Enables communication between non-specialist and receivers. |
| **Anttila 2019** (20) | DepisNet-Thai intervention | Type: Promotion  Intervention aiming to support adolescent mental wellbeing through peer-group sessions, based on Garcia’s construction of adolescent coping. | n.a | 5 weekly sessions for 5 weeks (50 minutes per session) + 2 weeks orientation | Face-to-face | Teacher | Face-to-face: Runs the program, acts as tutor, guides students on answering the questions in the app, reads students exercises, interacts in peer-group discussions, monitors & supports adherence | Training: handbook for DepisNet-Thai + course; time: n.m.  Supervision: n.m.; time: n.m. | Web program | Providing treatment component:  1.Delivering mental health promotion techniques through exercises that support self-reflection skills and self-management skills. |
|  | DepisNet-Thai intervention active control | Type: Promotion  Intervention aiming to support adolescent mental wellbeing through individual sessions, based on Garcia’s construction of adolescent coping | n.a | Same as above | Same as above | Same as above | Same as above without peer-group discussion | Same as above | Same as above |  |
| **Menezes, 2019** (21) | Emotional control (CONEMO) | Type: Prevention & treatment  Intervention aiming to reduce depressive symptoms in people with diabetes or hypertension through individual low-intensity psychoeducation sessions based on behavioural activation techniques. | n.a | 3 sessions per week for 6 weeks | Face-to-face and remotely | Nurse or nurse assistant | Face-to-face:  Introduces the app, closes the final session.  Remotely:  Resolves questions related to the app, supports adherence and motivation. | Training: n.m; time: n.m  Supervision: by clinical psychologist; time: weekly | Mobile phone application, phone | Delivering the behavioural activation program.  Phone:  Enables communication between nurse/nurse assistant and patient. |
| **Zhou, 2019** (22) | Cyclic Adjustment  Training (CAT) | Type: Prevention & treatment  Intervention to increase psychological resilience and reduce depressive and anxiety symptoms in women with breast cancer through individual sessions based on the CAT. | Routine nursing care for woman with breast cancerara: including health instruction, vital signs and post surgery complications monitoring, and post-surgery and drainage tube care. | 12 weeks (ranging from 1x before surgery for 20-60 min, to 1x weekly to 3x a day including different tasks) | Face-to-face & remotely | Nurse | Face-to-face & remotely (after hospital discharge):  Provides routine nursing care + supports the use of the CAT intervention based on the Roy Adaptation Model (RAM). | Training & time: n.m  Supervision & time: n.m | Mobile application (CAT + WeChat) | CAT application: Delivering psychosocial intervention.  WeChat: Enables communication between user and peers and user and nurse. |
| **Gonsalves, 2021**(23) | POD  Adventures | Type: Treatment  Intervention aiming to reduce psychosocial and mental health problems in students (with subjective mental health problems) through individual and group sessions, based on problem-solving techniques. | n.a. | 4 sessions over 2-3 weeks (30-40 minutes per session) | Face-to-face | Lay counsellors: college graduates with experience in delivering the problem-solving intervention | Face-to-face:  Introduces the app, guides and supports the users in technical or content issues. | Training: office-based course + printed manual; time: 4 days  Supervision: peer group supervisor meetings with clinical psychologist; time: weekly (1h per session) | Mobile application | Delivering problem-solving therapy |
| **Arjadi, 2018** (24) | Guided act and Feel Indonesia (GAF-ID) | Type: Treatment  Intervention aiming to treat depressive symptoms through individual sessions including:  psychoeducation about depression, monitoring mood and behaviour or activities,  behavioural activation techniques. | Online psychoeducation: basic psychoeducation about depression. | 1x weeks for 8 weeks (30-45 min per session) | Remotely | Lay counsellors | Remotely:  Introduces the web-program, supports and provides feedback on completed modules, technical assistance, supports adherence. | Training & time: course on intervention with role-plays + how to handle technical issues, people with low motivation, and how to monitor people with suicidality and other serious aggravation of symptoms + written manual as a support during the intervention; time: 2 days.  Supervision: time: regular meeting with psychologist and sending reports; time: weekly (30-60 minutes) + further phone calls if necessary | Web program, phone calls, email | Web program:  1.Delivering treatment for depression based on psychoeducation and behavioural activation techniques;  Web-program/ phone call/email:  1. Sending reminders to participant; 2. Enabling communication between lay counsellor and user on app-content. 3. Supervision of non-specialist (if necessary, via phone) |
| **Araya, 2022** (25) | Digital intervention | Type: Treatment  Low-intensity Intervention aiming to treat depression through individual sessions based on behavioural activation techniques. | Enhanced usual care: treatments as usual for depression, diabetes and/or hypertension (type of treatment left to the discretion of local clinicians) + regular assessment for depression outcomes | 3 sessions per week for 6 weeks (<=10 minutes per session) | Face-to-face & remotely | Nurse assistant | Face-to-face:  Introduces the app  Remotely:  Supports patients in using the app, resolves (technical) difficulties & supports adherence | Training: yes *(no further details mentioned);* time: n.m.  Supervision: individually by clinical psychologist; time: weekly | Mobile application, phone calls | Mobile application:  1.Delivering low-intensity treatment to reduce depressive symptoms through behavioural activation techniques.  2.Sending alerts and reminders to the non-specialist in case of non-adherence of participants.  Phone call:  1. Enables communication between nurse assistant and user for support on app-related tasks; 2. Supporting adherence by having 2 mandatory calls in the beginning. |
| **Khan, 2019** (26) | Group problem management + | Type: Prevention & treatment  Intervention aiming to prevent and treat common mental disorders in women through group sessions based on problem-solving, counselling and behavioural techniques using the WHO-based transdiagnostic guidelines. | Enhanced usual care:  Referral of people with CMD^1^; receiving brief treatment from primary care physicians on CMD^1^. | 5 weekly sessions (2 h per session) | Face-to-face | Lay helpers:  females with 16 years of education and without formal mental health education.  Lady health workers (LHW):  certified LHW, who usually work at a health unit and provides maternal and child health services. | Lay helpers:  Face-to-face:  Delivers the Group PM+  Lady health workers:  Face-to-face:  Introduces lay helpers to the community, supports participant adherence, provides space for intervention sessions. | Lay helper:  Training:  by master trainer on knowledge  of common mental disorders, basic counselling and  group management skills, the Group PM+ intervention, self-care strategies, and practice sessions; time: 10 days  Supervision:  Remote group supervision with peers and non-specialists, trained supervisors; duration: weekly (2h per session)  Lady health worker:  Training:  by lay helpers on roles and responsibilities; time: ½ days  Supervision: n.m | Skype | Enables communication for supervision purposes. |
| **Rahman, 2019** (27) | Group management + | Type: Prevention & treatment  Intervention aiming to prevent and treat common mental disorders in women through group sessions based on problem-solving, counselling and behavioural techniques using the WHO-based transdiagnostic guidelines. | Enhanced usual care: feedback on assessment results and psychoeducation for participants and their families. | 5 weekly sessions (2 h per session) | Face-to-face | Facilitators: females with 16 years of education and without formal mental health education.  Lady health workers (LHW): certified LHW, who usually work at a health unit and provides maternal and child health services. | Facilitators: delivering the Group PM+  LHW: providing logistical support | Facilitators: by master trainer in form of education on adversities and its effect on mental health, basic helping skills, delivery of intervention strategies, skills in group facilitation, and facilitating self-care; time: 7 days.  Supervision: Remote group supervision with experienced supervisors including reviewing participants progress, individual case management, refresher training on strategies, rehearsing skills through role plays; duration: weekly (2h per session)  LHW: Training: n.m., supervision: n.m | Skype | Enables communication for supervision purposes. |
| **Chen, 2022** (28) | Chinese  Older Adult Collaborations in Health | Type: treatment  Two non-specialsits (primary care physician + aging workers) provide treatment of older people with hypertension and depressive symptoms. | Enhanced usual care: primary care physician receives guidelines to provide depression medication. | Monthly for 12 months | Face-to-face | Aging workers: people with middle-school education who receive in-service training from the Bureau of Civil Affairs in addressing the villagers’ social  needs.  Primary care physician: 3 years of medical education after high school without specific guidelines or training for affective disorder treatment. | Aging workers: Reduce social barriers to effective Depression and hypertension management, Support adherence to depression and hypertension treatment recommendations, providing health education,  Primary care physician: provides treatment for depression and hypertension based on treatment guidelines; provides health education  Both: weekly meeting to review care coordination | Aging workers & primary care physician: 4 days of training on their respective roles in this intervention.  Supervision: monthly call with psychiatrist + more on request | Phone | Enables communication for supervision purposes |

**References:**

1. Rahman A, Akhtar P, Hamdani SU, et al. Using technology to scale-up training and supervision of community health workers in the psychosocial management of perinatal depression: a non-inferiority, randomized controlled trial. Glob Ment Heal. 2019; doi: 10.1017/gmh.2019.7

2. Muke SS, Tugnawat D, Joshi U, et al. Digital Training for Non-Specialist Health Workers to Deliver a Brief Psychological Treatment for Depression in Primary Care in India:Findings from a Randomized Pilot Study. Environ Res public Heal. 2020; doi: 10.3390/ijerph17176368.

3. Nisar A, Yin J, Nan Y, et al. Standardising Training of Nurses in an Evidence-Based Psychosocial Intervention for Perinatal Depression : Randomized Trial of Electronic vs . Face-to-Face Training in China. Int J Environ Res Public Heal. 2022; doi: 10.3390/ijerph19074094.

4. Pereira CA, Wen CL, Miguel EC, et al. A randomised controlled trial of a web ‑ based educational program in child mental health for schoolteachers. Eur Child Adolesc Psychiatry. 2015; doi: 10.1007/s00787-014-0642-8.

5. Maulik PK, Kallakuri S, Devarapalli S, Jha V, Patel A. Increasing use of mental health services in remote areas using mobile technology : a pre – post evaluation of the SMART Mental Health project in rural India. J Glob Health. 2017;7(1).

6. Maulik PK, Devarapalli S, Kallakuri S. The Systematic Medical Appraisal Referral and Treatment Mental Health Project : Quasi-Experimental Study to Evaluate a Technology-Enabled Mental Health Services Delivery Model Implemented in Rural India Corresponding Author : J Med Internet Res. 2020;22(e15553):1–11.

7. Doukani A, Sera F, Chibanda D. A community health volunteer delivered problem-solving therapy mobile application based on the Friendship Bench ‘ Inuka Coaching ’ in Kenya : A pilot cohort study. Glob Ment Heal. 2022;8(e9):1–11.

8. Dambi J, Norman C, Doukani A, Potgieter S, Turner J, Musesengwa R, et al. A Digital Mental Health Intervention (Inuka) for Common Mental Health Disorders in Zimbabwean Adults in Response to the COVID-19 Pandemic: Feasibility and Acceptability Pilot Study. JMIR Ment Heal. 2022;9(10): doi: https://doi.org/10.2196/37968.

9. Chibanda D, Weiss HA, Verhey R, et al. Effect of a Primary Care–Based Psychological Intervention on Symptoms of Common Mental Disorders in Zimbabwe A Randomized Clinical Trial. JAMA. 2016; doi: 10.1001/jama.2016.19102.

10. Ross R, Sawatphanit W, Suwansujarid T, et al. The Effect of Telephone Support on Depressive Symptoms Among HIV-Infected Pregnant Women in Thailand: An Embedded Mixed Methods Study. JANAC J Assoc Nurses AIDS Care. 2013; doi: 10.1016/j.jana.2012.08.005.

11. Ebrahem SM, Badawy SA, Hassan RA, et al.. Effect of Telehealth Nursing Intervention on Psychological Status and Coping Strategies Among Parents During COVID-19 Pandemic. Holist Nurs Pract. 2023; doi: 10.1097/HNP.0000000000000561.

12. Scazufca M, Clara M, Couto PDP, et al. Pilot study of a two-arm non-randomized controlled cluster trial of a psychosocial intervention to improve late life depression in socioeconomically deprived areas of São Paulo , Brazil ( PROACTIVE ): feasibility study of a psychosocial intervention for lntervention for late life depression in Sao Pãulo. BMC Public Health. 2019; doi: 10.1186/s12889-019-7495-5.

13. Garg A, Agrawal R, Velleman R, et al. Integrating assisted tele-psychiatry into primary healthcare in Goa, India: a feasibility study. Glob Ment Heal. 2022; doi: 10.1017/gmh.2021.47.

14. Liu Y, Hasimu M, Joa M, Tang J, Wang Y, He X, et al. The effect of a APP-Based Intervention for Depression Among Community-Dwelling Individuals With Spinal Cord Injury: A randomized Controlled Trial. Arch Phys Med Rehabil. 2023; doi: 10.1016/j.apmr.2022.10.005.

15. Öztoprak PU, Koç G, Erkaya S. Evaluation of the effect of a nurse navigation program developed for postpartum mothers on maternal health: A randomized controlled study. Public Health Nurs. 2023; doi: 10.1111/phn.13226.

16. Hong S, Lee S, Song K, et al. A nurse-led mHealth intervention to alleviate depressive symptoms in older adults living alone in the community: A quasi-experimental study. Int J Nurs Stud. 2023; doi: 10.1016/j.ijnurstu.2022.104431.

17. Noor Hanita Z, Khatijah LA, Kamaruzzaman S. A pilot study on development and feasibility of the ‘MyEducation: CABG application’ for patients undergoing coronary artery bypass graft (CABG) surgery. BMC Nurs. 2022; doi: 10.1186/s12912-022-00814-4.

18. Xu X, Chen S, Chen J, et al.Feasibility and Preliminary Efficacy of a Community-Based Addiction Rehabilitation Electronic System in Substance Use Disorder : Pilot Randomized Controlled Trial. JMIR mHealth uHealth. 2021; doi: 10.2196/21087.

19. Rodriguez M, Eisenlohr-moul TA, Weisman J, et al. The Use of Task Shifting to Improve Treatment Engagement in an Internet-Based Mindfulness Intervention Among Chinese University Students : Randomized Controlled Trial. JMIR Form Res. 2021; doi: 10.2196/25772.

20. Anttila M, Sittichai R, Katajisto J, et al. Impact of a Web Program to Support the Mental Wellbeing of High School Students : A Quasi Experimental Feasibility Study. Environ Res public Heal. 2019; doi: 10.3390/ijerph16142473.

21. Menezes P, Quayle J, Paulo S. Use of a Mobile Phone App to Treat Depression Comorbid With Hypertension or Diabetes : A Pilot Study in Brazil and Peru JMIR Ment Heal. 2019; doi: 10.2196/11698.

22. Zhou K, Li J, Li X. Effects of cyclic adjustment training delivered via a mobile device on psychological resilience , depression , and anxiety in Chinese post ‑ surgical breast cancer patients. Breast Cancer Res Treat. 2019; https://doi.org/10.1007/s10549-019-05368-9

23. Gonsalves PP, Hodgson ES, Bhat B, et al. App- based guided problem- solving intervention for adolescent mental health: a pilot cohort study in Indian schools. Evid Based Ment Heal. 2021; doi: 10.1136/ebmental-2020-300194.

24. Arjadi R, Nauta MH, Scholte WF, et al. Internet-based behavioural activation with lay counsellor support versus online minimal psychoeducation without support for treatment of depression : a randomised controlled trial in Indonesia. The Lancet Psychiatry. 2018; doi: 10.1016/S2215-0366(18)30223-2.

25. Araya R, Menezes PR, Claro HG, et al. Effect of a Digital Intervention on Depressive Symptoms in Patients With Comorbid Hypertension or Diabetes in Brazil and Peru Two Randomized Clinical Trials. JAMA. 2022; doi: 10.1001/jama.2021.4348.

26. Khan MN, Hamdani SU, Chiumento A, et al. Evaluating feasibility and acceptability of a group WHO trans-diagnostic intervention for women with common mental disorders in rural Pakistan: A cluster randomised controlled feasibility trial. Epidemiol Psychiatr Sci. 2019; doi: 10.1017/S2045796017000336.

27. Rahman A, Khan MN, Hamdani SU, Chiumento A, Akhtar P, Nazir H, et al. Effectiveness of a brief group psychological intervention for women in a post-conflict setting in Pakistan: a single-blind, cluster, randomised controlled trial. Lancet. 2019; doi: 10.1016/S0140-6736(18)32343-2.

28. Chen S, Conwell Y, Xue J, et al. Effectiveness of integrated care for older adults with depression and hypertension in rural China: A cluster randomized controlled trial. PLoS Med. 2022;doi: http://dx.doi.org/10.1371/journal.pmed.1004019
