# Supplementary material for: The effectiveness of mental health interventions involving non-specialists and digital technology in low-and middle-income countries – a systematic review
Source: BMC Public Health. 2024 Jan 3;24:77. doi: 10.1186/s12889-023-17417-6 (PMC10763181; doi:10.1186/s12889-023-17417-6)
Supplement: Supplementary file 7 — Additional file 7. [file 12889_2023_17417_MOESM7_ESM.docx]

# Additional file 7 Detailed outcome description and effect sizes

**Table S7.1. Overview of outcome categorization**

| **Outcome category** | **Specific outcomes** | **Study references** |
| --- | --- | --- |
| Competence | Competence | (1), (2), (3) |
| Knowledge | Knowledge | (4) |
| Mental healthcare use | Mental healthcare use | (5), (6) |
| Severity of mental health problems | Severity of anxiety, Severity of depression, Severity of common mental disorder (CMD), disability, Proportion of people with depression (mild-severe) vs no depression, drug positive tests, Longest period of drug abuse absence, Indices of depression, Indices of anxiety, Indices of stress, stress level, proportion of people recovered from depression, Proportion of people with disability at baseline who had no disability at follow-up, Proportion of participants without suicide risk at follow-up, psychosocial problem severity, Mental health symptoms, Psychological distress in terms of hospital anxiety and depression, psychological profile, PTSD symptoms, generalised distress, Severity of alcohol use disorder symptoms | (7), (8), (9), (10), (11), (12), (14), (15), (16), (17), (18), (19), (20), (21), (23), (26) |
| Psychosocial functioning | Quality of life, Capability, mindfulness, level of resilience, mental wellbeing | (8), (15), (19), (22), (23) |

**Table S7.2 Detailed effect sizes for each intervention**

| **Study author, intervention name, reference** | **Outcome** | **Effect size calculation (follow-up assessment time-point)** | **Unadjusted effect size^1^** | **Adjusted effect size^1^** | **Adjusted variables** | **Favours intervention vs. control** | **Favours post intervention vs. baseline** |
| --- | --- | --- | --- | --- | --- | --- | --- |
| ***Digital training of non-specialists; n=4 studies (6 interventions)*** | | | | | | | |
| Rahman, Digital training with face-to-face support (1) | Competence | Intervention – control (3 months post intervention) | d=0.16, small |  |  | Intervention |  |
| Muke, intervention 1: Digital training with remote support (DGT+) (2) | Competence | Intervention – baseline | d=0.7, medium |  |  |  | Post intervention |
| Muke, intervention 2: Digital training without remote support (DGT) (2) | Competence | Intervention – baseline | d=0.32, medium |  |  |  | Post intervention |
| Nisar, Digital training with face-to-face support (3) | Competence | Intervention – control (3 months post intervention) | d=0.1, small | d=0.1, small | Baseline socio demographics, prior mental health training and knowledge | Control |  |
| Pereira, intervention 1: Web-based interactive education intervention (4) | Knowledge | Intervention – baseline |  | d=0.1, small | Clusters (schools) |  | Post intervention |
| Pereira, intervention 2: Text-and video-based education (4) |  |  |  | d=0.08, small | Clusters (schools) |  | Baseline |
| ***Digital support for non-specialists; n=11 studies (12 interventions)*** | | | | | | | |
| Maulik, The SMART intervention (5) | Mental healthcare use | Intervention – baseline | d=0.68, medium |  |  |  | Post intervention |
| Maulik, The SMART intervention (6) | Mental healthcare use | Intervention – baseline | d=1.17, large | d=1.18, large | Cluster (villages), baseline sociodemographic |  | Post intervention |
| Doukani, Inouka coaching app intervention (7) | Severity of anxiety | Intervention – baseline | d=0.83, large |  |  |  | Post intervention |
|  | Severity of depression |  | d=0.84, large |  |  |  | Post intervention |
|  | Severity of CMD |  | d=1, large |  |  |  | Post intervention |
| Dambi, intervention 1: Inouka coaching app intervention (8) | Severity of CMD | Intervention – baseline | d=1.08, large |  |  |  | Post intervention |
|  | Severity of depression |  | d=0.71, medium |  |  |  | Post intervention |
|  | Severity of anxiety |  | d=0.63, medium |  |  |  | Post intervention |
|  | Disability |  | d=0.6, medium |  |  |  | Post intervention |
|  | Quality of life |  | d=0.39, medium |  |  |  | Post intervention |
| Dambi, intervention 2: The friendship-bench WhatsApp Intervention (8) | Severity of CMD | Intervention – baseline | d=0.88, large |  |  |  | Post intervention |
|  | Severity of depression |  | d=1.35, large |  |  |  | Post intervention |
|  | Severity of anxiety |  | d=1.1, large |  |  |  | Post intervention |
|  | Disability |  | d=0.91, large |  |  |  | Post intervention |
|  | Quality of life |  | d=0.06, small |  |  |  | Post intervention |
| Chibanda, The friendship-bench intervention (9) | Severity of CMD | Intervention – control (6 months post baseline) | d=1.07, large | d = 1.04, large | Baseline sociodemographic, (mental) health status |  | Post intervention |
| Ross, Telephone counselling intervention (10) | Severity of depression | Intervention – control (1 month post baseline | d=0.27, medium |  |  | Control |  |
|  |  | Intervention – control (2 months post baseline) | d=0.24, medium |  |  | Intervention |  |
| Ebrahem, Telehealth nursing intervention (11) | Proportion of people with depression (mild-severe) vs no depression | Intervention – baseline | d=0.28, medium |  |  |  | Post intervention |
|  | Proportion of people with anxiety (mild- extremely severe) vs. no anxiety |  | d=0.28, medium |  |  |  | Post intervention |
|  | Proportion of people with stress (mild-severe) vs. no stress |  | d=0.29, medium |  |  |  | Post intervention |
| Scazufca, Psychosocial intervention (12) | Severity of depression | Intervention – control (1 month post intervention) | d=1.49, large |  |  | Intervention |  |
|  | Quality of life |  | d=0.06, small |  |  | Control |  |
|  | Capability |  | d=0.17, small |  |  | Intervention |  |
| Garg, The IMproving ACcess  through Tele-psychiatry (IMPACT treatment) (13) | Severity of CMD | Intervention – baseline (3 months post baseline) | d=1.13, large |  |  |  | Post intervention |
|  | Severity of alcohol use disorder symptoms |  | d=3.22, large |  |  |  | Post intervention |
|  | Disability |  | d=0.69, medium |  |  |  | Post intervention |
| Liu, TOGETHER app (14) | Severity of depression | (Intervention – baseline) – (control – baseline) | d=0.18, small |  |  | Intervention |  |
|  |  | (Intervention – baseline) – (control – baseline) (12 weeks post intervention) | d=0.54, medium |  |  | Intervention |  |
| Öztoprak, Nurse-navigation program (15) | Quality of life | Intervention – control (10 weeks post baseline) | d=2.24, large |  |  | Intervention |  |
|  |  | Intervention – control (16 weeks post baseline) | d=3.39, large |  |  | Intervention |  |
|  | Severity of Anxiety | Intervention – control (4 weeks post baseline) | d=2.91, large |  |  | Intervention |  |
|  |  | Intervention – control (5 weeks post baseline) | d=2.5, large |  |  | Intervention |  |
|  |  | Intervention – control (10 weeks post baseline) | d=3.17, large |  |  | Intervention |  |
|  |  | Intervention – control (16 weeks post baseline) | d=3.28, large |  |  | Intervention |  |
|  | Severity of depression | Intervention – control (5 weeks post baseline) | d=1.14, large |  |  | Intervention |  |
|  |  | Intervention – control (10 weeks post baseline) | d=1.79, large |  |  | Intervention |  |
|  |  | Intervention – control (16 weeks post baseline) | d=1.86, large |  |  | Intervention |  |
| ***Digitally delivered treatment with non-specialist involvement; n=10 studies (11 interventions)*** | | | | | | | |
| Hong, The nurse-led mHealth intervention (16) | Severity of depression | (Intervention – baseline) – (control – baseline) |  | d = 0.02, medium | Baseline mental health | Intervention |  |
| Hanita, the MyEducation: CABG application (17) | Severity of depression | Intervention – control outcome (1 month post intervention) | d=1.35, large |  |  | Intervention |  |
|  | Severity of anxiety |  | d=0.2, medium |  |  | Intervention |  |
| Xu, The CARE intervention (18) | Drug positive tests | Intervention – control | d=0.66, medium |  |  | Intervention |  |
|  | Longest period of absence |  | d=0.62, medium |  |  | Intervention |  |
| Rodriguez, The MIND+ intervention (19) | Severity of depression | Intervention pre-post change outcome – control pre-post change outcome (immediately after intervention) | d=0.33, medium |  |  | Intervention |  |
|  | Severity of anxiety |  | d=0.07, small |  |  | Intervention |  |
|  | Indices of depression |  | d=0.07, small |  |  | Intervention |  |
|  | Indices of anxiety |  | d=0.1, small |  |  | Intervention |  |
|  | Indices of stress |  | d=0.09, small |  |  | Intervention |  |
|  | Mindfulness |  | d=0.19, small |  |  | Control |  |
| Anttila, intervention 1: DepisNet-Thai intervention for individual sessions (20) | Severity of depression | Intervention – control (1 month post intervention) | d=0.07, small |  |  |  | Post Intervention |
|  | Stress level |  | d=0.14, small |  |  |  | Baseline |
| Anttila, intervention 2: DepistNet-Thai active control for group sessions (20) | Severity of depression | Intervention – control (1 month post intervention) | d=0.27, medium |  |  |  | Post intervention |
|  | Stress level |  | d=0.09, small |  |  |  | Post intervention |
| Menezes, The CONEMO app intervention (21) | Proportion of people recovered from depressive | Intervention only (follow-up outcome immediately after intervention) | Brazil trial: 65%, Peru trial 1: 1.87%, Peru trial 2: 2.75% |  |  |  | Post intervention |
|  | Proportion of people with disability at baseline who had no disability at follow-up |  | Brazil trial: 10%, Peru trial 1: 6.67%, Peru trial 2: 0% |  |  |  | Post intervention |
|  | Proportion of participants without suicide risk at follow-up |  | Brazil trial: 100%, Peru trial 1 & 2: 0% |  |  |  | Post intervention |
| Zhou, The CAT intervention (22) | Level of resilience | Intervention – control |  | d = 2.68, large | Baseline mental health | Intervention |  |
| Gonsalves, POD Adventure app intervention (23) | Psychosocial problem severity | Intervention – baseline (2-3 weeks post intervention) | d=1.75, large |  |  |  | Post intervention |
|  |  | Intervention – baseline (9-10 weeks post intervention) | d=1.94, large |  |  |  | Post intervention |
|  | Mental health symptoms | Intervention – baseline (2-3 weeks post intervention) | d=0.45, medium |  |  |  | Post intervention |
|  |  | Intervention – baseline (9-10 weeks post intervention) | d=0.6, medium |  |  |  | Post intervention |
|  | Stress level | Intervention – baseline (2-3 weeks post intervention) | d=0.51, medium |  |  |  | Post intervention |
|  |  | Intervention – baseline (9-10 weeks post intervention) | d=0.6, medium |  |  |  | Post intervention |
|  | Mental well-being | Intervention – baseline (2-3 weeks post intervention) | d=0.07, small |  |  |  | Post intervention |
|  |  | Intervention – baseline (9-10 weeks post intervention) | d=0.32, medium |  |  |  | Post intervention |
| Ajradi, GAF-ID intervention (24) | Severity of depression | Intervention – control outcome (2 weeks post intervention) |  | d = 0.39, medium | Sociodemographic, baseline mental health | Intervention |  |
| Araya, The digital intervention (25) | Improvement of severity of depression | Intervention – control (3 months post intervention) |  | Brazil study: d=0.11, small; Peru study: d= 0.18, small | Brazil study: cluster (residency), Peru: cluster (health centre), both: Baseline mental health | Intervention |  |
| ***Digitally supervised non-specialists; n=3 studies (3 interventions)*** | | | | | | | |
| Khan, Group management plus intervention (26) | Psychological distress in terms of hospital anxiety and depression | Intervention – control (7 weeks post baseline |  | d=0.62, medium | Baseline mental health | Intervention |  |
|  | Psychological distress in terms of hospital anxiety |  |  | d=0.54, medium |  | Intervention |  |
|  | Psychological distress in terms of hospital depression |  |  | d=0.6, medium |  | Intervention |  |
|  | Disability |  |  | d=0.53, medium |  | Intervention |  |
|  | Psychological profile |  |  | d=0.86, large |  | Intervention |  |
|  | PTSD symptoms |  |  | d=0.15, small |  | Intervention |  |
|  | Generalised distress |  |  | d=0.15, small |  | Intervention |  |
| Rahman, Group management plus intervention (27) | Psychological distress in terms of hospital anxiety and depression | Intervention – control (1 week post intervention) |  | d=0.79, medium | Baseline mental health | Intervention |  |
|  |  | Intervention – control (3 months post intervention) |  | d=0.6, medium | Baseline mental health | Control |  |
| Chen, The COACH intervention (28) | Severity of depression | Intervention – control (3 months post baseline) | d=0.45, medium | d=0.13, small | Baseline sociodemographic, baseline (mental) health | Intervention |  |
|  |  | Intervention – control (6 months post baseline) | d=0.82, large | d=0.25, medium |  | Intervention |  |
|  |  | Intervention – control (9 months post baseline) | d=1.09, large | d=0.34, medium |  | Intervention |  |
|  |  | Intervention – control (12 months post baseline) | d=1.37, large | d=0.4, medium |  | Intervention |  |

Notes: Notes: ^1^The interpretation are based on following rules: small (d<0.2), medium (d= 0.2-0.8), large (d>0.8) (29). Abbreviations: CMD = common mental disorders

**Bibliography**

1. Rahman A, Akhtar P, Hamdani SU, et al. Using technology to scale-up training and supervision of community health workers in the psychosocial management of perinatal depression: a non-inferiority, randomized controlled trial. Glob Ment Heal. 2019; doi: 10.1017/gmh.2019.7

2. Muke SS, Tugnawat D, Joshi U, et al. Digital Training for Non-Specialist Health Workers to Deliver a Brief Psychological Treatment for Depression in Primary Care in India:Findings from a Randomized Pilot Study. Environ Res public Heal. 2020; doi: 10.3390/ijerph17176368.

3. Nisar A, Yin J, Nan Y, et al. Standardising Training of Nurses in an Evidence-Based Psychosocial Intervention for Perinatal Depression : Randomized Trial of Electronic vs . Face-to-Face Training in China. Int J Environ Res Public Heal. 2022; doi: 10.3390/ijerph19074094.

4. Pereira CA, Wen CL, Miguel EC, et al. A randomised controlled trial of a web ‑ based educational program in child mental health for schoolteachers. Eur Child Adolesc Psychiatry. 2015; doi: 10.1007/s00787-014-0642-8.

5. Maulik PK, Kallakuri S, Devarapalli S, Jha V, Patel A. Increasing use of mental health services in remote areas using mobile technology : a pre – post evaluation of the SMART Mental Health project in rural India. J Glob Health. 2017;7(1).

6. Maulik PK, Devarapalli S, Kallakuri S. The Systematic Medical Appraisal Referral and Treatment Mental Health Project : Quasi-Experimental Study to Evaluate a Technology-Enabled Mental Health Services Delivery Model Implemented in Rural India Corresponding Author : J Med Internet Res. 2020;22(e15553):1–11.

7. Doukani A, Sera F, Chibanda D. A community health volunteer delivered problem-solving therapy mobile application based on the Friendship Bench ‘ Inuka Coaching ’ in Kenya : A pilot cohort study. Glob Ment Heal. 2022;8(e9):1–11.

8. Dambi J, Norman C, Doukani A, Potgieter S, Turner J, Musesengwa R, et al. A Digital Mental Health Intervention (Inuka) for Common Mental Health Disorders in Zimbabwean Adults in Response to the COVID-19 Pandemic: Feasibility and Acceptability Pilot Study. JMIR Ment Heal. 2022;9(10): doi: https://doi.org/10.2196/37968.

9. Chibanda D, Weiss HA, Verhey R, et al. Effect of a Primary Care–Based Psychological Intervention on Symptoms of Common Mental Disorders in Zimbabwe A Randomized Clinical Trial. JAMA. 2016; doi: 10.1001/jama.2016.19102.

10. Ross R, Sawatphanit W, Suwansujarid T, et al. The Effect of Telephone Support on Depressive Symptoms Among HIV-Infected Pregnant Women in Thailand: An Embedded Mixed Methods Study. JANAC J Assoc Nurses AIDS Care. 2013; doi: 10.1016/j.jana.2012.08.005.

11. Ebrahem SM, Badawy SA, Hassan RA, et al.. Effect of Telehealth Nursing Intervention on Psychological Status and Coping Strategies Among Parents During COVID-19 Pandemic. Holist Nurs Pract. 2023; doi: 10.1097/HNP.0000000000000561.

12. Scazufca M, Clara M, Couto PDP, et al. Pilot study of a two-arm non-randomized controlled cluster trial of a psychosocial intervention to improve late life depression in socioeconomically deprived areas of São Paulo , Brazil ( PROACTIVE ): feasibility study of a psychosocial intervention for lntervention for late life depression in Sao Pãulo. BMC Public Health. 2019; doi: 10.1186/s12889-019-7495-5.

13. Garg A, Agrawal R, Velleman R, et al. Integrating assisted tele-psychiatry into primary healthcare in Goa, India: a feasibility study. Glob Ment Heal. 2022; doi: 10.1017/gmh.2021.47.

14. Liu Y, Hasimu M, Joa M, Tang J, Wang Y, He X, et al. The effect of a APP-Based Intervention for Depression Among Community-Dwelling Individuals With Spinal Cord Injury: A randomized Controlled Trial. Arch Phys Med Rehabil. 2023; doi: 10.1016/j.apmr.2022.10.005.

15. Öztoprak PU, Koç G, Erkaya S. Evaluation of the effect of a nurse navigation program developed for postpartum mothers on maternal health: A randomized controlled study. Public Health Nurs. 2023; doi: 10.1111/phn.13226.

16. Hong S, Lee S, Song K, et al. A nurse-led mHealth intervention to alleviate depressive symptoms in older adults living alone in the community: A quasi-experimental study. Int J Nurs Stud. 2023; doi: 10.1016/j.ijnurstu.2022.104431.

17. Noor Hanita Z, Khatijah LA, Kamaruzzaman S. A pilot study on development and feasibility of the ‘MyEducation: CABG application’ for patients undergoing coronary artery bypass graft (CABG) surgery. BMC Nurs. 2022; doi: 10.1186/s12912-022-00814-4.

18. Xu X, Chen S, Chen J, et al.Feasibility and Preliminary Efficacy of a Community-Based Addiction Rehabilitation Electronic System in Substance Use Disorder : Pilot Randomized Controlled Trial. JMIR mHealth uHealth. 2021; doi: 10.2196/21087.

19. Rodriguez M, Eisenlohr-moul TA, Weisman J, et al. The Use of Task Shifting to Improve Treatment Engagement in an Internet-Based Mindfulness Intervention Among Chinese University Students : Randomized Controlled Trial. JMIR Form Res. 2021; doi: 10.2196/25772.

20. Anttila M, Sittichai R, Katajisto J, et al. Impact of a Web Program to Support the Mental Wellbeing of High School Students : A Quasi Experimental Feasibility Study. Environ Res public Heal. 2019; doi: 10.3390/ijerph16142473.

21. Menezes P, Quayle J, Paulo S. Use of a Mobile Phone App to Treat Depression Comorbid With Hypertension or Diabetes : A Pilot Study in Brazil and Peru JMIR Ment Heal. 2019; doi: 10.2196/11698.

22. Zhou K, Li J, Li X. Effects of cyclic adjustment training delivered via a mobile device on psychological resilience , depression , and anxiety in Chinese post ‑ surgical breast cancer patients. Breast Cancer Res Treat. 2019; https://doi.org/10.1007/s10549-019-05368-9

23. Gonsalves PP, Hodgson ES, Bhat B, et al. App- based guided problem- solving intervention for adolescent mental health: a pilot cohort study in Indian schools. Evid Based Ment Heal. 2021; doi: 10.1136/ebmental-2020-300194.

24. Arjadi R, Nauta MH, Scholte WF, et al. Internet-based behavioural activation with lay counsellor support versus online minimal psychoeducation without support for treatment of depression : a randomised controlled trial in Indonesia. The Lancet Psychiatry. 2018; doi: 10.1016/S2215-0366(18)30223-2.

25. Araya R, Menezes PR, Claro HG, et al. Effect of a Digital Intervention on Depressive Symptoms in Patients With Comorbid Hypertension or Diabetes in Brazil and Peru Two Randomized Clinical Trials. JAMA. 2022; doi: 10.1001/jama.2021.4348.

26. Khan MN, Hamdani SU, Chiumento A, et al. Evaluating feasibility and acceptability of a group WHO trans-diagnostic intervention for women with common mental disorders in rural Pakistan: A cluster randomised controlled feasibility trial. Epidemiol Psychiatr Sci. 2019; doi: 10.1017/S2045796017000336.

27. Rahman A, Khan MN, Hamdani SU, Chiumento A, Akhtar P, Nazir H, et al. Effectiveness of a brief group psychological intervention for women in a post-conflict setting in Pakistan: a single-blind, cluster, randomised controlled trial. Lancet. 2019; doi: 10.1016/S0140-6736(18)32343-2.

28. Chen S, Conwell Y, Xue J, et al. Effectiveness of integrated care for older adults with depression and hypertension in rural China: A cluster randomized controlled trial. PLoS Med. 2022;doi: http://dx.doi.org/10.1371/journal.pmed.1004019.

29. Cohen J. statistical power analysis for behavioural sciences. 2nd ed. New York Lawrence Erlbaum Associates: New York; 1988. ISBN: 0-8058-0283-5.
