# Supplementary material for: The effectiveness of mental health interventions involving non-specialists and digital technology in low-and middle-income countries – a systematic review
Source: BMC Public Health. 2024 Jan 3;24:77. doi: 10.1186/s12889-023-17417-6 (PMC10763181; doi:10.1186/s12889-023-17417-6)
Supplement: Supplementary file 8 — Additional file 8. [file 12889_2023_17417_MOESM8_ESM.docx]

# **ADDITIONAL FILE 8: TYPE OF ANALYSIS AND LOST-TO-FOLLOW UP RATES**

**Table S8 Reasons for drop-out rates**

| **Author, reference** | **Was a ITT^1^ analysis or CCA^2^ used?** | **Total n of lost to follow-up^4^ (%)** | **Most reason for the biggest proportion of lost to follow-up** |
| --- | --- | --- | --- |
| Rahman (1) | CCA^2^ | N=20 (25%) | Temporarily absent N=10 |
| Muke (2) | CCA^2^ | N=6 (14.29%) | Unclear^5^ |
| Nisar (3) | ITT^1^ | N=11 (11%) | Unclear^5^ |
| Pereira (4) | ITT^1^ | N=61 (53.04%) | Unclear^5^ |
| Maulik (5) | ITT^2^ | N=6 (2.52%) | Died due to natural causes and not self-harm (N=3) |
| Maulik (6) | ITT^1^ | N= 57 (6.33%) | Death confirmed to be unrelated to mental illness (N=28) |
| Doukani (7) | CCA^2^ | N=8 (13.33%) | n.m. |
| Dambi (8) | CCA^2^ | N=46 (37.40%) | Connectivity issues (proportion is unclear) [might relate to intervention] |
| Ross (9) | ITT^1^ | 0 | n.a. |
| Ebrahem (10) | CCA^2^ | N=11 | n.m |
| Chibanda (11) | ITT^1^ | N= 52 (9.08%) | Moved out of city (N=19) |
| Scazufca (12) | CCA^2^ | N=4 (6.90%) | Could not be found (N=2) |
| Öztoprak (13) | CCA^2^ | N=3 (4.69%) | Moved out of province and unable to contact (N=2) |
| Garg (14) | CCA^2^ | N=48 (30%) | Could not be contacted (N=24) |
| Hong (15) | CCA^2^ | N=15 (25.42%) | n.m |
| Liu (16) | CCA^2^ | N=4 (3.92%) | Lost connection (N=3) |
| Hanita (17) | ITT^2^ | 0 | n.a. |
| Xu (18) | ITT^1^ | N=2 (5%) | Were arrested (N=2) |
| Rodriguez (19) | ITT^1^ | N=31 (57.41%) | n.m |
| Antilla (20) | CCA^2^ | N= 28 (16.77%) | n.m |
| Menezes, Sao Paolo setting (21) | CCA^2^ | N= 1 (5%) | n.m |
| Menezes, Lima 1 setting (21) |  | N= 6 (28.6%) |  |
| Menezes, Lima 2 setting (21) |  | N=8 (33%) |  |
| Zhou (22) | ITT^1^ | N= 7 (5.3%) | n.m |
| Gonsalves (23) | CCA^2^ | N=131 (52.82%) | Closure of school due to COVID (N=62) |
| Arjadi (24) | ITT^1^ | N=57 (18.21%) | No improvement (N=18) [might relate to intervention], Could not make time (N=18) |
| Araya, Brazil setting (25) | ITT^1^ | N= 90 (10.23%) | Unclear^5^ |
| Araya, Peru setting (25) | ITT^1^ | N=22 (5.09%) |  |
| Khan (26) | ITT^1^ | N=7 (5.88%) | Unclear^5^ |
| Rahman (27) | MITT^3^ | N=34 (5.56%) | Unclear^5^ |
| Chen (28) | ITT^1^ | N=152 (6.43%) | Refused (N=93) |
| Notes: Abbreviations: n.m.= not mentioned, n.a. not applicable.  Notes: ^1^Intention to treat analysis; ^2^Complete case analysis;^3^ Modified ITT defined as the analysis of participants who had at least one measurement of the primary or the secondary outcome; ^4^ Lost to follow-up was defined as those people who did not do the last assessment in the respective study. ^5^If authors do not indicate the reason for lost to follow-up or if the reason for the biggest proportion of those lost to follow-up is unclear. | | | |

**References:**

1. Rahman A, Akhtar P, Hamdani SU, et al. Using technology to scale-up training and supervision of community health workers in the psychosocial management of perinatal depression: a non-inferiority, randomized controlled trial. Glob Ment Heal. 2019; doi: 10.1017/gmh.2019.7

2. Muke SS, Tugnawat D, Joshi U, et al. Digital Training for Non-Specialist Health Workers to Deliver a Brief Psychological Treatment for Depression in Primary Care in India:Findings from a Randomized Pilot Study. Environ Res public Heal. 2020; doi: 10.3390/ijerph17176368.

3. Nisar A, Yin J, Nan Y, et al. Standardising Training of Nurses in an Evidence-Based Psychosocial Intervention for Perinatal Depression : Randomized Trial of Electronic vs . Face-to-Face Training in China. Int J Environ Res Public Heal. 2022; doi: 10.3390/ijerph19074094.

4. Pereira CA, Wen CL, Miguel EC, et al. A randomised controlled trial of a web ‑ based educational program in child mental health for schoolteachers. Eur Child Adolesc Psychiatry. 2015; doi: 10.1007/s00787-014-0642-8.

5. Maulik PK, Kallakuri S, Devarapalli S, Jha V, Patel A. Increasing use of mental health services in remote areas using mobile technology : a pre – post evaluation of the SMART Mental Health project in rural India. J Glob Health. 2017;7(1).

6. Maulik PK, Devarapalli S, Kallakuri S. The Systematic Medical Appraisal Referral and Treatment Mental Health Project : Quasi-Experimental Study to Evaluate a Technology-Enabled Mental Health Services Delivery Model Implemented in Rural India Corresponding Author : J Med Internet Res. 2020;22(e15553):1–11.

7. Doukani A, Sera F, Chibanda D. A community health volunteer delivered problem-solving therapy mobile application based on the Friendship Bench ‘ Inuka Coaching ’ in Kenya : A pilot cohort study. Glob Ment Heal. 2022;8(e9):1–11.

8. Dambi J, Norman C, Doukani A, Potgieter S, Turner J, Musesengwa R, et al. A Digital Mental Health Intervention (Inuka) for Common Mental Health Disorders in Zimbabwean Adults in Response to the COVID-19 Pandemic: Feasibility and Acceptability Pilot Study. JMIR Ment Heal. 2022;9(10): doi: https://doi.org/10.2196/37968.

9. Chibanda D, Weiss HA, Verhey R, et al. Effect of a Primary Care–Based Psychological Intervention on Symptoms of Common Mental Disorders in Zimbabwe A Randomized Clinical Trial. JAMA. 2016; doi: 10.1001/jama.2016.19102.

10. Ross R, Sawatphanit W, Suwansujarid T, et al. The Effect of Telephone Support on Depressive Symptoms Among HIV-Infected Pregnant Women in Thailand: An Embedded Mixed Methods Study. JANAC J Assoc Nurses AIDS Care. 2013; doi: 10.1016/j.jana.2012.08.005.

11. Ebrahem SM, Badawy SA, Hassan RA, et al.. Effect of Telehealth Nursing Intervention on Psychological Status and Coping Strategies Among Parents During COVID-19 Pandemic. Holist Nurs Pract. 2023; doi: 10.1097/HNP.0000000000000561.

12. Scazufca M, Clara M, Couto PDP, et al. Pilot study of a two-arm non-randomized controlled cluster trial of a psychosocial intervention to improve late life depression in socioeconomically deprived areas of São Paulo , Brazil ( PROACTIVE ): feasibility study of a psychosocial intervention for lntervention for late life depression in Sao Pãulo. BMC Public Health. 2019; doi: 10.1186/s12889-019-7495-5.

13. Garg A, Agrawal R, Velleman R, et al. Integrating assisted tele-psychiatry into primary healthcare in Goa, India: a feasibility study. Glob Ment Heal. 2022; doi: 10.1017/gmh.2021.47.

14. Liu Y, Hasimu M, Joa M, Tang J, Wang Y, He X, et al. The effect of a APP-Based Intervention for Depression Among Community-Dwelling Individuals With Spinal Cord Injury: A randomized Controlled Trial. Arch Phys Med Rehabil. 2023; doi: 10.1016/j.apmr.2022.10.005.

15. Öztoprak PU, Koç G, Erkaya S. Evaluation of the effect of a nurse navigation program developed for postpartum mothers on maternal health: A randomized controlled study. Public Health Nurs. 2023; doi: 10.1111/phn.13226.

16. Hong S, Lee S, Song K, et al. A nurse-led mHealth intervention to alleviate depressive symptoms in older adults living alone in the community: A quasi-experimental study. Int J Nurs Stud. 2023; doi: 10.1016/j.ijnurstu.2022.104431.

17. Noor Hanita Z, Khatijah LA, Kamaruzzaman S. A pilot study on development and feasibility of the ‘MyEducation: CABG application’ for patients undergoing coronary artery bypass graft (CABG) surgery. BMC Nurs. 2022; doi: 10.1186/s12912-022-00814-4.

18. Xu X, Chen S, Chen J, et al.Feasibility and Preliminary Efficacy of a Community-Based Addiction Rehabilitation Electronic System in Substance Use Disorder : Pilot Randomized Controlled Trial. JMIR mHealth uHealth. 2021; doi: 10.2196/21087.

19. Rodriguez M, Eisenlohr-moul TA, Weisman J, et al. The Use of Task Shifting to Improve Treatment Engagement in an Internet-Based Mindfulness Intervention Among Chinese University Students : Randomized Controlled Trial. JMIR Form Res. 2021; doi: 10.2196/25772.

20. Anttila M, Sittichai R, Katajisto J, et al. Impact of a Web Program to Support the Mental Wellbeing of High School Students : A Quasi Experimental Feasibility Study. Environ Res public Heal. 2019; doi: 10.3390/ijerph16142473.

21. Menezes P, Quayle J, Paulo S. Use of a Mobile Phone App to Treat Depression Comorbid With Hypertension or Diabetes : A Pilot Study in Brazil and Peru JMIR Ment Heal. 2019; doi: 10.2196/11698.

22. Zhou K, Li J, Li X. Effects of cyclic adjustment training delivered via a mobile device on psychological resilience , depression , and anxiety in Chinese post ‑ surgical breast cancer patients. Breast Cancer Res Treat. 2019; https://doi.org/10.1007/s10549-019-05368-9

23. Gonsalves PP, Hodgson ES, Bhat B, et al. App- based guided problem- solving intervention for adolescent mental health: a pilot cohort study in Indian schools. Evid Based Ment Heal. 2021; doi: 10.1136/ebmental-2020-300194.

24. Arjadi R, Nauta MH, Scholte WF, et al. Internet-based behavioural activation with lay counsellor support versus online minimal psychoeducation without support for treatment of depression : a randomised controlled trial in Indonesia. The Lancet Psychiatry. 2018; doi: 10.1016/S2215-0366(18)30223-2.

25. Araya R, Menezes PR, Claro HG, et al. Effect of a Digital Intervention on Depressive Symptoms in Patients With Comorbid Hypertension or Diabetes in Brazil and Peru Two Randomized Clinical Trials. JAMA. 2022; doi: 10.1001/jama.2021.4348.

26. Khan MN, Hamdani SU, Chiumento A, et al. Evaluating feasibility and acceptability of a group WHO trans-diagnostic intervention for women with common mental disorders in rural Pakistan: A cluster randomised controlled feasibility trial. Epidemiol Psychiatr Sci. 2019; doi: 10.1017/S2045796017000336.

27. Rahman A, Khan MN, Hamdani SU, Chiumento A, Akhtar P, Nazir H, et al. Effectiveness of a brief group psychological intervention for women in a post-conflict setting in Pakistan: a single-blind, cluster, randomised controlled trial. Lancet. 2019; doi: 10.1016/S0140-6736(18)32343-2.

28. Chen S, Conwell Y, Xue J, et al. Effectiveness of integrated care for older adults with depression and hypertension in rural China: A cluster randomized controlled trial. PLoS Med. 2022;doi: http://dx.doi.org/10.1371/journal.pmed.1004019
