# Supplementary material for: The effectiveness of mental health interventions involving non-specialists and digital technology in low-and middle-income countries – a systematic review
Source: BMC Public Health. 2024 Jan 3;24:77. doi: 10.1186/s12889-023-17417-6 (PMC10763181; doi:10.1186/s12889-023-17417-6)
Supplement: Supplementary file 9 — Additional file 9. [file 12889_2023_17417_MOESM9_ESM.docx]

# **ADDITIONAL FILE 9: EXCLUSION CRITERIA FOR RECRUITMENT OF PARTICIPANTS IN EACH STUDY**

**Table S9. Summary of exclusion criteria for participants in each study**

| **Study reference** | **Exclusion criteria** |
| --- | --- |
| Rahman (1) | n.m^1^ |
| Muke (2) | Participation in previous research activities by the study research team, significant speech, sight, or hearing impairment, illiteracy |
| Nisar (3) | n.m^1^ |
| Pereira (4) | n.m^1^ |
| Maulik (5) | Limited by any severe physical disorder in accessing mental health services |
| Maulik (6) | Severe depression (a score of ≥15 on either the PHQ-9^2^ and/or GAD-7^3^), increased suicide risk (a score ≥1 on the self-harm–related question of PHQ-9^2^) |
| Doukani (7) | n.m^1^ |
| Dambi (8) | Receiving specialist mental health care at the point of recruitment, known psychiatric conditions (i.e., major depression). |
| Chibanda (9) | Inability to understand the study procedures, inability to speak English or Shona, suicidal intent, end-stage AIDS, currently in psychiatric care, pregnant or up to 3 months’ postpartum, or current psychosis, intoxication, and/or dementia |
| Ross (10) | n.m^1^ |
| Ebrahem (11) | n.m^1^ |
| Scazufca (12) | PHQ-9^2^ < 10, complete deafness, terminal illness, risk of suicide, inability to communicate (e.g., due to cognitive impairment either reported by a family member or detected by the researcher) |
| Öztoprak (13) | People who withdrew at any  phase of the study, those moving out of the study location during the study period, and those who developed (or had a baby who developed) any complications in during the study period |
| Garg (14) | Patients requiring immediate medical assistance, not planning to stay in the study area the upcoming 6 months, unable to speak English, Hindi, Kokani, or Marathi. |
| Liu (16) | Spinal cord injury (SCI) caused by congenital disease, individuals with serious dysfunction of the heart, brain, lung, liver or kidney, currently participating in other rehabilitation or nursing projects related to SCI |
| Hong (15) | Significant cognitive impairment, high risk of suicide (based on Korean versions of the Mini-Mental Status Examination, Crisis Tirage Ration Scale and Scale for Suicidal Ideation), emergency hospitalisation due to severe depression. |
| Hanita (17) | Patients undergoing emergency surgery, with prior valve surgery, signs of neurological or cognitive impairments including vision, hearing or any impairment impeding their ability to use a digital device. |
| Xu, 2021 (18) | Inability or refusal to use smartphone app, severe cognitive impairment, or a history  of suicidality. |
| Rodriguez (19) | Aged <18 years, no proof of current student status and emergency contact, currently experiences manic or psychotic symptoms, expresses suicidal or homicidal ideation during the intake phone interview |
| Anttila (20) | Inadequate Thai skills, inability to provide informed consent |
| Menezes (21) | Psychosis symptoms (Psychosis Screening Questionnaire), pregnancy (self-reported), high suicidal risk (positive answer in item 9 of the PHQ-9^2^, protocol to assess suicidal risk  (Suicide Risk Assessment Protocol), cognitive impairment (Community Screening for Dementia). |
| Zhou (22) | Presence of cognitive and psychiatric disorders (screened and diagnosed by a psychiatrist not involved in this study and the DSM-5^4^), other malignant tumors, and breast disease. |
| Gonsalves (23) | Elevated risk of self-harm or suicide requiring external referral |
| Arjadi (24) | Current substance use disorder (SCID-5^5^), current or previous manic or hypomanic episodes or psychotic disorder, and acute  suicidality (defined as a suicide plan with preparatory behaviour), currently being seen at least weekly for psychological interventions |
| Araya (25) | High suicide risk, pregnant women with gestational diabetes and/or hypertension at screening stage |
| Khan (26) | Suicide risk, cognitive impairment (e.g., severe intellectual disability or dementia) or mental disorder (psychotic disorders, substance-dependence) |
| Rahman (27) | Severe mental disorder (i.e., psychotic disorder, substance abuse disorder), severe cognitive impairment and severe intellectual disabilities |
| Chen (28) | Mania, psychosis, alcohol abuse/dependency within the past 6 months from baseline (based on the Mini-International Neuropsychiatric Interview), acute suicide risk (evaluated by participants primary care physician) |
| Notes: ^1^ not mentioned;^2^Patient Health Questionnaire-9;^3^ Generalized Anxiety Disorder 7;^4^ Diagnostic and Statistical Manual of Mental Disorders;^5^ Structured Clinical Interview for DSM-5 | |

**References:**

1. Rahman A, Akhtar P, Hamdani SU, et al. Using technology to scale-up training and supervision of community health workers in the psychosocial management of perinatal depression: a non-inferiority, randomized controlled trial. Glob Ment Heal. 2019; doi: 10.1017/gmh.2019.7

2. Muke SS, Tugnawat D, Joshi U, et al. Digital Training for Non-Specialist Health Workers to Deliver a Brief Psychological Treatment for Depression in Primary Care in India:Findings from a Randomized Pilot Study. Environ Res public Heal. 2020; doi: 10.3390/ijerph17176368.

3. Nisar A, Yin J, Nan Y, et al. Standardising Training of Nurses in an Evidence-Based Psychosocial Intervention for Perinatal Depression : Randomized Trial of Electronic vs . Face-to-Face Training in China. Int J Environ Res Public Heal. 2022; doi: 10.3390/ijerph19074094.

4. Pereira CA, Wen CL, Miguel EC, et al. A randomised controlled trial of a web ‑ based educational program in child mental health for schoolteachers. Eur Child Adolesc Psychiatry. 2015; doi: 10.1007/s00787-014-0642-8.

5. Maulik PK, Kallakuri S, Devarapalli S, Jha V, Patel A. Increasing use of mental health services in remote areas using mobile technology : a pre – post evaluation of the SMART Mental Health project in rural India. J Glob Health. 2017;7(1).

6. Maulik PK, Devarapalli S, Kallakuri S. The Systematic Medical Appraisal Referral and Treatment Mental Health Project : Quasi-Experimental Study to Evaluate a Technology-Enabled Mental Health Services Delivery Model Implemented in Rural India Corresponding Author : J Med Internet Res. 2020;22(e15553):1–11.

7. Doukani A, Sera F, Chibanda D. A community health volunteer delivered problem-solving therapy mobile application based on the Friendship Bench ‘ Inuka Coaching ’ in Kenya : A pilot cohort study. Glob Ment Heal. 2022;8(e9):1–11.

8. Dambi J, Norman C, Doukani A, Potgieter S, Turner J, Musesengwa R, et al. A Digital Mental Health Intervention (Inuka) for Common Mental Health Disorders in Zimbabwean Adults in Response to the COVID-19 Pandemic: Feasibility and Acceptability Pilot Study. JMIR Ment Heal. 2022;9(10): doi: https://doi.org/10.2196/37968.

9. Chibanda D, Weiss HA, Verhey R, et al. Effect of a Primary Care–Based Psychological Intervention on Symptoms of Common Mental Disorders in Zimbabwe A Randomized Clinical Trial. JAMA. 2016; doi: 10.1001/jama.2016.19102.

10. Ross R, Sawatphanit W, Suwansujarid T, et al. The Effect of Telephone Support on Depressive Symptoms Among HIV-Infected Pregnant Women in Thailand: An Embedded Mixed Methods Study. JANAC J Assoc Nurses AIDS Care. 2013; doi: 10.1016/j.jana.2012.08.005.

11. Ebrahem SM, Badawy SA, Hassan RA, et al.. Effect of Telehealth Nursing Intervention on Psychological Status and Coping Strategies Among Parents During COVID-19 Pandemic. Holist Nurs Pract. 2023; doi: 10.1097/HNP.0000000000000561.

12. Scazufca M, Clara M, Couto PDP, et al. Pilot study of a two-arm non-randomized controlled cluster trial of a psychosocial intervention to improve late life depression in socioeconomically deprived areas of São Paulo , Brazil ( PROACTIVE ): feasibility study of a psychosocial intervention for lntervention for late life depression in Sao Pãulo. BMC Public Health. 2019; doi: 10.1186/s12889-019-7495-5.

13. Garg A, Agrawal R, Velleman R, et al. Integrating assisted tele-psychiatry into primary healthcare in Goa, India: a feasibility study. Glob Ment Heal. 2022; doi: 10.1017/gmh.2021.47.

14. Liu Y, Hasimu M, Joa M, Tang J, Wang Y, He X, et al. The effect of a APP-Based Intervention for Depression Among Community-Dwelling Individuals With Spinal Cord Injury: A randomized Controlled Trial. Arch Phys Med Rehabil. 2023; doi: 10.1016/j.apmr.2022.10.005.

15. Öztoprak PU, Koç G, Erkaya S. Evaluation of the effect of a nurse navigation program developed for postpartum mothers on maternal health: A randomized controlled study. Public Health Nurs. 2023; doi: 10.1111/phn.13226.

16. Hong S, Lee S, Song K, et al. A nurse-led mHealth intervention to alleviate depressive symptoms in older adults living alone in the community: A quasi-experimental study. Int J Nurs Stud. 2023; doi: 10.1016/j.ijnurstu.2022.104431.

17. Noor Hanita Z, Khatijah LA, Kamaruzzaman S. A pilot study on development and feasibility of the ‘MyEducation: CABG application’ for patients undergoing coronary artery bypass graft (CABG) surgery. BMC Nurs. 2022; doi: 10.1186/s12912-022-00814-4.

18. Xu X, Chen S, Chen J, et al.Feasibility and Preliminary Efficacy of a Community-Based Addiction Rehabilitation Electronic System in Substance Use Disorder : Pilot Randomized Controlled Trial. JMIR mHealth uHealth. 2021; doi: 10.2196/21087.

19. Rodriguez M, Eisenlohr-moul TA, Weisman J, et al. The Use of Task Shifting to Improve Treatment Engagement in an Internet-Based Mindfulness Intervention Among Chinese University Students : Randomized Controlled Trial. JMIR Form Res. 2021; doi: 10.2196/25772.

20. Anttila M, Sittichai R, Katajisto J, et al. Impact of a Web Program to Support the Mental Wellbeing of High School Students : A Quasi Experimental Feasibility Study. Environ Res public Heal. 2019; doi: 10.3390/ijerph16142473.

21. Menezes P, Quayle J, Paulo S. Use of a Mobile Phone App to Treat Depression Comorbid With Hypertension or Diabetes : A Pilot Study in Brazil and Peru JMIR Ment Heal. 2019; doi: 10.2196/11698.

22. Zhou K, Li J, Li X. Effects of cyclic adjustment training delivered via a mobile device on psychological resilience , depression , and anxiety in Chinese post ‑ surgical breast cancer patients. Breast Cancer Res Treat. 2019; https://doi.org/10.1007/s10549-019-05368-9

23. Gonsalves PP, Hodgson ES, Bhat B, et al. App- based guided problem- solving intervention for adolescent mental health: a pilot cohort study in Indian schools. Evid Based Ment Heal. 2021; doi: 10.1136/ebmental-2020-300194.

24. Arjadi R, Nauta MH, Scholte WF, et al. Internet-based behavioural activation with lay counsellor support versus online minimal psychoeducation without support for treatment of depression : a randomised controlled trial in Indonesia. The Lancet Psychiatry. 2018; doi: 10.1016/S2215-0366(18)30223-2.

25. Araya R, Menezes PR, Claro HG, et al. Effect of a Digital Intervention on Depressive Symptoms in Patients With Comorbid Hypertension or Diabetes in Brazil and Peru Two Randomized Clinical Trials. JAMA. 2022; doi: 10.1001/jama.2021.4348.

26. Khan MN, Hamdani SU, Chiumento A, et al. Evaluating feasibility and acceptability of a group WHO trans-diagnostic intervention for women with common mental disorders in rural Pakistan: A cluster randomised controlled feasibility trial. Epidemiol Psychiatr Sci. 2019; doi: 10.1017/S2045796017000336.

27. Rahman A, Khan MN, Hamdani SU, Chiumento A, Akhtar P, Nazir H, et al. Effectiveness of a brief group psychological intervention for women in a post-conflict setting in Pakistan: a single-blind, cluster, randomised controlled trial. Lancet. 2019; doi: 10.1016/S0140-6736(18)32343-2.

28. Chen S, Conwell Y, Xue J, et al. Effectiveness of integrated care for older adults with depression and hypertension in rural China: A cluster randomized controlled trial. PLoS Med. 2022;doi: http://dx.doi.org/10.1371/journal.pmed.1004019
