# Supplementary material for: The effectiveness of mental health interventions involving non-specialists and digital technology in low-and middle-income countries – a systematic review
Source: BMC Public Health. 2024 Jan 3;24:77. doi: 10.1186/s12889-023-17417-6 (PMC10763181; doi:10.1186/s12889-023-17417-6)
Supplement: Supplementary file 10 — Additional file 10. [file 12889_2023_17417_MOESM10_ESM.docx]

# **ADDITIONAL FILE 10: CLINICAL FUNCTIONS OF DIGITAL TECHNOLOGY & NON-SPECIALIST INTERVENTIONS**

**Rationale for differentiating studies into following groups:**

1. Digital training
2. Digital support for non-specialist delivered intervention
3. Digitally delivered interventions with non-specialist involvement
4. Digital supervision

Table S10.1. shows each function of the digital technology in non-specialist mental healthcare interventions. Following definitions could be derived.

***Definitions of clinical functions:***

**Organizational tasks**

*Data collection*

Technology is used to store patient-related data, so that the non-specialist or other relevant health workers (i.e. doctors, or clinical managers) can monitor them.

*Alerts and reminders*

Digital applications were used to alert the service-receiver, the non-specialists and/or relevant stakeholders (i.e., clinical manager or supervisors) on aggravation of symptoms or non-adherence of patients. Additionally, text-messages were used to remind patients on follow-up treatments, in case of non-adherence.

*Care coordination*

Digital applications were used by the non-specialists to monitor appointments, or to encourage treatment adherence. In the latter example, asynchronous or synchronous communication techniques were used to increase adherence rates by pre-planning future treatment appointments or encouraging the participants.

**Guidance in intervention delivery**

*Decision support*

Digital support systems were used by non-specialists who provided the care (face-to-face or remotely). In particular, decision support systems were used to screen or diagnose participants based on evidence-based manuals or to provide tailored treatment to the patients.

*Visualization tool for mental health education purposes*

Usage of informational videos for educational outreach interventions.

**Connection between health worker and service receiver**

*Emergency contact*

Digital technology was used to connect patient with carers and health workers in case of an emergency.

*Communication*

Technology was used for communication between intervention provider and service user. For face-to-face delivered treatments, asynchronous communication via text-messages can be used to reinforce the acquired treatment skills from the previous session. For interventions in which the non-specialists primarily provide the treatment remotely, technology was used for synchronous or for asynchronous treatment-delivery. In cases where the digital app or website was primarily used to provide the treatment, the users could communicate asynchronously or synchronously to receive further support on the treatment content and/or resolve technical issues.

**Main deliverer of treatment components**

*Provides treatment for specific groups of disorders*

The digital technology provides (components of) the therapy (i.e., education, exercises) via a digital application or website that needs to be completed by the service user.

**Supervision**

*Supervision*

A digital tool is used to enable supervision of non-specialists by specialists (i.e., trough phone or video calls).

**Explanation of categorization of studies according to the role of the digital technology:**

Digital training: In this domain the study focusses on the non-specialist who receives a digital training to provide any form of mental health intervention

Digital support for the non-specialist: In this domain the non-specialist is the main deliverer of the intervention. The non-specialist delivers the intervention (i.e., treatment or education etc.) using digital tools as a further support (i.e., app or website). Intervention could be delivered in person, or remotely (asynchronous or synchronous) by the non-specialist.

Digitally-delivered interventions with non-specialist involvement: In this domain the digital technology is the main provider of the intervention and the non-specialist acts as further support. In particular, the non-specialist supports in terms of technical assistance, discussions on the resolved tasks, supporting motivation and adherence or provide specific treatment components alongside the main digital intervention.

Digital supervision: In this domain the non-specialists who provides any mental health intervention receives digital supervision.

**Table S10.1 Clinical functions of digital technologies in each intervention**

| **Author, date** | **Type of non-specialist** | **Training & competence building of non-specialists** | **Data collection & reporting** | **Decision support tools (i.e, for screening or treatment delivery)** | **Alerts and reminders** | **Emergency contact** | **Visualization tool for mental health education purposes** | **Provides treatment for specific groups of disorders (exercises & information)** | **Communication between non-specialist and user** | **Care coordination** | **Supervision of non-specialists** |
| --- | --- | --- | --- | --- | --- | --- | --- | --- | --- | --- | --- |
| Rahman, 2019 (1) | Lady health workers | X |  |  |  |  |  |  |  |  |  |
| Muke, 2020 (DGT) (2) | ASHA^1^, ASHA^1^ Facilitators, Multi-Purpose Health Workers | X |  |  |  |  |  |  |  |  |  |
| Muke, 2020  (DGT+) (2) | ASHA^1^, ASHA^1^ Facilitators, Multi-Purpose Health Workers | X |  |  |  |  |  |  |  |  |  |
| Nisar, 2022 (3) | Nurses | X |  |  |  |  |  |  |  |  |  |
| Pereira (WBIE),2015 (4) | Teachers | X |  |  |  |  |  |  |  |  |  |
| Pereira (TVBE), 2015 (4) | Teachers | X |  |  |  |  |  |  |  |  |  |
| Maulik, 2017 (5) | ASHA^1^, primary care physician |  | X | X |  |  | X |  |  |  |  |
| Maulik, 2020 (6) | ASHA^1^, primary care physician |  | X | X |  |  | X |  |  |  |  |
| Doukani, 2021 (7) | Community health volunteer^2^ |  | X | X |  |  |  |  | X |  |  |
| Dambi (8) | Community health volunteers^2^ |  | X | X |  |  |  |  | X | X |  |
| Dambi (8) | Community health volunteers^2^ |  | X | X |  |  |  |  | X | X |  |
| Chibanda, 2016 (9) | Lay health worker^2^ |  | X |  | X |  |  |  |  | X |  |
| Ross, 2013 (10) | Nurses |  |  |  |  |  |  |  | X |  |  |
| Ebrahem, 2023 (11) |  |  |  |  |  |  |  |  | X |  |  |
| Scazufca, 2019 (12) | Nurse assistant or community health worker |  | X | X | X |  |  |  |  | X | X |
| Garg, 2022 (13) | People with at least senior secondary level education + experience in other task-shifting care delivery |  | X |  |  |  |  |  | X | X |  |
| Liu, 2023 (14) | Collaborative nursing team |  |  | X |  |  |  |  | X | X |  |
| Öztoprak, 2023 (15) | Nurse |  |  |  | X |  |  |  | X |  |  |
| Hong, 2023 (16) | Nurses & community nurses |  |  |  |  |  |  | X |  |  |  |
| Hanita, 2022 (17) | Nurses |  |  |  | X |  |  | X |  |  |  |
| Xu, 2021 (18) | Social worker |  | X |  |  | X |  | X | X |  |  |
| Rodriguez, 2021 (19) | University students |  |  |  |  |  |  | X | X |  | X |
| Anttila, 2019 (intervention group) (20) | Teachers |  |  |  |  |  |  | X |  |  |  |
| Anttila 2019 (active control group) (20) | Teachers |  |  |  |  |  |  | X |  |  |  |
| Menezes, 2019 (21) | Nurse or nurse assistant |  |  |  |  |  |  | X | X |  |  |
| Zhou, 2019 (22) | Nurses |  | X |  |  |  |  | X | X |  |  |
| Gonsalves, 2021 (23) | College graduates with experience in delivering the problem-solving intervention |  |  |  |  |  |  | X |  |  |  |
| Arjadi, 2018 (24) | Lay counsellors (not further specified) |  |  |  | X |  |  | X | X |  |  |
| Araya, 2021 (25) | Nurse assistant |  |  |  | X |  |  | X | X | X |  |
| Khan, 2019 (26) | Lay helpers (not further specified), lady health workers |  |  |  |  |  |  |  |  |  | X |
| Rahman, 2019 (27) | Facilitators (not further specified), lady health workers |  |  |  |  |  |  |  |  |  | X |
| Chen, 2022 (28) | Aging workers |  |  |  |  |  |  |  |  |  | X |
| Notes: ^1^ASHA= Accredited Social Health Activist, ^2^ it is not further specified which background (occupational, educational or societal) these non-specialists have. | | | | | | | | | | | |

**Type of non-specialist intervention**

These differentiation of non-specialist intervention types is based on the frameworks provided by Barnett et al. (29,30).

Outreach interventions: In these interventions the non-specialist is responsible to bridge the gap between the community and the formal healthcare system. The non-specialist provides for example mental illness screening or mental health education or promotion within a healthy population.

Auxiliary care interventions: In these interventions the non-specialists assist the specialist who provides the psychotherapy treatment, by supporting in care management tasks or supporting the motivation and adherence of the service-receiver.

Stepped care intervention: Stepped-care interventions involve a collaborative approach where non-specialists initially offer low-intensity psychosocial interventions (i.e., breathing exercises, sports etc.). If there is no noticeable progress, a specialist steps in to provide more intensive psychotherapy. These interventions typically include a specialist Mental Health Care (MHC) worker in the care process.

Primary-delivery interventions: Primary-delivery interventions rely on non-specialists to independently deliver the components of psychotherapy treatment during the study period. The specialist's role is limited to training and supervising the non-specialist and addressing individuals with adverse effects, such as those with suicidal intentions. These interventions are particularly useful in remote areas where specialized professionals are scarce.

**Table S10.2 The role of non-specialists and type of delivered intervention**

| **Study** | **The role of the non-specialist** | **Type of intervention delivered** | **Justification** |
| --- | --- | --- | --- |
| Digital training with face-to-face support (1) | Receives digital training to deliver perinatal depression treatment | Primary-delivery approach | The non-specialist delivers the thinking healthy program, a CBT-based intervention to treat perinatal depression. The specialist is only involved in training and supervision. Hence, because the non-specialist provides psychotherapy components and the specialist is not directly part of care-delivery, this type is considered as the primary-delivery intervention. |
| Digital training with remote support (DGT+) (2) | Receives digital training to deliver depression treatment | Primary-delivery approach | The non-specialist is trained to deliver the Healthy Activity Program (HAP), which is a treatment for depression based on problem-solving therapy. The specialist is only involved in training and supervision. Referral to specialist-provided care was not part of the intervention (considered as discharge). Hence, because the non-specialist provides psychotherapy components and the specialist is not directly part of care-delivery, this type is considered as the primary-delivery intervention. |
| Digital training without remote support (DGT) (2) | Receives digital training to deliver depression treatment | Primary-delivery approach | The non-specialist is trained to deliver the Healthy Activity Program (HAP), which is a treatment for depression based on problem-solving therapy. The specialist is only involved in training and supervision. Referral to specialist-provided care was not part of the intervention (considered as discharge). Hence, because the non-specialist provides psychotherapy components and the specialist is not directly part of care-delivery, this type is considered as the primary-delivery intervention. |
| Digital training with face-to-face support (3) | Receives digital training to deliver perinatal depression treatment | Primary-delivery approach | The non-specialist is trained to deliver the Healthy Activity Program (HAP), which is a treatment for depression based on problem-solving therapy. The specialist is only involved in training and supervision. Referral to specialist-provided care was not part of the intervention (considered as discharge). Hence, because the non-specialist provides psychotherapy components and the specialist is not directly part of care-delivery, this type is considered as the primary-delivery intervention. |
| Web-based interactive education intervention (4) | Receives digital training to educate on identifying and handling child mental disorders | Outreach | The non-specialist is trained to provide mental health education in schools + identification and referral of those with mental problems. Hence the non-specialist provides outreach work. |
| Text-and video-based education (4) | Receives video-based training to educate on identifying and handling child mental disorders | Outreach | The non-specialist is trained to provide mental health education in schools + identification and referral of those with mental problems. Hence the non-specialist provides outreach work. |
| The SMART intervention (5) | Health worker: Screens population for common mental disorders through app; physician: diagnosis and treatment through app | Outreach (screening) + primary delivery approach | Health workers provides screening (outreach) and physician provides treatment based on mhGap manuals. The specialist only takes up severe cases (patients with drug abuse or psychotic symptoms) but was not directly involved in this intervention. Hence this type is a combination of outreach + primary-delivery treatment intervention. |
| The SMART intervention (6) | Health worker: Screens population for common mental disorders through app; physician: diagnosis and treatment through app | Outreach (screening) + primary delivery approach | Health workers provides screening (outreach) and physician provides treatment based on mhGap manuals. The specialist only takes up severe cases (patients with drug abuse or psychotic symptoms) but was not directly involved in this intervention. Hence this type is a combination of outreach + primary-delivery treatment intervention. |
| Inouka coaching app intervention (7) | Delivers low-intensity problem-solving therapy through app (via chat-function) | Primary delivery approach | The non-specialist provides problem-solving therapy for people with CMD. If after the intervention no progress can be detected, the patient will be referred to a specialist. This would mean that the specialist is not directly part of the intervention. Hence, we consider this as the primary-delivery model. |
| Inouka coaching app intervention (8) | Delivers low-intensity problem-solving therapy through app (via chat-function) | Primary delivery approach | The non-specialist provides problem-solving therapy for people with CMD is delivered. If after the intervention no progress can be detected, the patient will be referred to a specialist. This would mean that the specialist is not directly part of the intervention. Hence, we consider this as primary delivery model. |
| Friendship-bench whatsapp intervention (8) | Delivers low-intensity problem-solving therapy through app (via chat-function) | Primary delivery approach | The non-specialist provides problem-solving therapy for people with CMD is delivered. If after the intervention no progress can be detected, the patient will be referred to a specialist. This would mean that the specialist is not directly part of the intervention. Hence, we consider this as the primary delivery model. |
| The friendship-bench intervention (9) | Provides problem solving therapy to patients face-to-face and supports therapy adherence through phone calls/SMS | Primary delivery approach | The non-specialist provides problem-solving therapy for people with CMD is delivered. If after the intervention no progress can be detected, the patient will be referred to a specialist. This would mean that the specialist is not directly part of the intervention. Hence, we consider this as the primary delivery model. |
| Telephone counselling intervention (10) | Provides emotional and informational support for pregnant women with HIV through phone | Primary delivery approach | The non-specialist provides emotional support. Because the non-specialist does not deliver a specific evidence-based psychological treatment intervention and because a referral to a specialist is not included in this intervention, we consider this as the primary-delivery model. |
| Telehealth nursing intervention (11) | Provides COVID-related information and adaptive coping strategies | Primary delivery approach | The non-specialist provides adaptive coping strategies including deep-breathing exercise, listening to music, getting support from friends, and healthy ways for expressing  the negative feeling of themselves and their children such as drawing. Because the non-specialist does not deliver a specific evidence-based psychological treatment intervention and because a referral to a specialist is not included in this intervention, we consider this as the primary-delivery model. |
| PROACTIVE intervention (12) | Delivers psychosocial treatment based on behavioural activation therapy techniques through in-person meeting at home + phone calls | Primary delivery approach | The non-specialist provides low-and high intensity treatment depending on the severity of symptoms based on the evidence-based behavioural activation therapy components. Because the non-specialist provides both treatment types, and the specialist (a psychologist) is responsible for supervision only, and not in direct care delivery, we consider this as the primary delivery model. |
| The IMPACT treatment (13) | Delivers assisted tele-psychiatric treatment | Auxiliary care | Treatment is provided by the psychiatrist and the non-specialist assists the specialists through care coordination, assessment and monitoring of (mental) health status, liaising with family members of service receiver, providing additional counselling. Hence this type of care is considered as an auxiliary care model. |
| Together intervention (14) | Provides the mHealth (TOGETHER) app to people with spinal cord injury | Stepped-care | The non-specialist provides online sessions with the patient using the mHealth app at follow-up after hospital discharge. The non-specialist first provides care alone. In particular, the non-specialist provides tailored health education, which we consider as a low-level psychosocial intervention task, including assessing the severity of negative emotions, listening to patients’ thoughts, and encouraging and comforting them. If required (i.e., when psychological symptoms do not improve) the non-specialist refers the patient to specialist, hence we consider this as the stepped-care approach. |
| The nurse-navigation program (15) | Provides post-natal care including psychological components | Primary delivery approach | The non-specialist provides post-natal care including psychosocial care. Because there is no specialist involved in care delivery, this type is considered as the primary delivery model. |
| Nurse-led mHealth intervention (16) | Delivers mHealth based depression treatment | Auxiliary care | The non-specialist supports in the digital intervention by guiding the care-receiver + additional consultation on intervention content and technical problems, and supporting adherence. Specifically, the app provided cognitive behavioural therapy components such as teaching problem-solving skills, relaxation techniques, and practising social skills that address depression, online/offline art activities. The specialist (a trained art therapist) provides group-therapy sessions. Hence, we consider this type as an intervention where the non-specialist assists the specialist who provides the specialised care; hence this type is considered as an auxiliary care model. |
| MyEducation: CABG application (17) | Implements the educational health applications + is alerted in case of problems | Primary delivery approach | The non-specialist supports in the digital intervention by guiding the care receiver and providing additional support (symptom management) in case of emergencies. There is no mental health specialist involved. Hence, this intervention is considered as the primary delivery model. |
| The CARE intervention (18) | Provides care as usual (weekly urine screen, application for social benefits), using the CARE website to obtain health-related information, track location of participant, track and support adherence to treatment, and supports motivation. | Primary delivery approach | The non-specialist supports the care-receiver who receives a digital drug-rehabilitation care. There is no specialist involved in this rehabilitation care. Hence, we consider this as the primary delivery model. |
| The MIND+ intervention (19) | Encourages and supports treatment adherence. | Primary-delivery approach | The non-specialist supports the care-receiver who receives a digital mindfulness treatment. There is no specialist involved in this intervention. Hence, the non-specialist acts as the primary delivery model. |
| DepisNet-Thai intervention for individual sessions (20) | Group sessions with teachers who work together with tasks provided by the app/website | Outreach: mental health promotion | The non-specialist supports the service-receiver who receives a digital mental health promotion (wellbeing) intervention. Hence, the non-specialist provides an outreach intervention. |
| DepistNet-Thai active control for group sessions (20) | Run the program, act as tutor, guide students on answering the questions in the app, read students excercise, interact in discussions, checking & supporting adherence | Outreach: mental health promotion | The non-specialist supports the service-receiver who receives a digital mental health promotion (wellbeing) intervention. Hence, the non-specialist provides an outreach intervention. |
| The CONEMO app intervention (21) | Introduces the app, closes the final session.  Resolves questions related to the app, supports adherence and motivation. | Primary delivery approach | The non-specialist supports the service receiver who receives a digital psychoeducation and behavioural activation treatment. The specialist (a psychologist) is only responsible for supervision and not involved in care provision. Hence, we consider this intervention as the primary delivery model. |
| The CAT intervention (22) | Provides routine nursing care + provides part of the CAT intervention based on the Roy Adaptation Model (RAM). | Primary delivery approach | The non-specialist is part of a digitally-delivered intervention (Cyclic Adjustment Training). The non-specialist supports the user in fulfilling the tasks + provides psychosocial treatment components, such as relaxation techniques, encouraging social behaviour (meeting with friends), encouraging and promoting positive feelings. Because there are no specialists involved in care delivery, this intervention is considered as the primary delivery model. |
| POD Adventure app intervention (23) | Introduces the app, guides and supports the users in technical or content issues. | Primary delivery approach | The non-specialist supports the service receiver who receives a digital problem-solving therapy. The specialist (psychologist) is only responsible for supervision and not involved in care delivery. Hence, this intervention is considered as the primary delivery model |
| GAF-ID intervention (24) | Introduces the web-program, supports and provides feedback on completed modules, technical assistance, supports adherence. | Primary delivery approach | The non-specialist supports the service receiver who receives psychoeducation and behavioural activation therapy. The specialist (psychologist) is only responsible for supervision and not directly involved in care delivery, except for acute cases (suicidality). Hence, this intervention is considered as the primary delivery model. |
| The digital intervention (25) | Provided support in app usage by supporting adherence, motivation and resolving technical problems | Primary delivery approach | The non-specialist supports the service receiver who receives psychoeducation and behavioural activation therapy. The specialist (psychologist) was only responsible for supervision and not directly involved in care provision. Hence, this intervention is considered as the primary delivery model. |
| Group management plus intervention (26) | Provides treatment and receives digital supervision via skype | Primary delivery approach | The non-specialist provides treatment based on problem-solving therapy, counselling and behavioural techniques. The specialist was not directly involved in treatment delivery but only for training and supervision of non-specialist. Hence, this intervention is considered as the primary delivery model. |
| Group management plus intervention (27) | Provides treatment and receives digital supervision via skype | Primary delivery approach | The non-specialist provides treatment based on problem-solving therapy, counselling and behavioural techniques. The specialist was not directly involved in treatment delivery but only for training and supervision of non-specialist. Hence, this intervention is considered as the primary delivery model. |
| The COACH intervention (28) | Provides part of the treatment and receives supervision via phone | Outreach + primary-delivery | The non-specialists provide different part of the treatment and educational work in the community (outreach).  One non-specialist (aging worker) provides psychoeducation (outreach work), while the other non-specialist (primary care physician) provides treatment using evidence-based toolkits and guidelines. The specialist (psychiatrist) meets with the patient once for a counselling session + medication prescription as needed. However, except from this task, this specialist is not involved in treatment delivery and is only responsible for supervision purposes. Hence, we consider this type as a combination of outreach + primary-delivery treatment model. |

**References:**

1. Rahman A, Akhtar P, Hamdani SU, et al. Using technology to scale-up training and supervision of community health workers in the psychosocial management of perinatal depression: a non-inferiority, randomized controlled trial. Glob Ment Heal. 2019; doi: 10.1017/gmh.2019.7

2. Muke SS, Tugnawat D, Joshi U, et al. Digital Training for Non-Specialist Health Workers to Deliver a Brief Psychological Treatment for Depression in Primary Care in India:Findings from a Randomized Pilot Study. Environ Res public Heal. 2020; doi: 10.3390/ijerph17176368.

3. Nisar A, Yin J, Nan Y, et al. Standardising Training of Nurses in an Evidence-Based Psychosocial Intervention for Perinatal Depression : Randomized Trial of Electronic vs . Face-to-Face Training in China. Int J Environ Res Public Heal. 2022; doi: 10.3390/ijerph19074094.

4. Pereira CA, Wen CL, Miguel EC, et al. A randomised controlled trial of a web ‑ based educational program in child mental health for schoolteachers. Eur Child Adolesc Psychiatry. 2015; doi: 10.1007/s00787-014-0642-8.

5. Maulik PK, Kallakuri S, Devarapalli S, Jha V, Patel A. Increasing use of mental health services in remote areas using mobile technology : a pre – post evaluation of the SMART Mental Health project in rural India. J Glob Health. 2017;7(1).

6. Maulik PK, Devarapalli S, Kallakuri S. The Systematic Medical Appraisal Referral and Treatment Mental Health Project : Quasi-Experimental Study to Evaluate a Technology-Enabled Mental Health Services Delivery Model Implemented in Rural India Corresponding Author : J Med Internet Res. 2020;22(e15553):1–11.

7. Doukani A, Sera F, Chibanda D. A community health volunteer delivered problem-solving therapy mobile application based on the Friendship Bench ‘ Inuka Coaching ’ in Kenya : A pilot cohort study. Glob Ment Heal. 2022;8(e9):1–11.

8. Dambi J, Norman C, Doukani A, Potgieter S, Turner J, Musesengwa R, et al. A Digital Mental Health Intervention (Inuka) for Common Mental Health Disorders in Zimbabwean Adults in Response to the COVID-19 Pandemic: Feasibility and Acceptability Pilot Study. JMIR Ment Heal. 2022;9(10): doi: https://doi.org/10.2196/37968.

9. Chibanda D, Weiss HA, Verhey R, et al. Effect of a Primary Care–Based Psychological Intervention on Symptoms of Common Mental Disorders in Zimbabwe A Randomized Clinical Trial. JAMA. 2016; doi: 10.1001/jama.2016.19102.

10. Ross R, Sawatphanit W, Suwansujarid T, et al. The Effect of Telephone Support on Depressive Symptoms Among HIV-Infected Pregnant Women in Thailand: An Embedded Mixed Methods Study. JANAC J Assoc Nurses AIDS Care. 2013; doi: 10.1016/j.jana.2012.08.005.

11. Ebrahem SM, Badawy SA, Hassan RA, et al.. Effect of Telehealth Nursing Intervention on Psychological Status and Coping Strategies Among Parents During COVID-19 Pandemic. Holist Nurs Pract. 2023; doi: 10.1097/HNP.0000000000000561.

12. Scazufca M, Clara M, Couto PDP, et al. Pilot study of a two-arm non-randomized controlled cluster trial of a psychosocial intervention to improve late life depression in socioeconomically deprived areas of São Paulo , Brazil ( PROACTIVE ): feasibility study of a psychosocial intervention for lntervention for late life depression in Sao Pãulo. BMC Public Health. 2019; doi: 10.1186/s12889-019-7495-5.

13. Garg A, Agrawal R, Velleman R, et al. Integrating assisted tele-psychiatry into primary healthcare in Goa, India: a feasibility study. Glob Ment Heal. 2022; doi: 10.1017/gmh.2021.47.

14. Liu Y, Hasimu M, Joa M, Tang J, Wang Y, He X, et al. The effect of a APP-Based Intervention for Depression Among Community-Dwelling Individuals With Spinal Cord Injury: A randomized Controlled Trial. Arch Phys Med Rehabil. 2023; doi: 10.1016/j.apmr.2022.10.005.

15. Öztoprak PU, Koç G, Erkaya S. Evaluation of the effect of a nurse navigation program developed for postpartum mothers on maternal health: A randomized controlled study. Public Health Nurs. 2023; doi: 10.1111/phn.13226.

16. Hong S, Lee S, Song K, et al. A nurse-led mHealth intervention to alleviate depressive symptoms in older adults living alone in the community: A quasi-experimental study. Int J Nurs Stud. 2023; doi: 10.1016/j.ijnurstu.2022.104431.

17. Noor Hanita Z, Khatijah LA, Kamaruzzaman S. A pilot study on development and feasibility of the ‘MyEducation: CABG application’ for patients undergoing coronary artery bypass graft (CABG) surgery. BMC Nurs. 2022; doi: 10.1186/s12912-022-00814-4.

18. Xu X, Chen S, Chen J, et al.Feasibility and Preliminary Efficacy of a Community-Based Addiction Rehabilitation Electronic System in Substance Use Disorder : Pilot Randomized Controlled Trial. JMIR mHealth uHealth. 2021; doi: 10.2196/21087.

19. Rodriguez M, Eisenlohr-moul TA, Weisman J, et al. The Use of Task Shifting to Improve Treatment Engagement in an Internet-Based Mindfulness Intervention Among Chinese University Students : Randomized Controlled Trial. JMIR Form Res. 2021; doi: 10.2196/25772.

20. Anttila M, Sittichai R, Katajisto J, et al. Impact of a Web Program to Support the Mental Wellbeing of High School Students : A Quasi Experimental Feasibility Study. Environ Res public Heal. 2019; doi: 10.3390/ijerph16142473.

21. Menezes P, Quayle J, Paulo S. Use of a Mobile Phone App to Treat Depression Comorbid With Hypertension or Diabetes : A Pilot Study in Brazil and Peru JMIR Ment Heal. 2019; doi: 10.2196/11698.

22. Zhou K, Li J, Li X. Effects of cyclic adjustment training delivered via a mobile device on psychological resilience , depression , and anxiety in Chinese post ‑ surgical breast cancer patients. Breast Cancer Res Treat. 2019; https://doi.org/10.1007/s10549-019-05368-9

23. Gonsalves PP, Hodgson ES, Bhat B, et al. App- based guided problem- solving intervention for adolescent mental health: a pilot cohort study in Indian schools. Evid Based Ment Heal. 2021; doi: 10.1136/ebmental-2020-300194.

24. Arjadi R, Nauta MH, Scholte WF, et al. Internet-based behavioural activation with lay counsellor support versus online minimal psychoeducation without support for treatment of depression : a randomised controlled trial in Indonesia. The Lancet Psychiatry. 2018; doi: 10.1016/S2215-0366(18)30223-2.

25. Araya R, Menezes PR, Claro HG, et al. Effect of a Digital Intervention on Depressive Symptoms in Patients With Comorbid Hypertension or Diabetes in Brazil and Peru Two Randomized Clinical Trials. JAMA. 2022; doi: 10.1001/jama.2021.4348.

26. Khan MN, Hamdani SU, Chiumento A, et al. Evaluating feasibility and acceptability of a group WHO trans-diagnostic intervention for women with common mental disorders in rural Pakistan: A cluster randomised controlled feasibility trial. Epidemiol Psychiatr Sci. 2019; doi: 10.1017/S2045796017000336.

27. Rahman A, Khan MN, Hamdani SU, Chiumento A, Akhtar P, Nazir H, et al. Effectiveness of a brief group psychological intervention for women in a post-conflict setting in Pakistan: a single-blind, cluster, randomised controlled trial. Lancet. 2019; doi: 10.1016/S0140-6736(18)32343-2.

28. Chen S, Conwell Y, Xue J, et al. Effectiveness of integrated care for older adults with depression and hypertension in rural China: A cluster randomized controlled trial. PLoS Med. 2022;doi: http://dx.doi.org/10.1371/journal.pmed.1004019

29. Barnett, M. L., Gonzalez, A., Miranda, J., et al. Mobilizing Community Health Workers to Address Mental Health Disparities for Underserved Populations: A Systematic Review. Adm Policy Ment Health. 2018;45(2):195–211. doi: 10.1007/s10488-017-0815-0.

30. Barnett ML, Puffer ES, Ng LC, Jaguga F. Effective training practices for non-specialist providers to promote high-quality mental health intervention delivery: A narrative review with four case studies from Kenya, Ethiopia, and the United States. Cambridge Prism Glob Ment Heal. 2023;10(e26):1–9. Doi: 10.1017/gmh.2023.19
