# Supplementary material for: The effectiveness of mental health interventions involving non-specialists and digital technology in low-and middle-income countries – a systematic review
Source: BMC Public Health. 2024 Jan 3;24:77. doi: 10.1186/s12889-023-17417-6 (PMC10763181; doi:10.1186/s12889-023-17417-6)
Supplement: Supplementary file 11 — Additional file 11. [file 12889_2023_17417_MOESM11_ESM.docx]

# ADDITIONAL FILE 11: RISK OF BIAS ASSESSMENTS

**Table S11. Risk of bias assessments**

| **Bias assessment of individual randomised controlled trials^1^** | | | | | | | | |
| --- | --- | --- | --- | --- | --- | --- | --- | --- |
| **First author (year)** | **Bias arising from the randomization process** | **Bias due to deviations from the intended intervention** | **Bias due to missing outcome data** | **Bias in measurement of the outcome** | **Bias in selection of the reported results** | **Overall rating** |  | |
| Ross, 2013 | High | Some concern | Low | High | Some concern | High |  |  |
| Nisar, 2020 | Low | Some concern | Low | Low | Some concern | Some concern |  |  |
| Rahman, 2019 | Low | Some concern | Low | Low | Low | Some concern |  |  |
| Araya, 2021 (Peru) | Low | Low | Low | Low | Low | Low |  |  |
| Arjadi, 2018 | Low | Low | Low | Low | Low | Low |  |  |
| Zhou, 2019 | Some concern | Some concern | Low | Low | Some concern | Some concern |  |  |
| Rodriguez, 2021 | Some concern | Some concern | Low | Some concern | Some concern | High |  |  |
| Liu, 2023 | Low | Some concern | Low | Low | Some concern | Some concern |  |  |
| Öztoprak, 2023 | Low | Some concern | Low | High | Low | High |  |  |
| **Bias assessment of cluster randomised controlled trials^2^** | | | | | | | | |
| **First author (year)** | **Bias arising from the randomization process** | **Bias arising from the timing of identification and recruitment of individual participants in relation to timing of randomization** | **Bias due to deviations from the intended intervention** | **Bias due to missing outcome data** | **Bias in measurement of the outcome** | **Bias in selection of the reported results** | **Overall rating** |  |
| Araya, 2021 (Brazil) | Low | Low | Some concern | Low | Low | Low | Some concern |  |
| Chibanda, 2016 | Some concern | Low | Low | Low | Low | Low | Some concern |  |
| Pereira, 2015 | Some concern | Low | Some concern | Low | High | Some concern | High |  |
| Rahman, 2019 | Low | Low | Low | Low | Low | Low | Low |  |
| Chen, 2022 | High | High | Some concern | Low | Low | High | High |  |
| **Bias assessment of non-randomized controlled trials^3^** | | | | | | | | |
| **First author (year)** | **Bias due to confounding** | **Bias due to selection of participants** | **Bias in classification of interventions** | **Bias due to deviations from intended intervention** | **Bias due to missing outcome data** | **Bias in measurement of the outcome** | **Bias in selection of the reported results** | **Overall rating** |
| Maulik, 2017 | Serious | Serious | Serious | No information | Low | Moderate | No information | Serious |
| Maulik, 2020 | Serious | Serious | Serious | No information | Low | Moderate | No information | Serious |
| Ebrahem, 2023 | Critical | Critical | Serious | No information | Critical | Serious | No information | Critical |
| Hong, 2023 | Moderate | Low | Low | Low | Moderate | Moderate | Moderate | Moderate |
| Notes:  ^1^ Bias assessment of individual randomised controlled trials was conducted using the Cochrane Risk of Bias 2.0 (RoB 2.0) tool. ^2^Bias assessment of cluster randomised controlled trails was conducted using the Cochrane RoB 2 with Additional considerations for cluster-randomized trials (RoB 2.0 CRCT) tool. ^3^Bias assessment of non-randomised controlled trials was conducted using the Risk Of Bias In Non-randomized Studies of Interventions (ROBINS-I) tool. T | | | | | | | | |
