# Supplementary material for: The effectiveness of mental health interventions involving non-specialists and digital technology in low-and middle-income countries – a systematic review
Source: BMC Public Health. 2024 Jan 3;24:77. doi: 10.1186/s12889-023-17417-6 (PMC10763181; doi:10.1186/s12889-023-17417-6)
Supplement: Supplementary file 12 — Additional file 12. [file 12889_2023_17417_MOESM12_ESM.docx]

# **ADDITIONAL FILE 12: GRADE ASSESSMENTS**

**Table S12 Certainty of evidence assessment using the GRADE tool^1^**

| **Domain: Digital training of non-specialists** | | |
| --- | --- | --- |
| **Grade domain** | **Judgement** | **Concerns about certainty domains** |
| Methodological limitations of the study | Three studies were RCTs, while one study was a randomized pilot study, which can be judged as high risk of bias, given the unpowered results. Among the three RCTs, two were judged to be at high risk of bias and one revealed some concerns (Additional file 11).  Therefore, these trials can be judged as having serious methodological limitations. | Serious |
| Indirectness | The population (mostly female) and setting (LMICs) were similar in all studies (Table 1). Three studies investigated a digital training for non-specialist enabling them to provide treatment. In contrast, one study examined whether mental health education may increase more awareness and decrease stigma in school settings, relating to a mental health promotion approach (Additional file 6). Moreover, the outcomes were assessed using different measurements (Table 3, Additional file 7). Given that the majority of studies investigated a similar intervention, while only one differed and because of the variation in outcome measures, the trials can be judged to indicate borderline indirectness. | Serious, borderline |
| Imprecision | In total, around 389 participants were included in the four trials. Moreover, three among the four studies conducted an a-priori sample size calculation indicating that their results provide >80% power (1–3). Nevertheless, because a threshold of n=400 or less is concerning for imprecision (4),  the given evidence can be judged as indicating borderline serious imprecision. | Serious, borderline |
| Inconsistency | Most studies reported small effectiveness of the intervention as compared to the control, except from two which revealed medium effectiveness. Furthermore, most studies favored the intervention group over the control (Table 3). Only one study reported higher competence level in the control group but evaluated these results as non-inferior to the control group based on the non-inferiority margin (1). Hence, the given evidence can be judged as not being seriously inconsistent. | Not serious |
| Publication bias | All outcomes were presented, despite revealing small effectiveness and non-significant results. However, 3 studies were conducted in Asian settings and 1 in South America (Table 1). No studies from African country settings were included, which may indicate a publication bias. Hence, we judge the overall evidence to have borderline serious publication bias. | Serious, borderline |
| **Domain: Digital support for non-specialist delivered interventions** | | |
| **Grade domain** | **Judgement** | **Concerns about certainty domains** |
| Methodological limitations of the study | Among the n=11 included studies, four were RCTs, three were NRCTs, and four were pilot/feasibility trials. The RCTs were judged to be at high risk of bias, or with some concerns for bias. The NRCTs were judged to be at serious or critical risk of bias (Additional file 11). We consider that the results of the pilot and feasibility studies were at high risk of bias given the small sample sizes. Therefore, the included trials can be judged as having serious methodological limitations. | Serious |
| Indirectness | Most of the included studies investigated people with common mental disorders (CMD) or just depression, anxiety, substance or alcohol use disorders with or without comorbidities. Among those studies, the assessment tools varied from validated structured assessment tools to clinical evaluations (table 1). Additionally, different interventions were offered in which the technology had different clinical functions (i.e., sometimes as a decision support tool for the non-specialist and sometimes for organization purposes or for remote asynchronous or synchronous communication). Additionally, different treatment and care interventions were delivered with different lengths (additional file 6 and 10). Given the heterogeneity in the study population, interventions and outcomes the evidence can be judged as being seriously indirect. | Serious |
| Imprecision | The total number of participants at baseline of all included trials was n=2469. While five studies revealed significant effects based with 80% power (5–9), two studies did not report on the significance of the outcome of intertest (10,11) and three studies revealed significant outcome which were underpowered (12–14) Because half of the results revealed unpowered effects, the provided evidence can be judged as implying borderline serious imprecision. | Serious, borderline |
| Inconsistency | Most outcomes across the studies favored the intervention group with mostly large or medium effect sizes (Table 3). Hence, the available evidence can be judged as revealing no serious inconsistency. | Not serious |
| Publication bias | All outcomes were presented, despite revealing small effectiveness. Furthermore, studies were conducted in Asian, South American and African country settings (Table 1). Therefore, although a publication bias cannot be ruled out, it seems unlikely. | Not serious |
| **Domain: Digitally delivered treatment with non-specialist supportc** | | |
| **Grade domain** | **Judgement** | **Concerns about certainty domains** |
| Methodological limitations of the study | Among the included studies, five were pilot or feasibility studies, four were RCTs and one was an NRCT (Table 2). The pilot and feasibility studies can be considered to generate result with high risk of bias given the small sample size. Among the RCTs one study was judged to be at high risk of bias, two interventions revealed some concern, and two interventions were at low risk of bias (Additional file 11). Given that the majority of intervention were at high risk of bias or had some concern, serious methodological limitations in the given evidence can be concluded. | Serious |
| Indirectness | The health status of the population was very heterogenous in terms of diagnosed disorder and underlying (severity) of mental health complaints. Moreover, the measurement tools differed across the studies (Table 1). Hence, the given evidence can be evaluated to reveal serious indirectness. | Serious |
| Imprecision | The total number of participants in these trials was n=2314. However, because half of the studies examined small samples and therefore provided unpowered results (Table 1), the provided evidence can be judged as implying borderline serious imprecision. | Serious, borderline |
| Inconsistency | Most outcomes across the studies favored the intervention group with mostly small effect sizes (Table 3). Hence, the available evidence can be judged as revealing no serious inconsistency. | Not serious |
| Publication bias | All outcomes were presented, despite revealing small effectiveness. However, none of the studies were conducted in African country settings (Table 1). Hence, we judge the overall evidence to have borderline serious publication bias. | Serious, borderline |
| **Domain: Digital supervision of non-specialists** | | |
| Methodological limitations of the study | Among the three studies included in this domain, one was a feasibility study, which can be judged as high risk of bias given the small sample size and unpowered results. The other included studies were two cluster RCTs which were judged to be at low and high risk of bias (Additional file 11). Hence, because two out of three studies had a high risk of bias, overall evidence was judged to reveal a serious methodological limitation of the study. | Serious |
| Indirectness | All studies focused on outcomes relating to the severity of mental illness of the participants. These outcomes indirectly indicate that interventions, in which the digital technology is used to supervise the non-specialists are effective for the non-specialists, by showing that those people who receive treatment from the non-specialist show improvements in mental health status. However, it is not clear how effective the digital supervision component is in comparison to for example face-to-face supervision or no supervision. Thus, we judge the available evidence to be seriously indirect. | Serious |
| Imprecision | Evidence in this domain is based on three studies with n=3096 participants in total. Moreover, two studies provided revealed significant effects with 80% power. Hence, we judged that this evidence at had is not seriously imprecise. | Not serious |
| Inconsistency | All studies show improvements in the psychological well-being of the participants receiving the treatment intervention (Table 3). Hence, the evidence at hand can be judged as not being seriously inconsistent. | Not serious |
| Publication bias | Two studies were conducted in Pakistan and one in China. The two studies conducted in Pakistan studies originated from the same research group investigating the same intervention in the same population setting only at a different time-point with slightly different inclusion criteria for recruitment (Table 1). Hence, we judge the available evidence to have a serious risk of publication bias. | Serious |
| ^1^The certainty assessment is based on the guidelines proposed by Grading of Recommendations, Assessment, Development and Evaluation (GRADE) Working Group (15) and recommendation for using the GRADE assessment tool in narrative synthesis (16). | | |

**Table S12 Summary of findings table^1^ for domain: Digital training of non-specialists**

| **Outcome** | **Effect** | **Number of participants (studies)** | **Certainty in the evidence*** |
| --- | --- | --- | --- |
| Competence and knowledge of non-specialists | Most studies showed an increase in competence and knowledge with small effectiveness. | N=398 (4) | BETWEEN LOW AND MODERATE^2,3,4^  ⊕⊕ |
| The outcome of interest is competence and knowledge level of non-specialist to provide a mental health intervention, for which a narrative synthesis is provided.  * high certainty ⊕⊕⊕⊕, moderate certainty ⊕⊕⊕O, low certainty ⊕⊕OO and very low certainty ⊕OOO.  ^1^This table was based on the recommendations of Murad et al. and the GRADE approach (15,16). ^2^Serious risk of bias.^3^ Borderline serious indirectness resulted from the heterogeneity of interventions and borderline imprecision resulted from generally small number of participants across studies and. These concepts were grouped together, given that they were judged to be borderline serious. ^4^ Borderline serious publication bias, because the study reported on outcomes favoring intervention and control, but concurrently no studies conducted in African country settings were included in this domain. | | | |

**Table S13. Summary of findings table^1^ for domain: Digital support for non-specialists-delivered interventions**

| **Outcome** | **Effect** | **Number of participants (studies)** | **Certainty in the evidence*** |
| --- | --- | --- | --- |
| Treatment behavior, severity of mental health problems and psychosocial functioning | Most studies favoured the intervention compared to different control conditions with regards to increased mental healthcare use, decreased severity of mental health problems and increased psychosocial functioning with  Mostly medium to large effect sizes. | N=2759 (6) | LOW^2,3, 4^  ⊕ O O |
| The outcome of interest is related to mental health treatment and includes mental health treatment behavior and severity of mental health complaints for which a narrative synthesis is provided.  * high certainty ⊕⊕⊕⊕, moderate certainty ⊕⊕⊕O, low certainty ⊕⊕OO and very low certainty ⊕OOO.  ^1^This table was based on the recommendations of Murad et al. and the GRADE approach (15,16). ^2^Serious risk of bias ^3^ Serious indirectness resulted from the high heterogeneity of the PICO characteristics across the studies. ^4^ Borderline serious imprecision resulted from the fact that only half of the studies had optimal information size for the outcome of interest in this review. | | | |

**Table S14** **Summary of findings table^1^ for domain: Digitally delivered treatment with non-specialist support**

| **Outcome** | **Effect** | **Number of participants (studies)** | **Certainty in the evidence*** |
| --- | --- | --- | --- |
| Severity of mental health problems and psychosocial functioning | Most studies favoured the intervention in contrast to different control conditions with regards to decreased severity of mental health problems and increased psychosocial functioning. | N=2314 (10) | LOW^2,3,4,5^  ⊕ |
| The outcome of interest is related to mental health treatment and includes mental health treatment behavior and severity of mental health complaints for which a narrative synthesis is provided.  * high certainty ⊕⊕⊕⊕, moderate certainty ⊕⊕⊕O, low certainty ⊕⊕OO and very low certainty ⊕OOO.  ^1^This table was based on the recommendations of Murad et al. and the GRADE approach (15,16). ^2^Serious risk of bias^3^ Serious indirectness resulted from the high heterogeneity of the PICO characteristics across the studies.^4^ Borderline serious imprecision resulted from the fact that only half of the studies had optimal information size for the outcome of interest in this review. ^5^ Borderline serious publication bias, because the study reported on outcomes favoring intervention and control, but concurrently no studies conducted in African country settings were included in this domain. These concepts were grouped together, given that they were judged to be borderline serious. | | | |

**Table S15. Summary of findings table^1^ for domain: Digital supervision of non-specialist**

| Outcome | Effect | Number of participants (studies) | Certainty in the evidence* |
| --- | --- | --- | --- |
| Mental health well-being and symptom severity | All studies showed a decrease in symptom severity with medium effect size. | n=731 (2) | BETWEEN LOW AND VERY LOW^2,3,4^  ⊕ |
| The outcome of interest is related to mental health treatment and includes mental health treatment behavior and severity of mental health complaints for which a narrative synthesis is provided.  * high certainty ⊕⊕⊕⊕, moderate certainty ⊕⊕⊕O, low certainty ⊕⊕OO and very low certainty ⊕OOO.  ^1^This table was based on the recommendations of Murad et al. and the GRADE approach (15,16). ^2^ Serious risk because 2/3 have a high risk for bias,  ^3^ Serious indirectness because the results do not directly indicate the effectiveness of digital supervision compared to no supervision or face-to-face supervision.^4^ Serious publication bias, because two studies (out of three) were derived from the same research group and investigated the same intervention in slightly different population settings. | | | |

**References:**

1. Nisar A, Yin J, Nan Y, Luo H, Han D, Yang L, et al. Standardising Training of Nurses in an Evidence-Based Psychosocial Intervention for Perinatal Depression : Randomized Trial of Electronic vs . Face-to-Face Training in China. Int J Environ Res Public Heal. 2022;19(4094):1–13.

2. Rahman A, Akhtar P, Hamdani SU, Atif N, Nazir H, Uddin I, et al. Using technology to scale-up training and supervision of community health workers in the psychosocial management of perinatal depression: a non-inferiority, randomized controlled trial. Glob Ment Heal. 2019;6:1–5.

3. Pereira CA, Wen CL, Miguel EC, Polanczyk G V. A randomised controlled trial of a web ‑ based educational program in child mental health for schoolteachers. Eur Child Adolesc Psychiatry [Online]. 2015;24:931–40. doi: http://dx.doi.org/10.1007/s00787-014-0642-8

4. Guyatt GH, Oxman AD, Kunz R, Brozek J, Alonso-Coello P, Rind D, et al. GRADE guidelines 6. Rating the quality of evidence - Imprecision. J Clin Epidemiol. 2011;64(12):1283–93.

5. Ebrahem SM, Badawy SA, Hassan RA, Radwan HA, Shokr EA, Hussein AA. Effect of Telehealth Nursing Intervention on Psychological Status and Coping Strategies Among Parents During COVID-19 Pandemic. Holist Nurs Pract. 2023;37(1):34–44.

6. Liu Y, Hasimu M, Joa M, Tang J, Wang Y, He X, et al. The effect of a APP-Based Intervention for Depression Among Community-Dwelling Individuals With Spinal Cord Injury: A randomized Controlled Trial. Arch Phys Med Rehabil. 2023;104:195–202.

7. Öztoprak PU, Koç G, Erkaya S. Evaluation of the effect of a nurse navigation program developed for postpartum mothers on maternal health: A randomized controlled study. Public Health Nurs. 2023;40(5):672–84.

8. Chibanda D, Weiss HA, Verhey R, Simms V, Munjoma R, Rusakaniko S, et al. Effect of a Primary Care–Based Psychological Intervention on Symptoms of Common Mental Disorders in Zimbabwe A Randomized Clinical Trial. JAMA. 2016;316(24):2618–26.

9. Maulik PK, Devarapalli S, Kallakuri S. The Systematic Medical Appraisal Referral and Treatment Mental Health Project : Quasi-Experimental Study to Evaluate a Technology-Enabled Mental Health Services Delivery Model Implemented in Rural India Corresponding Author : J Med Internet Res. 2020;22(e15553):1–11.

10. Maulik PK, Kallakuri S, Devarapalli S, Jha V, Patel A. Increasing use of mental health services in remote areas using mobile technology : a pre – post evaluation of the SMART Mental Health project in rural India. J Glob Health. 2017;7(1).

11. Ross R, Sawatphanit W, Suwansujarid T, Stidham AW, Drew BL, Creswell JW. The Effect of Telephone Support on Depressive Symptoms Among HIV-Infected Pregnant Women in Thailand: An Embedded Mixed Methods Study. JANAC J Assoc Nurses AIDS Care [Internet]. 2013 Sep;24(5):e13-24. Available from: https://search.ebscohost.com/login.aspx?direct=true&db=cin20&AN=104213263&site=ehost-live

12. Dambi J, Norman C, Doukani A, Potgieter S, Turner J, Musesengwa R, et al. A Digital Mental Health Intervention (Inuka) for Common Mental Health Disorders in Zimbabwean Adults in Response to the COVID-19 Pandemic: Feasibility and Acceptability Pilot Study. JMIR Ment Heal. 2022;9(10):35960595.

13. Garg A, Agrawal R, Velleman R, Rane A, Costa S, Gupta D, et al. Integrating assisted tele-psychiatry into primary healthcare in Goa, India: a feasibility study. Glob Ment Heal. 2022;9:26–36.

14. Doukani A, Sera F, Chibanda D. A community health volunteer delivered problem-solving therapy mobile application based on the Friendship Bench ‘ Inuka Coaching ’ in Kenya : A pilot cohort study. Glob Ment Heal. 2022;8(e9):1–11.

15. Schünemann HJ, Higgins JPT, Vist GE, Glasziou P, Akl EA, Skoetz N GG. Chapter 14: Completing ‘Summary of findings’ tables and grading the certainty of the evidence. In: Higgins JPT, Thomas J, Chandler J, Cumpston M, Li T, Page MJ WV, editor. Cochrane Handbook for Systematic Reviews of Interventions version 63 (updated February 2022) [Internet]. 2022. Available from: www.training.cochrane.org/handbook.

16. Murad MH, Mustafa RA, Schünemann HJ, Sultan S, Santesso N. Rating the certainty in evidence in the absence of a single estimate of effect. Evid Based Med. 2017;22(3):85–7.
