# Supplementary material for: The effectiveness of mental health interventions involving non-specialists and digital technology in low-and middle-income countries – a systematic review
Source: BMC Public Health. 2024 Jan 3;24:77. doi: 10.1186/s12889-023-17417-6 (PMC10763181; doi:10.1186/s12889-023-17417-6)
Supplement: Supplementary file 13 — Additional file 13. [file 12889_2023_17417_MOESM13_ESM.docx]

# **ADDITIONAL FILE 13: SENSITIVITY ANALYSIS OF DIFFERENT EFFECT MEASURES**

Table S13 shows the results of the sensitivity analysis comparing Cohen’s d with the statistical corrections Glass delta and Hedges g. The results show that the interpretation of the effect size did not change when using the different statistical corrections in most cases.

**Table S13. Sensitivity analysis comparing the interpretation of different effect sizes**

| **Author name and study reference** | **Outcome** | **Effect size considering the assumptions** | **Cohens d’** |
| --- | --- | --- | --- |
| Rahman (1) | Competence at 3 months post-baseline | ∆^1^= 0.13; small | d= 0.16; small |
| Muke (DGT+) (2) | Competence at 1 month post baseline | g^2^=0.66; medium | d=0.7; medium |
| Muke (DGT) (2) | Competence at 1 month post baseline | g^2^= 0.3; small | d= 0.32; small |
| Xu (3) | Longest period of absence at 6 months post-baseline | ∆^1^=0.52; medium | d=0.62; medium |
| Chen (4) | Depressive symptoms at 3 months post baseline | ∆^1^=-0.32; medium | d=-0.43; medium |
|  | Depressive symptoms at 6 months post baseline | ∆^1^=-0.85; large | d=-0.82; large |
|  | Depressive symptoms at 12 months post baseline | **∆^1^=-1.3; large** | **d= -0.4, medium** |
| Öztoprak (5) | Quality of life at 3 months post-partum | ∆^1^=2.75; large | d= 3.39, large |
|  | Anxiety at 6 weeks post-partum | ∆^1^=-2.42; large | d= -3.17, large |
|  | Anxiety at 3 months post-partum | ∆^1^=-2.45; large | d= -3.28, large |
|  | Depression at 10 days post-partum | ∆^1^=-1.54; large | d= -1.14, large |
|  | Depression at 3 months post-partum | ∆^1^=-1.54; large | d= -1.86, large |
| Notes: ^1^∆= based on Glass delta; ^2^g= based on Hedge’s g | | | |

References:

1. Rahman A, Akhtar P, Hamdani SU, et al. Using technology to scale-up training and supervision of community health workers in the psychosocial management of perinatal depression: a non-inferiority, randomized controlled trial. Glob Ment Heal. 2019; doi: 10.1017/gmh.2019.7

2. Muke SS, Tugnawat D, Joshi U, et al. Digital Training for Non-Specialist Health Workers to Deliver a Brief Psychological Treatment for Depression in Primary Care in India:Findings from a Randomized Pilot Study. Environ Res public Heal. 2020; doi: 10.3390/ijerph17176368.

3. Xu X, Chen S, Chen J, et al.Feasibility and Preliminary Efficacy of a Community-Based Addiction Rehabilitation Electronic System in Substance Use Disorder : Pilot Randomized Controlled Trial. JMIR mHealth uHealth. 2021; doi: 10.2196/21087.

4. Chen S, Conwell Y, Xue J, et al. Effectiveness of integrated care for older adults with depression and hypertension in rural China: A cluster randomized controlled trial. PLoS Med. 2022;doi: http://dx.doi.org/10.1371/journal.pmed.1004019

5. Öztoprak PU, Koç G, Erkaya S. Evaluation of the effect of a nurse navigation program developed for postpartum mothers on maternal health: A randomized controlled study. Public Health Nurs. 2023; doi: 10.1111/phn.13226.
