# Supplementary material for: The effectiveness of mental health interventions involving non-specialists and digital technology in low-and middle-income countries – a systematic review
Source: BMC Public Health. 2024 Jan 3;24:77. doi: 10.1186/s12889-023-17417-6 (PMC10763181; doi:10.1186/s12889-023-17417-6)
Supplement: Supplementary file 14 — Additional file 14. [file 12889_2023_17417_MOESM14_ESM.docx]

# **ADDITIONAL FILE 14: SENSITIVITY ANALYSIS OF DIFFERENT CORRELATION COEFFICIENTS**

Table S14 shows the results of a sensitivity analysis comparing the effect size Cohen’s for different outcomes when using different regression coefficients. The results show that in 4 out of 22 outcomes, the interpretation varied from medium to large, when using a different regression coefficient. The remaining outcomes had the same interpretation of the effect size when using the three different correlation coefficients (r).

**Table S14. Sensitivity analysis comparing effect sizes using different r**

| **Author, study reference** | **outcome** | **Cohens d with r= 0.5** | **Cohens d with r= 0.2** | **Cohens d with r= 0.8** |
| --- | --- | --- | --- | --- |
| Muke (DGT intervention), (1) | Competence | 0.32, medium | 1.25, large | 0.63, medium |
| Muke, (DGT+ intervention) (1) | Competence | 0.7, medium | 1.26, large | 0.63, medium |
| Doukani (2) | Severity of CMD | -1, large | -1.06, large | -0.82, large |
|  | Severity of depression | -0.84, large | -0.84, large | -0.84, large |
|  | Severity of anxiety | -0.83, large | -1,26, large | -0.77, medium |
| Dambi (Inuka intervention) (4) | Severity of CMD | -1.09, large | -1.1, large | -1.05, large |
|  | Severity of depression | -0.7, large | -0.7, large | -0.7, large |
|  | Severity of anxiety | -0.62, medium | -0.64, medium | -0.6, medium |
|  | Disability and functioning | -0.6, medium | -0.63, medium | -0.5, medium |
|  | Quality of life | 0.39, medium | 0.39, medium | 0.39, medium |
| Dambi (Whatsapp active control) (4) | Severity of CMD | -0.88, large | -1.31, large | -0.86, large |
|  | Severity of depression | -1.35, large | -1.36, large | -1.32, large |
|  | Severity of anxiety | -1.11, large | -1.11, large | -1.11, large |
|  | Disability and functioning | -0.91, large | -1.01, large | -0.7, medium |
|  | Quality of life | 0.06, small | 0.08, small | 0.04, small |
| Anttila, intervention (3) | Severity of depressive symptoms | -0.07, small | -0.07, small | -0.07, small |
|  | Stress level | 0.14, small | 0.14, small | 0.14, small |
| Anttila, (active control) (3) | Severity of depressive symptoms | -0.27, small | -0.28, small | -0.27, small |
|  | Stress level | -0.09, small | -0.09, small | -0.09, small |
| Garg, 2022 (5) | Severity of CMD | -1.13, large | -1.14, large | -1.12, large |
|  | Alcohol use disorder | -3.22, large | -3.39, large | -2.74, large |
|  | Disability and functioning | -0.69, medium | -0.7, medium | -0.63, medium |

**References**

1. Muke SS, Tugnawat D, Joshi U, et al. Digital Training for Non-Specialist Health Workers to Deliver a Brief Psychological Treatment for Depression in Primary Care in India:Findings from a Randomized Pilot Study. Environ Res public Heal. 2020; doi: 10.3390/ijerph17176368.

2. Doukani A, Sera F, Chibanda D. A community health volunteer delivered problem-solving therapy mobile application based on the Friendship Bench ‘ Inuka Coaching ’ in Kenya : A pilot cohort study. Glob Ment Heal. 2022; doi: 10.1017/gmh.2021.3.

3. Anttila M, Sittichai R, Katajisto J, et al. Impact of a Web Program to Support the Mental Wellbeing of High School Students : A Quasi Experimental Feasibility Study. Environ Res public Heal. 2019; doi: 10.3390/ijerph16142473.

4. Dambi J, Norman C, Doukani A, Potgieter S, Turner J, Musesengwa R, et al. A Digital Mental Health Intervention (Inuka) for Common Mental Health Disorders in Zimbabwean Adults in Response to the COVID-19 Pandemic: Feasibility and Acceptability Pilot Study. JMIR Ment Heal. 2022;9(10): doi: https://doi.org/10.2196/37968.

5. Garg A, Agrawal R, Velleman R, et al. Integrating assisted tele-psychiatry into primary healthcare in Goa, India: a feasibility study. Glob Ment Heal. 2022; doi: 10.1017/gmh.2021.47.
