# Supplementary material for: The effectiveness of mental health interventions involving non-specialists and digital technology in low-and middle-income countries – a systematic review
Source: BMC Public Health. 2024 Jan 3;24:77. doi: 10.1186/s12889-023-17417-6 (PMC10763181; doi:10.1186/s12889-023-17417-6)
Supplement: Supplementary file 15 — Additional file 15. [file 12889_2023_17417_MOESM15_ESM.docx]

# **ADDITIONAL FILE 15: REPORTED SOURCES OF STUDY FUNDING**

**Table S15. Reported sources of funding for each included study**

| **Domain** | **Author, study reference** | **Sources of study funding reported** |
| --- | --- | --- |
| ***Digital training of non-specialists*** | Rahman (1) | Grand Challenges Canada (#0596-04) |
|  | Muke (2) | Nationale Institute of Mental Health, USA (U19MH113211) |
|  |  |  |
|  | Nisar (3) | School of Nursing at Xi’an Jiaotong University and (partly funded by) University of Liverpool Industry Fund Award (JXR13462) |
|  | Pereira (4) | Master’s scholarship Fundação de Amparo à Pesquisa do Estado  de São Paulo (Grant no. 2010/14223-3) and research grant by the National Council for Scientific and Technological  Development |
|  |  |  |
| ***Digital support for non-specialists*** | Maulik (5) | WT/DBT Fellowship (IA/I/13/1/500879) and Grand Challenges Canada (0524-01-10) |
|  | Maulik (6) | n.m |
|  | Doukani (7) | Grand Challenges Canada (0793-05) |
|  | Dambi (8) | n.m |
|  | Chibanda (9) |  |
|  | Ross (10) | Kent State University Research Council, Summer Research and Creativity Activity Appointment Award, Nursing Cluster Award |
|  | Ebrahem (11) | n.m |
|  |  | Grand Challenges Canada (KCU-0087-042) |
|  | Scazufca (12) | Medical Research Council United Kingdon (MRC-UK, ML/l016702/1), Fundação de Amparo à Pesquisa do Estado de São Paulo (FAPESP/Brazil, 2013/50953–4), and Research productivity grant from CNPq Brazil |
|  | Öztoprak (13) | n.m |
|  | Garg (14) | Medical Research Council (MRC), UK, Grant No. MR/N021886/1. |
|  | Liu (15) | Natural Science Foundation of Guangdong Province, China; grant no: 2021A1515011800 and National Natural Science Foundation China; grant 71603293 |
| ***Digitally delivered treatment with non-specialist support*** | Hong (16) | National Research Foundation of Korea (NRF) funded  by the Ministry of Education (No. 2016R1D1A1B03932013 and  2020R1A6A1A03041989) |
|  | Hanita (17) | University of Malaya Research Grant (BK016–2014), Kuala Lumpur Malaysia |
|  | Xu (18) | Clinical Research Center, Shanghai Jiao Tong University School of  Medicine (DLY201818), Municipal Human Resources Development Program for Outstanding Young Talents in Medical and Health Sciences in Shanghai (2017YQ013), cience and Technology Innovation Plan in Shanghai (18411961200), Shanghai Key Laboratory of Psychotic Disorders (13DZ2260500), National Key R&D Program of China (2017YFC1310400), National Nature Science Foundation (81771436), Shanghai Clinical Research Center for Mental Health (19MC1911100) |
|  | Rodriguez (19) | Student Research Award by the Center for International Studies at Duke University |
|  | Anttila (20) | The Finnish Funding Agency for Technology and Innovation (1547/31/2012, 40245/12) |
|  | Menezes (21) | National Institute of Mental Health (1U19MH098780) |
|  | Zhou (22) | China Postdoctoral Science Foundation (Grant No. 2018M643678) and National Natural Science Foundation of China (Grant No. 81502700) |
|  | Gonsalves (23) | Wellcome Trust, UK (grant number 106919/Z/15/Z) |
|  | Arjadi (24) | Indonesia Endowment Fund for Education (Lembaga Pengelola Dana Pendidikan), Ministry of Finance Republic of Indonesia (790/LPDP/2013), University of Groningen |
|  | Araya (25) | National Institute of Mental Health (U19MH098780) |
| ***Digital supervision of non-specialists*** | Khan (26) | Office of Foreign Disaster Assistance |
|  | Rahman (27) | Office for Foreign Disaster Assistance |
|  | Chen (28) | National Institute of Mental Health  of the U.S. National  Institutes of Health under Award Number  R01MH100298 |

**References:**

1. Rahman A, Akhtar P, Hamdani SU, et al. Using technology to scale-up training and supervision of community health workers in the psychosocial management of perinatal depression: a non-inferiority, randomized controlled trial. Glob Ment Heal. 2019; doi: 10.1017/gmh.2019.7

2. Muke SS, Tugnawat D, Joshi U, et al. Digital Training for Non-Specialist Health Workers to Deliver a Brief Psychological Treatment for Depression in Primary Care in India:Findings from a Randomized Pilot Study. Environ Res public Heal. 2020; doi: 10.3390/ijerph17176368.

3. Nisar A, Yin J, Nan Y, et al. Standardising Training of Nurses in an Evidence-Based Psychosocial Intervention for Perinatal Depression : Randomized Trial of Electronic vs . Face-to-Face Training in China. Int J Environ Res Public Heal. 2022; doi: 10.3390/ijerph19074094.

4. Pereira CA, Wen CL, Miguel EC, et al. A randomised controlled trial of a web ‑ based educational program in child mental health for schoolteachers. Eur Child Adolesc Psychiatry. 2015; doi: 10.1007/s00787-014-0642-8.

5. Maulik PK, Kallakuri S, Devarapalli S, Jha V, Patel A. Increasing use of mental health services in remote areas using mobile technology : a pre – post evaluation of the SMART Mental Health project in rural India. J Glob Health. 2017;7(1).

6. Maulik PK, Devarapalli S, Kallakuri S. The Systematic Medical Appraisal Referral and Treatment Mental Health Project : Quasi-Experimental Study to Evaluate a Technology-Enabled Mental Health Services Delivery Model Implemented in Rural India Corresponding Author : J Med Internet Res. 2020;22(e15553):1–11.

7. Doukani A, Sera F, Chibanda D. A community health volunteer delivered problem-solving therapy mobile application based on the Friendship Bench ‘ Inuka Coaching ’ in Kenya : A pilot cohort study. Glob Ment Heal. 2022;8(e9):1–11.

8. Dambi J, Norman C, Doukani A, et al. A Digital Mental Health Intervention (Inuka) for Common Mental Health Disorders in Zimbabwean Adults in Response to the COVID-19 Pandemic: Feasibility and Acceptability Pilot Study. JMIR Ment Heal. 2022;9(10): doi: https://doi.org/10.2196/37968.

9. Chibanda D, Weiss HA, Verhey R, et al. Effect of a Primary Care–Based Psychological Intervention on Symptoms of Common Mental Disorders in Zimbabwe A Randomized Clinical Trial. JAMA. 2016; doi: 10.1001/jama.2016.19102.

10. Ross R, Sawatphanit W, Suwansujarid T, et al. The Effect of Telephone Support on Depressive Symptoms Among HIV-Infected Pregnant Women in Thailand: An Embedded Mixed Methods Study. JANAC J Assoc Nurses AIDS Care. 2013; doi: 10.1016/j.jana.2012.08.005.

11. Ebrahem SM, Badawy SA, Hassan RA, et al.. Effect of Telehealth Nursing Intervention on Psychological Status and Coping Strategies Among Parents During COVID-19 Pandemic. Holist Nurs Pract. 2023; doi: 10.1097/HNP.0000000000000561.

12. Scazufca M, Clara M, Couto PDP, et al. Pilot study of a two-arm non-randomized controlled cluster trial of a psychosocial intervention to improve late life depression in socioeconomically deprived areas of São Paulo , Brazil ( PROACTIVE ): feasibility study of a psychosocial intervention for lntervention for late life depression in Sao Pãulo. BMC Public Health. 2019; doi: 10.1186/s12889-019-7495-5.

13. Garg A, Agrawal R, Velleman R, et al. Integrating assisted tele-psychiatry into primary healthcare in Goa, India: a feasibility study. Glob Ment Heal. 2022; doi: 10.1017/gmh.2021.47.

14. Liu Y, Hasimu M, Joa M, Tang J, Wang Y, He X, et al. The effect of a APP-Based Intervention for Depression Among Community-Dwelling Individuals With Spinal Cord Injury: A randomized Controlled Trial. Arch Phys Med Rehabil. 2023; doi: 10.1016/j.apmr.2022.10.005.

15. Öztoprak PU, Koç G, Erkaya S. Evaluation of the effect of a nurse navigation program developed for postpartum mothers on maternal health: A randomized controlled study. Public Health Nurs. 2023; doi: 10.1111/phn.13226.

16. Hong S, Lee S, Song K, et al. A nurse-led mHealth intervention to alleviate depressive symptoms in older adults living alone in the community: A quasi-experimental study. Int J Nurs Stud. 2023; doi: 10.1016/j.ijnurstu.2022.104431.

17. Noor Hanita Z, Khatijah LA, Kamaruzzaman S. A pilot study on development and feasibility of the ‘MyEducation: CABG application’ for patients undergoing coronary artery bypass graft (CABG) surgery. BMC Nurs. 2022; doi: 10.1186/s12912-022-00814-4.

18. Xu X, Chen S, Chen J, et al.Feasibility and Preliminary Efficacy of a Community-Based Addiction Rehabilitation Electronic System in Substance Use Disorder : Pilot Randomized Controlled Trial. JMIR mHealth uHealth. 2021; doi: 10.2196/21087.

19. Rodriguez M, Eisenlohr-moul TA, Weisman J, et al. The Use of Task Shifting to Improve Treatment Engagement in an Internet-Based Mindfulness Intervention Among Chinese University Students : Randomized Controlled Trial. JMIR Form Res. 2021; doi: 10.2196/25772.

20. Anttila M, Sittichai R, Katajisto J, et al. Impact of a Web Program to Support the Mental Wellbeing of High School Students : A Quasi Experimental Feasibility Study. Environ Res public Heal. 2019; doi: 10.3390/ijerph16142473.

21. Menezes P, Quayle J, Paulo S. Use of a Mobile Phone App to Treat Depression Comorbid With Hypertension or Diabetes : A Pilot Study in Brazil and Peru JMIR Ment Heal. 2019; doi: 10.2196/11698.

22. Zhou K, Li J, Li X. Effects of cyclic adjustment training delivered via a mobile device on psychological resilience , depression , and anxiety in Chinese post ‑ surgical breast cancer patients. Breast Cancer Res Treat. 2019; https://doi.org/10.1007/s10549-019-05368-9

23. Gonsalves PP, Hodgson ES, Bhat B, et al. App- based guided problem- solving intervention for adolescent mental health: a pilot cohort study in Indian schools. Evid Based Ment Heal. 2021; doi: 10.1136/ebmental-2020-300194.

24. Arjadi R, Nauta MH, Scholte WF, et al. Internet-based behavioural activation with lay counsellor support versus online minimal psychoeducation without support for treatment of depression : a randomised controlled trial in Indonesia. The Lancet Psychiatry. 2018; doi: 10.1016/S2215-0366(18)30223-2.

25. Araya R, Menezes PR, Claro HG, et al. Effect of a Digital Intervention on Depressive Symptoms in Patients With Comorbid Hypertension or Diabetes in Brazil and Peru Two Randomized Clinical Trials. JAMA. 2022; doi: 10.1001/jama.2021.4348.

26. Khan MN, Hamdani SU, Chiumento A, et al. Evaluating feasibility and acceptability of a group WHO trans-diagnostic intervention for women with common mental disorders in rural Pakistan: A cluster randomised controlled feasibility trial. Epidemiol Psychiatr Sci. 2019; doi: 10.1017/S2045796017000336.

27. Rahman A, Khan MN, Hamdani SU, Chiumento A, Akhtar P, Nazir H, et al. Effectiveness of a brief group psychological intervention for women in a post-conflict setting in Pakistan: a single-blind, cluster, randomised controlled trial. Lancet. 2019; doi: 10.1016/S0140-6736(18)32343-2.

28. Chen S, Conwell Y, Xue J, et al. Effectiveness of integrated care for older adults with depression and hypertension in rural China: A cluster randomized controlled trial. PLoS Med. 2022;doi: http://dx.doi.org/10.1371/journal.pmed.1004019
